# Evolutionary and Functional Relationships in the Truncated Hemoglobin Family

**DOI:** 10.1371/journal.pcbi.1004701

**Published:** 2016-01-20

**Authors:** Juan P. Bustamante, Leandro Radusky, Leonardo Boechi, Darío A. Estrin, Arjen ten Have, Marcelo A. Martí

**Affiliations:** 1 Departamento de Química Inorgánica, Analítica y Química Física, INQUIMAE-CONICET, Facultad de Ciencias Exactas y Naturales, Universidad de Buenos Aires, Buenos Aires, Argentina; 2 Departamento de Química Biológica e Instituto de Química Biológica de la Facultad de Ciencias Exactas y Naturales (IQUIBICEN), Universidad de Buenos Aires, Buenos Aires, Argentina; 3 Instituto de Cálculo, Facultad de Ciencias Exactas y Naturales, Universidad de Buenos Aires, Buenos Aires, Argentina; 4 Instituto de Investigación Biológica, CONICET, Universidad Nacional de Mar del Plata. Buenos Aires, Argentina; Koç University, TURKEY

## Abstract

Predicting function from sequence is an important goal in current biological research, and although, broad functional assignment is possible when a protein is assigned to a family, predicting functional specificity with accuracy is not straightforward. If function is provided by key structural properties and the relevant properties can be computed using the sequence as the starting point, it should in principle be possible to predict function in detail. The truncated hemoglobin family presents an interesting benchmark study due to their ubiquity, sequence diversity in the context of a conserved fold and the number of characterized members. Their functions are tightly related to O_2_ affinity and reactivity, as determined by the association and dissociation rate constants, both of which can be predicted and analyzed using *in-silico* based tools. In the present work we have applied a strategy, which combines homology modeling with molecular based energy calculations, to predict and analyze function of all known truncated hemoglobins in an evolutionary context. Our results show that truncated hemoglobins present conserved family features, but that its structure is flexible enough to allow the switch from high to low affinity in a few evolutionary steps. Most proteins display moderate to high oxygen affinities and multiple ligand migration paths, which, besides some minor trends, show heterogeneous distributions throughout the phylogenetic tree, again suggesting fast functional adaptation. Our data not only deepens our comprehension of the structural basis governing ligand affinity, but they also highlight some interesting functional evolutionary trends.

## Author Summary

Globins are a superfamily of widely studied and diverse globular proteins whose function is tightly related to their oxygen affinity and reactivity. Two prominent members are the well-known tetrameric hemoglobin and the monomeric myoglobin, both involved in reversible oxygen storage and transport in mammals. Truncated hemoglobins form one of the three main monophyletic branches of this superfamily, presenting an interesting paradigm for structure-function prediction studies, as a result of their well-known and conserved fold. In the present work we started from the working hypothesis that states that “it is possible to predict the function starting solely from sequence information through the determination of structure based chemical reactivity patterns related to oxygen reactivity” and predicted oxygen reactivity for over 1000 truncated hemoglobins and analyzed the results in an evolutionary context. Our results taught us many interesting and novel features of these proteins, underscoring flexibility and adaptability of the globin fold. The work also shows that it is possible to characterize protein function in greater detail if specific sequence-to-function bridges are built upon a solid structural basis.

## Introduction

Predicting function from sequence and/or structure is one of the most important goals of structural biology, especially considering the increasing number of available sequences derived from multiple sequencing projects [1]. General function assignment or annotation, typically based on similarity with sequences with known biochemical function by means of BLAST [2] or generally done through the inclusion of a given protein to a family using HMMER profiling [3], is common practice. However, determining specific functional properties or aspects, like substrate specificity/affinity or catalytic efficiency of a given protein with accuracy and detail at the residue level, is not straightforward. Even so, assuming that protein function is determined by protein structure and the particular physicochemical properties of its residues, encoded by the protein’s primary structure, it should in principle be possible to predict such functional properties in detail based on sequences and structures only.

The globin superfamily of heme proteins offers a large, diverse and thoroughly studied set of proteins, whose function is tightly related to small gaseous non polar ligand (mainly O_2_ but also NO, and CO) [4–6] affinity and reactivity. It is known that hemoglobins (Hbs) can have functions other than oxygen storage and transport, including enzymatic and sensing functions [7]. Globins, as well as other heme proteins with high O_2_ affinity such as mycobacterial truncated hemoglobins, usually function as O_2_ (and other reactive oxygen and nitrogen -RNOS- species) redox related enzymes [8–11]. Moderate O_2_ affinity globins, like the mammalian monomeric myoglobin (Mb) and tetrameric hemoglobin, usually act as oxygen carrier storage proteins [12,13], while low O_2_ affinity globins, such as soluble guanylate cyclase or the globin coupled sensors (GCS), are NO, CO or redox sensors [14,15].

**Fig 1:**
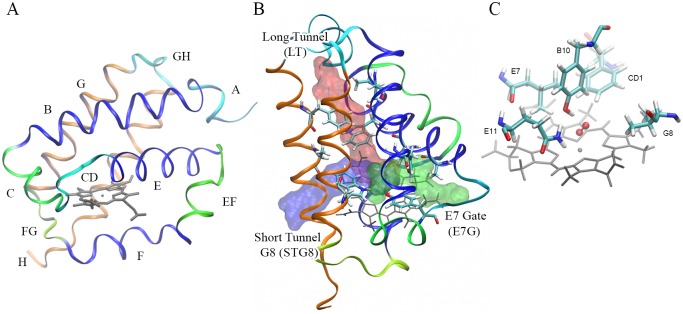
The distinctive truncated hemoglobin structure. (A) Typical fold of a trHb structure and the commonly used structural blocks (shown in different colors). (B) Schematic representation showing the three different ligand entry tunnels present in trHbs: Long Tunnel (in red), Short Tunnel G8 (in blue) and E7 Gate (in green). (C) Schematic representation of the five structural positions that define the active site in a typical trHb. The figure shows the heme group (grey), the bound oxygen ligand (red in balls and sticks) and the five key residues (shown as sticks).

The truncated hemoglobins (trHbs), also known as 2/2 Hbs, form one of the three lineages within the globin superfamily of proteins and is the only one present in all three superkingdoms of life [16,17]. They are distinguished by a simplified and unique two-over-two α-helical fold (see Fig 1A) and corresponding smaller size, i.e. 75–80% relative to three-over-three globins [17]. trHbs are organized in a number of structural blocks, as demonstrated in Fig 1A, in order to facilitate textual description and quick identification. Briefly, the protein is folded as two paired helix sandwich, composed of the BE and GH helices layers [18]. The well defined heme ligand binding site is composed of five structural positions, denominated B10, CD1, E7, E11 and G8 [17], and depicted in Fig 1C, which form distinctive features characterizing the trHbs. It should be noted that although it is tempting to analyze each residue contribution separately, these so-called distal residues act as a group in order to define the ligand reactivity. The generally accepted classification of trHbs in groups N, O and P (also labeled I, II and III) is founded on this characterization performed by Wittenberg et. al. [17] and later corroborated by a phylogenetic analysis [19]. Furthermore, trHbs present three topologically different ligand entry paths, i.e. long tunnel (LT), short tunnel G8 (STG8) and the E7 gate (E7G, the main ligand entry and escape route in three-over-three globins such as Mb), as schematically shown in Fig 1B, through which ligands can migrate from the solvent towards the protein active site.

Small ligand affinity is determined by the ratio between the association (*k*_*on*_) and dissociation (*k*_*off*_) rate constants, which characterize the corresponding processes. Association involves ligand migration from the solvent bulk to the active site through the protein gates and/or tunnel cavity systems, displacement of heme bound ligands (either external or protein residues) and finally Fe-ligand bond formation [20–23]. Dissociation, on the other hand, involves the disruption of the protein bound ligand interactions and its escape to the solvent [22,24]. During the last decade our group developed and applied several *in-silico* methods to address both the ligand association and dissociation processes with atomic resolution [5,25]. Briefly, using advanced sampling techniques we computed the free energy profiles (FEP) for ligand migration along the protein through internal tunnels that, together with the energy required to release the water molecules in the active site, account for the ligand association process. Also, molecular oxygen binding energy calculated by using hybrid quantum mechanics / molecular mechanics (QM/MM) based methods were successfully used to understand, correlate and determine the corresponding oxygen dissociation rate constants [5,26–30]. These studies showed that these *in-silico* analyses, performed in the context of available, structural and kinetic data, allow for a deep understanding of how particular globins control ligand affinity, paving the way for the development and application of a prediction protocol for the whole protein family.

Using as a working hypothesis that it is possible to predict the function starting solely from sequence information through the determination of structure based chemical reactivity patterns related to oxygen reactivity, we have developed an *in-silico* protocol in order to predict several functional properties, including the association and dissociation rate constants, for ca. 1000 trHbs sequences. This novel approach is based on the combination of homology modeling and molecular based energy calculations and further complemented with a phylogenetic analysis, with the ultimate goal to predict and analyze trHb function in an evolutionary context. Meta-analysis of the results not only deepens our comprehension of the structural basis governing ligand affinity, but it also highlights some interesting evolutionary trends in trHb function.

## Results

The results are organized as follows. A revision of the trHbs phylogenetic tree and analysis of (group based) conserved residues compose the first section, followed by sections describing the kinetic association and dissociation process and the prediction of the corresponding rate constants. Structural and functional property predictions in the whole trHb protein family form the fourth section. Finally, a global analysis of the computed properties is presented in a phylogenetic context.

### Revision of the trHbs phylogenetic tree and analysis of (group based) conserved residues

**Fig 2:**
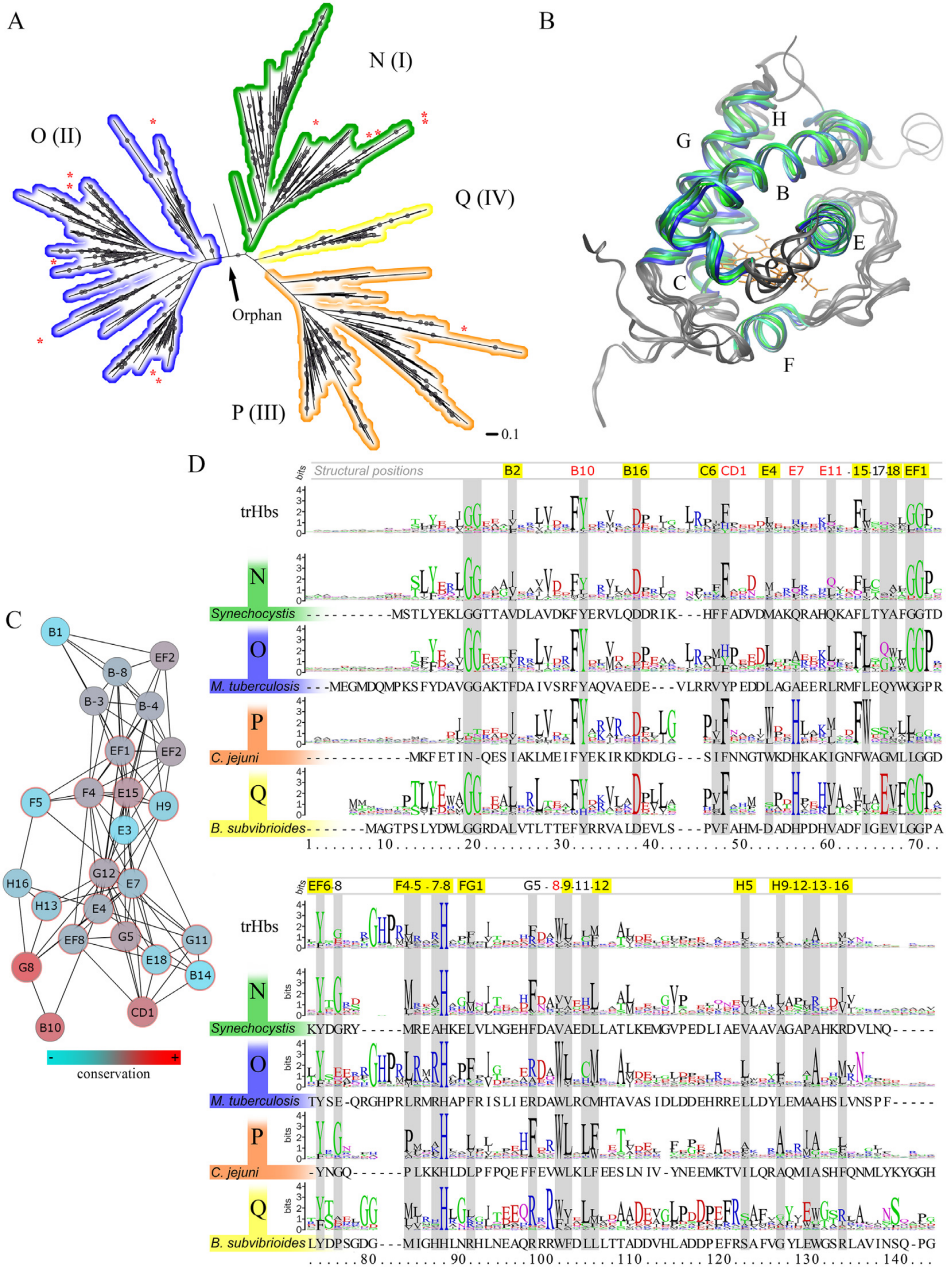
Phylogenetic hallmarks of trHbs sequences. (A) Bayesian phylogenetic tree of trHbs family presented as a radial phylogram. Dots indicate bayesian support of ≥80%. The scale bar represents a distance of 0.1 accepted amino acid substitutions per site. Red asterisks indicate sequences with resolved 3D structures that were used in the construction of both the multiple sequence alignment (MSA) used for phylogeny and the structural alignment presented in (B). (B) Structural alignment of 17 available trHbs sequences. The selected color regions, together with helix labeling, correspond with the most conserved regions in the MSA. (C) MI network of positions that have cMI > 65. Specificity determining position (SDP) E7 and its direct neighbors are highlighted. Color of the node is indicative for the Kullback-Leibler information. Red encircled nodes correspond with the central SDP, E7, and its direct neighbors. (D) Logo sequences trimmed with block mapping and gathering with entropy (BMGE) for all 1107 considered trHbs and for each separate group. One sequence member of each subgroup is also shown as a reference. Known hallmark and other important positions are shaded in grey with their structural position denominators indicated above, in red for active site residues and with yellow shading for identified SDPs.

The trHb family phylogeny has previously been described through the well known clustering into clusters N, O and P, also referred to as I, II and III, as derived from an analysis of 111 sequences available ca. 10 years ago [19]. Since then, many new sequences have become public, which allows for a more elaborate analysis. HMMER profiling was used to screen UnitProt and the PDB database and a non-redundant selection of the obtained sequences were aligned with Promals3D, which incorporates structural information. This multiple sequence alignment (MSA) was used to obtain a novel, bayesian tree (Fig 2A) that contains 1107 sequences (see S1 Fig to see where each protein ends-up, labeled with its corresponding UniProt ID). The tree largely corroborates the current classification, with the N group harboring 24%, the O group 45% and the P 27% of the sequences. However, the topology also suggests the existence of a novel, small (4%), clade of sequences labeled as Q (or IV) (see S2, S3 and S4 Figs to see where each protein ends-up for N, O, P and Q groups, respectively, labeled with its corresponding UniProt ID). Recently, Vinogradov and coworkers revised the phylogenetic relationships of bacterial and archeaeal globins. Their results are similar as those presented here, showing the presence of the N, O and P clusters with similar percentages of sequences and no cluster assignation discrepancies [7].

The taxonomic distribution shows as expected a broad range of phyla within the eukarya, bacteria and also archaea super kingdoms for the N group, emerging few sequences corresponding to plant sequences (3%), whereas P and Q contain only bacterial sequences, and the O group hosts bacterial as well as 4.2% plant sequences. Although there are proteins displaying no more than 15% of sequence identity, overall structure is well conserved (Fig 2B). This structural conservation makes it feasible to develop a protocol for a complete family characterization based on a sequence–structure–function relationship.

To identify key residues that determine trHbs properties and subgroup characteristics we analyzed the information derived from the corresponding sequence logos, as well as Cluster and Specificity Determining Positions (CDP and SDPs, respectively) and Mutual Information (MI) analysis (see Methods section for details). The results, presented in Fig 2C and 2D and S1 Table, yield a lot of information about the relevance of each structural position. Fig 2C, for example, shows the network of all positions with a cMI higher than 65 highlighting E7 and its direct MI-neighbours (most of which are also SDPs), which compose active site, heme binding and key structural positions. Hence, SDP analysis suggests that active site and heme binding residues are the main driving force of functional diversification. As expected, data conclusively shows the strictly conserved HisF8 that coordinates the heme group, whose binding is also supported by the basic E10, and E4, EF6, F4 and H16 residues building the heme hydrophobic environment. Two GG motifs, the first between the A and B helices and the second at the end of the E-helix (starting at EF1) are conserved in groups N, O and Q but are highly variable in group P. A highly conserved AspB16 marks the end of B-helix and is important for the typical trHb fold.

Concerning residues that allow group specific characterization, structural positions E7, with a conserved His defining the E7 gate (see below), and E15, governed by a Trp in group P, allow to discriminate P from O and N, hence, should be considered P specific hallmarks. Group O shows a characteristic basic stretch (His-Pro-Arg-Leu-Arg-X-Arg) located in the EF loop and the first turn of the F helix. Key characteristics of new identified Q group are the above mentioned GG motifs, Trp or Phe at G8 and an almost 100% conserved HisE7, as well as novel defining unique characteristics such as a strictly conserved Glu at E17, an Arg-X-Arg motif close to the G8 position and a rather distinct, conserved C terminus including the highly conserved Ser at structural position H22, all of which should be considered Q specific hallmarks.

Active site, or distal, residues show intermediate global conservation and also contribute to the group clustering. Most conserved is the commonly found PheB9-TyrB10 key for determining ligand affinity. G8 has the typical Trp in O, P and Q. CD1 is predominantly a Phe, but the O group also shows Tyr or His. E7 and E11 show higher levels of variation (Fig 2D).

It should be noted that there are four additional structural positions with significant MI values, i.e. E17, EF8, G5 and G11, far away from active site or tunnels topologies, which molecular function is currently unknown (highlighted in red at Fig 2D). They could be related with trHb fold stability, protein-protein interactions and/or post-translational modifications. A final point of notice is related to the sequence length, since many proteins show extended N or C terminal segments. Available structural data suggest that although they may adopt secondary structure, it does not alter the global protein fold.

### trHbs association rate (k_on_) is determined by tunnel topologies and active site water displacement

In order to be able to predict ligand affinity from the primary sequence only, reliable calculations of the various variables involved needed to be developed. The employed strategy used to compute the association rate constants (*k*_*on*_) is based on the determination of the free energy profiles (FEP) for ligand entry and the use of a kinetic model to obtain the corresponding *k*_*on*_. The kinetic model is based on the works of Olson, Viappiani and colleagues [21,22,31,32], which considers two basic processes: the partition of the ligand or the equilibrium between the solvent and the tunnel cavities (or wells), and the migration from the tunnel to the active site. The first is determined mainly by the depth of the free energy wells, and thus the barrier to escape back to the solvent, while the second is determined by the barrier that the ligand needs to cross to move forward and reach the heme. Qualitatively, in this model faster rates are achieved combining a deep well that increases the effective ligand concentration inside the protein, and a small barrier to reach the heme.

**Fig 3:**
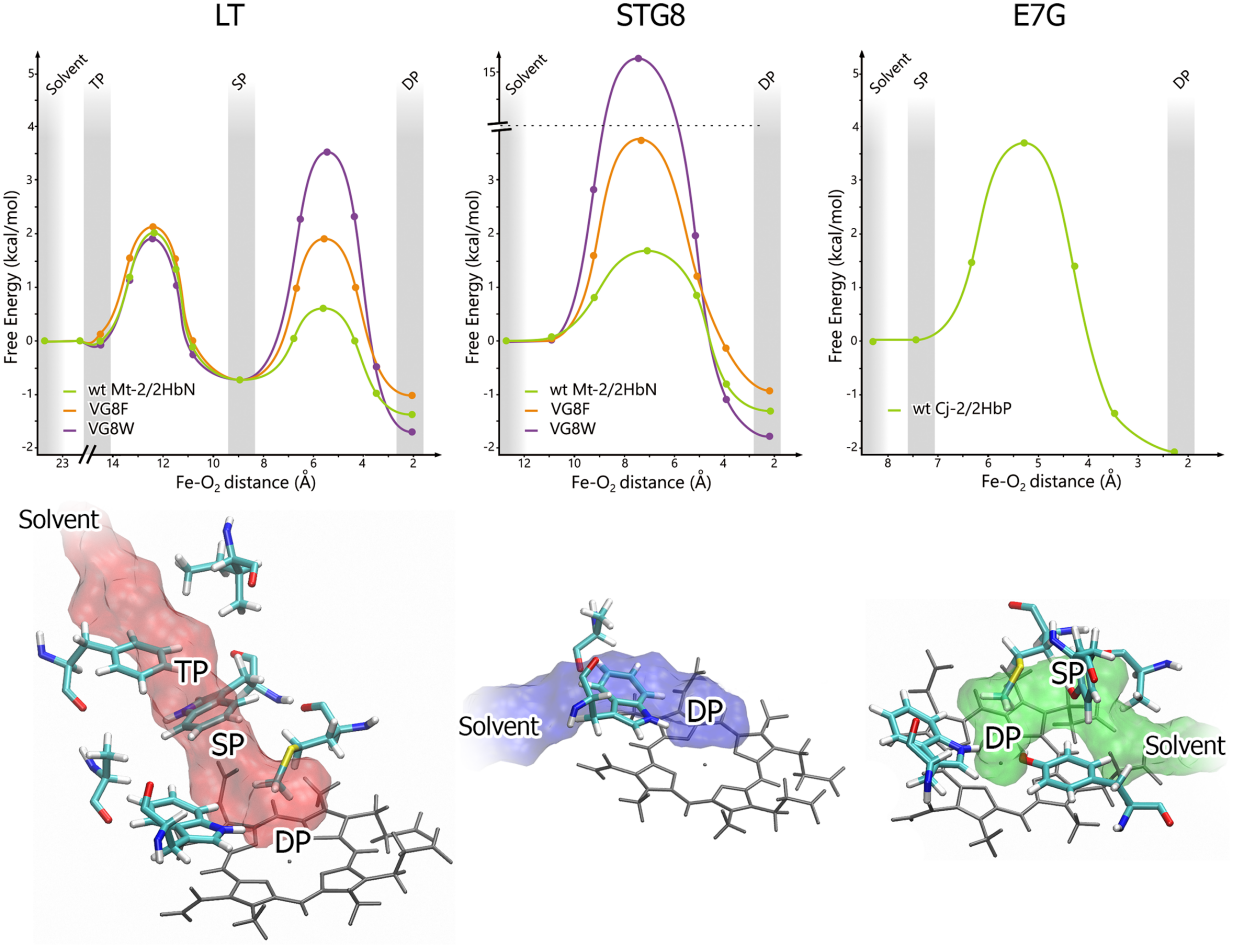
The three topological tunnels in trHbs. Top: examples of free energy profiles (FEPs) along the three potential ligand migration paths for Mt-trHbN Long Tunnel (LT), Short Tunnel G8 (STG8) and Cj-trHbP at E7 Gate (E7G). DP, SP and TP correspond to distal, secondary and tertiary pockets, respectively. The x coordinate is defined by the Fe-O_2_ distance through the pathway. Bottom: schematic representations of the heme distal residues, the tunnel and cavities system estimated with implicit ligand sampling for LT, STG8 and E7G.

As mentioned previously, trHbs present three potential paths for ligand entry and exit, LT, STG8 and E7G (see Fig 3). The LT runs parallel to the trHbs fold longer axis (along the H-helix) and perpendicular to the heme plane. It exits the protein between helices Q and H. It was described in Mt-trHbN, and always shows the presence of three wells and two barriers. The key residues defining LT topology (i.e. barrier height or well deepness) are H5, B2, H9, E15, E11 and G8. The STG8 is oriented perpendicular to the LT, runs parallel to the heme towards the G-helix, and exits the protein between helices G and H. Key residues determining its FEP are H9, G8 and G9. It was also first described in Mt-trHbN, and shows the presence of two wells and only one barrier. The third entry path, the E7G, is topologically equivalent to the ligand entry site in Mb. It was first described in Mt-trHbO and Cj-trHbP. It runs parallel to the heme plane in the opposite direction with respect to the STG8. It presents 2 wells separated by one barrier, and the residues determining their characteristics are B10, CD1, E7 and E11.

As shown by the examples in Fig 3, size, shape and hydrophobicity of the residues lining the tunnels determine both the barrier heights and well depth along the corresponding FEP. Usually, barriers in the 1–3*kcalmol* ^−1^ range are considered small and correspond to -and will be referred as-open tunnels, while large barriers in the 10–20*kcalmol* ^−1^ range result -and will be described as- blocked or closed tunnels. Well depths are computed relative to the bulk solvent and can be as large as 3*kcalmol* ^−1^ resulting in 150 times enhanced effective ligand concentration, which thus increases the association rate accordingly as shown for human Mb [33].

To analyze the reliability of our proposed model we first computed the FEP profiles along each tunnel for all trHbs whose k_on_ rates were determined experimentally, thus determining for each protein all three tunnel contributions to the ligand association process, which we will refer to as *k*_*LT*_, *k*_*STG8*_ and *k*_*E7G*_. The linear combination of the three tunnel rates in each protein, finally results in the corresponding trHb tunnel dependent association rate (*k*_*tunnels*_). It is important to note that *k*_*tunnels*_ range is usually dominated by the most open tunnel. In other words, once a tunnel is open, resulting in a high rate (10^5^ *M* ^−1^ *S* ^−1^), having a second similarly opened tunnel, does not result in a significant increase in *k*_*on*_.

Interestingly, and as analyzed in detail in Bustamante et. al. [34] *k*_*tunnels*_ shows poor correlation with the experimentally determined *k*_*on*_ (R^2^ = 0.47) and rates are significantly overestimated. Analysis of wt versus mutant pairs, where tunnel topologies are not altered but nonetheless result in over ten times difference in *k*_*on*_ values, combined with literature data strongly suggested that water displacement, from the distal pocket on top of the heme required to allow oxygen coordination, is the missing factor [20–22,35]. To account for this effect, the water stabilization free energy for each protein as estimated and characterized by the corresponding equilibrium constant $K_{H_{2}O}$ (presented in S2 Table for all possible trHbs).

**Fig 4:**
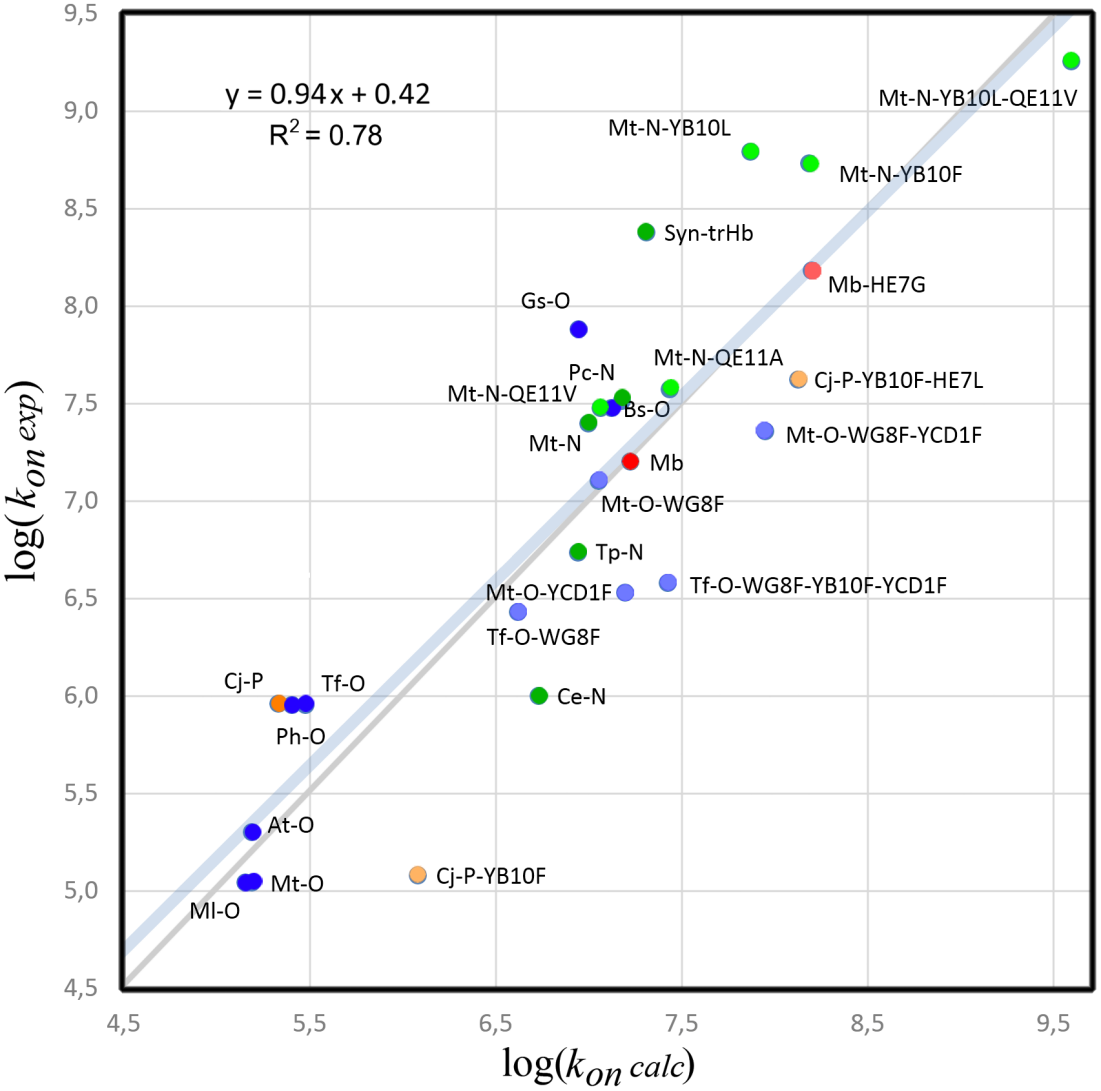
Plot of *log*(*k*_*on*_ *exp*) vs *log*(*k*_*on*_ *calc*). Dots indicate Mb in red and the trHbs in green, blue and orange for groups N, O and P, respectively. Dots with lighter colors indicate mutant proteins. Identity line is shown in grey. A complete list of experimental and calculated values is available at S3 Table.

To demonstrate the roles played by *k*_*tunnels*_ and water displacement, pairs (or groups) of proteins where one of the contributions varies while the other remains fairly constant, can be compared. For example, single and double distal GlnE11Val/Ala and TyrB10Leu/Phe mutants of Mt-trHbN can be compared wt Mt-trHbN (Fig 4). The substitutions do not alter tunnel topologies but both wt residues are capable of establishing hydrogen bonds with the water blocking the heme iron. Exchanging each of them for hydrophobic residues results in a significant (almost 10 times) increase in *k*_*on*_. Moreover, the double mutant, where there are no more water stabilizing interactions, results in an even larger increase in *k*_*on*_. To show the role played by the tunnel, we can look at the ValG8Phe mutant of Mt-trHbN, which has a 10 times smaller *k*_*on*_ compared to the wt [36] having very similar distal site residues, and thus similar $K_{H_{2}O}$. However, the ligand needs to overcome higher barriers along the tunnel to reach the active site, mainly due to the size increase of G8 residue (see Figs 4A and 3B), a fact that is reflected in smaller *k*_*tunnels*_. Another example, of the role played by the tunnel, is obtained when comparing wt forms of Mt-trHbN and Cj-trHbP, which show that the former has about 15 times larger *k*_*on*_ (Fig 4). In these cases, the ligand enters through different tunnels, STG8 and E7G, respectively, and which as shown in Fig 3A and 3C, present significantly different barrier heights, explaining the observed trend.

In summary, the above examples clearly show the relevance of both factors in determining the overall association rate. The resulting values (*k*_*LT*_, *k*_*STG8*_, *k*_*E7G*_ and $K_{H_{2}O}$) computed for all possible trHb sequences are presented in S2 Table and will be analyzed later. Combining both $K_{H_{2}O}$ and *k*_*tunnels*_ using eq 4 (see Methods), we were able to have a better estimate of the association rate, which shows good correlation (R^2^ = 0.78) with experimental data (Fig 4).

### trHbs show moderate to low oxygen dissociation rates (k_off_) as determined by active site hydrogen bonds

**Fig 5:**
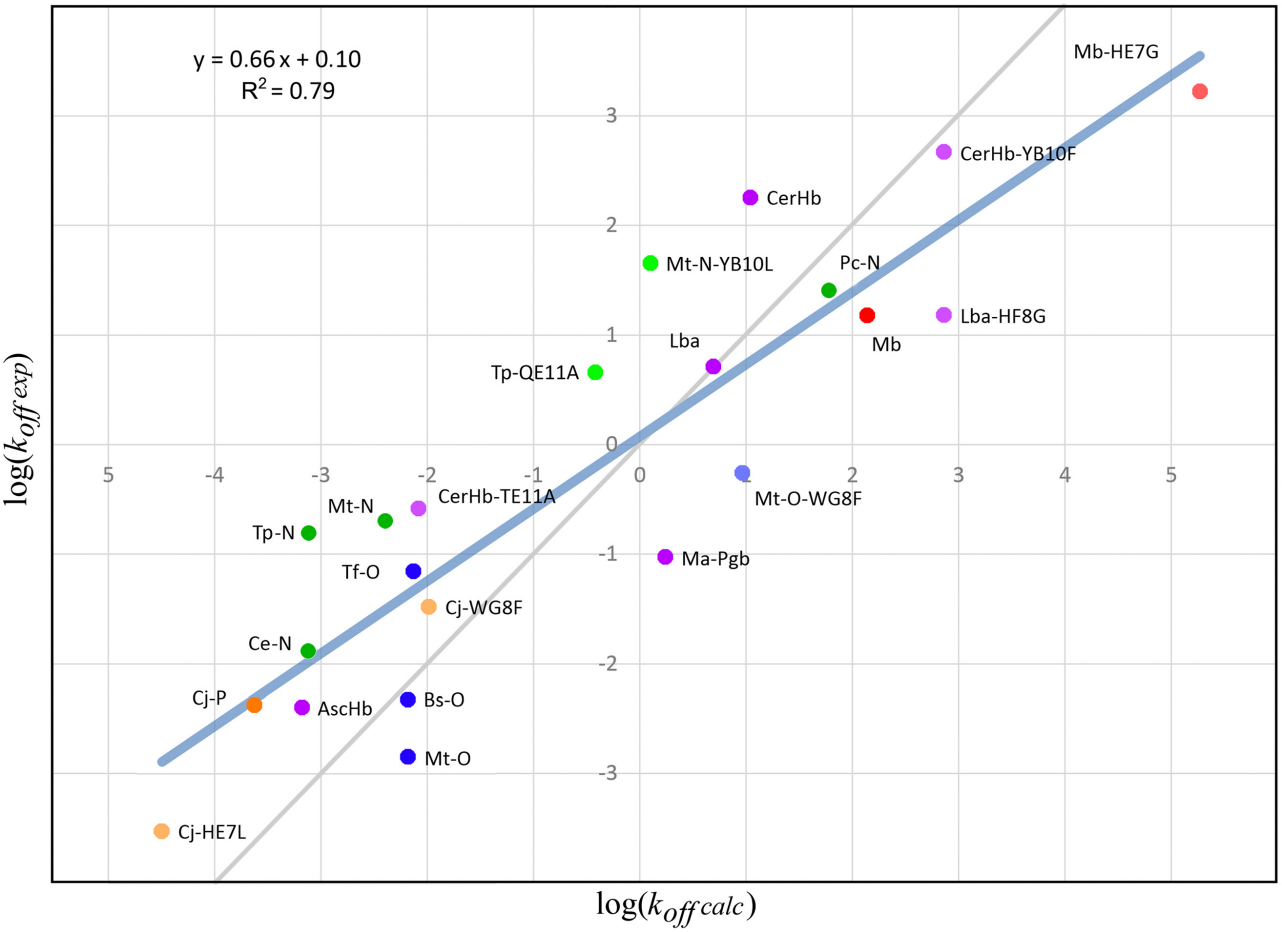
Plot of *log*(*k*_*off*_ *exp*) vs *log*(*k*_*off*_ *calc*). Dots indicate Mb in red and the trHbs in green, blue and orange for groups N, O and P, respectively. Other globin forms are indicated in purple. Circles with lighter colors indicate mutant proteins. Identity line is shown in grey. A complete list of experimental and calculated values is available at S4 Table.

As shown by previous works from our group, oxygen dissociation is mainly controlled by the strength of distal interactions [5]. Here a similar approach as described above for *k*_*tunnels*_ was followed. First, the oxygen binding energy ($\Delta \Delta E_{O_{2}}$) was calculated for a number of trHbs with resolved structure using a QM/MM scheme and compared to actual empirical data (see Methods). The corresponding plot of the predicted vs experimental determined *k*_*off*_ values for all available wt and mutant trHbs (and some additional cases from our previous works) is presented in Fig 5. This shows that trHbs *k*_*off*_ prediction is, on average, as good as the predictions for *k*_*on*_ (R^2^ = 0.79). The computed *k*_*off*_ values for all trHbs are presented in S2 Table.

Global analysis of the obtained values, suggests that three functional groups can be identified. A first group corresponds to proteins displaying fast dissociation rates (100*s* ^−1^), usually due to a lack of ligand stabilization by distal residues. Proteins with moderate dissociation rates, with half-lives of the oxygenated species in the seconds timescale form a second group, where those with low or very low dissociation rates, which usually result in high -or very high- oxygen affinities reside in a third group. Strikingly, ca. 70% of all analyzed cases display a low koff, as that observed for the trHbs N, O and P from Mycobacterium tuberculosis (Mt-trHbN), Thermobifida fusca (Tf-trHbO) and Campylobacter jejuni (Cj-trHbP), 25% showing a predicted moderate dissociation rate like that observed for Pc-trHbN or human Mb with the remainder showing high or very high rates. The structural reasons underlying this observation is the invariable presence of at least one (and many times two) strong hydrogen bonds (to the ligand) forming residues, like TyrB10 (present in 80% cases), TrpG8 (70%), His or Tyr at CD1 (20 and 15%, respectively) and GlnE11 (21%). Moreover, in many cases, like the already characterized Cj-trHbP, there are several hydrogen bonds forming residues that act cooperatively and dynamically establishing a tight multiple hydrogen bond network with the bound ligand, resulting in a very low *k*_*off*_.

It is important to note that the contribution of the FEP along the tunnels for the ligand escape process and thus *k*_*off*_ is negligible in trHbs, and was thus not considered (see Methods). As for *k*_*on*_, the functional and phylogenetic implication of the predicted dissociations rates will be discussed below in the context of the other computed properties.

### Predicting kinetics over the whole family

The present work’s ultimate goal is to make a potential functional prediction for each member of the trHb protein family in an evolutionary context, based solely on sequence information. To achieve this task, we constructed simplified models of most possible trHbs, in which the particular tunnel and/or distal residues were exchanged in a group dependent reference structure (1IDR for group N, 2BMM for group O and 2IG3 for groups P and Q, see Methods for details).

Analysis of all possible tunnel residue combinations, using the MSA, shows 460, 156 and 137 different residue combinations that define respectively the LT, STG8 and E7G characteristics. Selecting the most representative combinations while combining similar residues in the same group (see Methods) resulted in 41, 36 and 17 different residue combinations that cover more than 87% of all possible trHbs. The other structural aspect to be considered, are the active site residues that interact with coordinated O_2_ and water by means of hydrogen bond interactions. The five topological positions (B10, CD1, E7, E11 and G8) in the active site of the trHbs work cooperatively to define ligand stabilization. Analysis of the MSA shows that there are 158 different combinations of these key residues, which can be trimmed down to 28 combinations that cover over 75% of the trHbs active sites.

Once all possible combinations that define the tunnel and active site structural characteristics for the whole trHb family were determined, the corresponding models were built and used to compute the FEP for each possible residue combination in each of the three tunnels and the number of hydrogen bonds retaining the water at the active site that determine the *k*_*on*_ and the QM/MM obtained $\Delta \Delta E_{O_{2}}$ that determine *k*_*off*_ (see Methods). As a control, we also built this simplified models for all trHbs of which a complete structure is available and the results for the obtained parameters and kinetic rates are equivalent, suggesting that the approach is appropriate. The resulting values for *k*_*LT*_, *k*_*STG8*_, *k*_*E7G*_, $K_{H_{2}O}$, *k*_*on*_, $\Delta \Delta E_{O_{2}}$ and *k*_*off*_ for each determined residue combination and thus each analyzed trHb are presented in S2 Table. It is important to note that in our approximation trHbs with the same key position residue combination will display exactly the same rate constants. The calculated values should be considered a first estimation that according to the presented results is sufficiently accurate -typically well within one order of magnitude- to infer structure-function relationships. Clearly, the predicted values will differ from real values mostly since the characters are further modulated by minor aspects that are not considered in the calculation.

### Global analysis of the kinetic rates and the oxygen affinity

A first look analysis at the distribution of association rate values for all computed trHbs (S5 Fig) shows that although a wide range of values is possible, most trHbs display values in the 10^5^–10^8^ *M* ^−1^ *s* ^−1^ range, consistent with a tunnel which accesibility is only hampered by a water molecule. There are also a significant number of proteins that display values up to 10^9^ *M* ^−1^ *s* ^−1^, which is caused by the absence of blocking water. Finally, a minor group of proteins with association rates in the 10^3^–10^4^ *M* ^−1^ *s* ^−1^ range exist, which corresponds to those proteins where tunnels are blocked and/or tightly bound water blocks the access to the heme. The distribution of dissociation rates is more homogenous, but with a predominance of values below 1. The range extends from values as low as 10^4^ *s* ^−1^, which corresponds to proteins binding oxygen tightly with several hydrogen bonds, to 10^4^ *s* ^−1^ for those proteins displaying highly hydrophobic distal pockets.

The reliable prediction of both association and dissociation rate constants for all types of trHbs finally allows us to determine properly their oxygen affinity, which is usually expressed as p50, the oxygen pressure that results in half the protein loaded with O_2_. The results (S6 Fig) show that most trHbs display low (or very low) p50 values (< 1mmHg), which would indicate that the protein is oxygenated even in microaerobic environments. These proteins usually display a moderate *k*_*on*_ (in the range where most values are found) and large variations in *k*_*off*_, although always displaying values below 1*s* ^−1^. There is a second group with moderate p50 values (1-5mmHg), which possibly reflects these proteins are involved in oxygen transport. Which is characterized by the presence of both moderate *k*_*on*_ and *k*_*off*_ values (between 1*s* ^−1^ and 100*s* ^−1^). And finally, there are a few members with very high p50 values (displaying mostly both large *k*_*on*_ and *k*_*off*_ values), which suggest they are unable to bind oxygen at all. Correlation analysis of p50 vs *k*_*on*_ and *k*_*off*_, suggest that p50 is predominantly controlled by *k*_*off*_ (R^2^ = 0.60), with *k*_*on*_ having little impact (R^2^ = 0.05).

### Combination of phylogenetic and oxygen binding properties analysis

**Fig 6:**
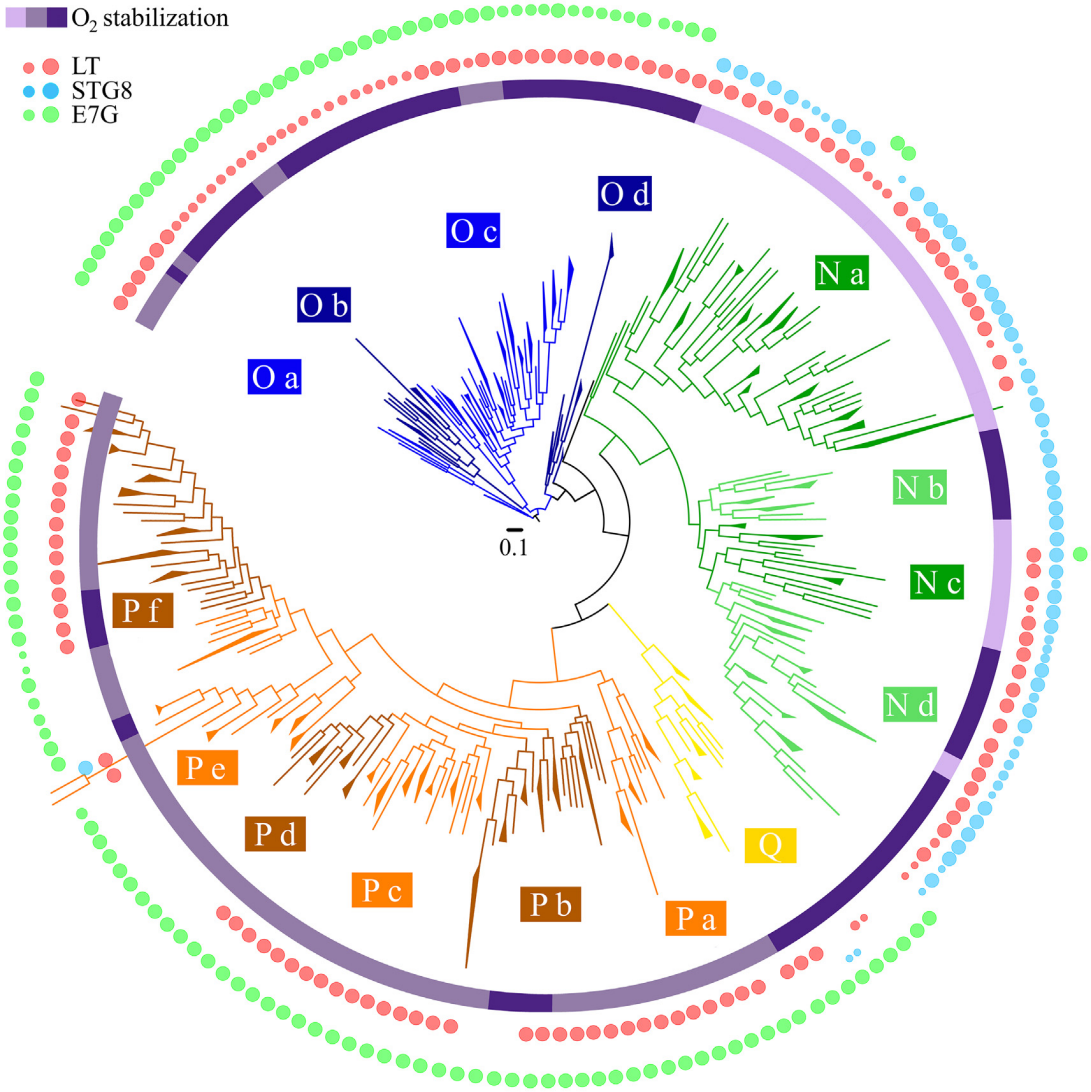
Collapsed phylogenetic tree of trHbs annotated with their key physicochemical characteristics. The circular phylogram shows the topology derived from Fig 2A, with color-matched boxes indicating hierarchical clustering. $\Delta \Delta E_{O_{2}}$ values are mapped on the inner concentric circle using a violet color gradient (greater stabilization, darker violet). Tunnel apertures are mapped as red, cyan and light green dots for Long Tunnel, Short Tunnel G8 and E7 Gate, respectively, on the other concentric circles. Dot size indicates tunnel openness.

To analyze how the different oxygen binding properties are related to the evolutionary processes that resulted in the functional diversification of the trHb family we decided to combine the above computed functional parameters for all trHbs in a phylogenetic context. To understand the resulting pattern we analyzed first, how phylogeny results in the hierarchical clustering of the trHb sequences (at the group and subgroup levels), and second, what properties co-cluster within each clade. Fig 6, thus shows the phylogenetic tree of the whole trHb family, together with a mapping of the O_2_ stabilization and the openness of each tunnel. A similar analysis was performed for the other computed parameters (S7 Fig), yielding similar conclusions. The emerging picture not only allows to further characterize each group, but also to identify several subgroups (mono- or paraphyletic) which share several key properties related to their ligand binding properties, and thus their putative functions, as will be shown in the discussion.

Oxygen stabilization (assigned as high, moderate and low), for example, shows a clear subgroup distribution that points to the groupwise conservation of key residues that determine oxygen stabilization in particular clades. Tunnel openness also shows clear group preferences. The STG8, for example, is only open in the N group. On the contrary, E7G, which is mostly blocked in group N -except for a small lineage denominated Na-, is always open for trHbs in group O as well as in P, albeit with minor exceptions. It is also interesting to note that, due to conservation of the overall fold, all the tunnels are present in all proteins. However, as shown for example by E7G in group N, it can be completely blocked (displaying free energy barrier for ligand entry of over 10*kcalmol* ^−1^) by the presence of key residues (see S2 Table).

Reasons underlying blockade of STG8 in trHbs from groups O, P and Q (and also some members at the Ia clade), can be traced to the ubiquitous presence of Trp or Phe at the structural position G8.

Clade N is particular in that it has trHbs that have either high or low but no intermediate O_2_ affinities, caused mostly by very low *k*_*off*_. Low affinity is correlated with the hydrophobic Leu at structural positions E7, E11 and G8. As such, clade N can be divided in subclades Na, Nb, Nc and Nd, of which Nb and Nd are monophyletic clades with high affinity, correlated with polar Gln at E7 and E11. A single mutation (CTA → CAA or CTG → CAG) might explain for the large change in *k*_*off*_. Nb differs from the other subclades in that it has one instead of two open tunnels. Clade O, with a fully opened E7G as defining characteristic, can be subclustered in four groups, Oa and Od being paraphyletic and Ob and Oc being monophyletic. It consists of a number of moderate and many low *k*_*off*_ trHbs with open E7G and LT. The main difference between O members is the dissociation rate, which together with the CD1 identity, which is otherwise occupied by a His or a Phe/Tyr. Group P, which contains mostly proteins with an open E7G, can be divided into six subgroups, having three monophyletic (a, d and f) and three paraphyletic (b, c and e), in general almost all subgroups present moderate kinetic constants, excluding Pb, with moderate but also very low *k*_*off*_. Finally, Group Q presents only one monophyletic subgroup characterized by moderate *k*_*on*_ and very low *k*_*off*_ values.

## Discussion

In the present work we have performed an updated and detailed phylogenetic and structural analysis of all (or most) available trHbs, looking for the structural reasons that govern their function. To achieve this task we used simplified models for 80% of all combinations of particular tunnel and distal residue substitutions, using three reference structures, one selected for each of the N, O and P groups. We predicted the interactions with the heme bound ligand that determine oxygen dissociation rates, as well as the free energy profiles for ligand migration across the tunnel/cavity system that determines the ligand association rate (the corresponding values for all computed rates are presented in S2 Table). In order to draw a general picture of trHbs evolution and function, a summary that integrates all our results is reported below.

### What are the underlying structural reasons of trHb ligand affinity?

Our working hypothesis was that determination of the proper physicochemical characteristics as derived from protein structure would allow us to infer (or predict) key trHb functional properties (uptake and release of O_2_) and associated parameters(*k*_*tunnels*_, $K_{H_{2}O}$ and $\Delta \Delta E_{O_{2}}$). The herein presented results show that we are able to predict both rates quite accurately, thus encouraging the performance of a complete analysis of all trHbs possible structures. The global analysis taught us that trHbs show, in general, moderate to very low oxygen dissociation rates, and thus moderate to high oxygen affinity, due to the presence of at least one and usually several hydrogen bond interactions between the ligand and the protein, most commonly provided by TyrB10, TrpG8, His or Tyr at CD1 and GlnE11, as was also previously found experimentally for some particular cases [22,26,37–41].

Concerning the tunnels, our results show that size and hydrophobicity of residues lining the tunnels results in the presence of deep wells along the FEP (or secondary docking sites) which increase the rate, while they reduce the rate through the imposition of sterical free energy “barriers”. Most important, almost all trHbs have at least one “open” tunnel, and many have two. It is important to note, that our data also suggest, in agreement with previous experimental observations on directed mutants [42,43], that the presence of more than one tunnel is redundant in terms of ligand association rate, since the ligand will reach the heme (and wait there to bind) through the tunnel presenting the easiest access.

In this scenario, the question of what is the relation between the presence of multiple tunnels and the trHbs function, must go beyond simple determination of *k*_*on*_ and involve also other aspects or possibilities (see below). Finally, we also show that association rate is significantly influenced by the presence of water molecules on top of the heme that interact with the distal residues through hydrogen bond interactions. The tighter the water is bound, the lower the *k*_*on*_.

### Going from structure to function

Having determined and analyzed the ligand binding properties of all trHbs and their phylogenetic relationships, the question now arises as to whether it is possible to infer or predict a possible function for them. The question of globin, and thus trHbs function is a controversial issue, since even for the hallmark protein Mb several functions (O_2_ storage, nitrite reductase, NO dioxygenase) have been proposed and shown to be possible [10,22,44]. The problem arises due to the vast heme reactivity that allows it to fulfill different tasks under different conditions. However, not all tasks will be performed with the same efficiency due to the differential heme reactivity, thus some functions may seem more likely than others. Moreover, as mentioned in the introduction, the ubiquitous presence of molecular oxygen in the environment and the large variation observed in heme protein's affinity towards it (in opposition to CO or NO that bind tightly to the heme almost independently of the protein environment), allows to draw some general lines based on the key parameters of O_2_ association and dissociation, computed here, even although, to the best of our knowledge, there is no single trHb whose function has been undoubtedly established and that are many trHbs remain poorly characterized beyond basic ligand kinetics. For some of them, which will be used here as leading cases, tentative but well based functional assignment is available. Finally, it is important to note that the predicted affinities apply only to “hypothetical” monomeric isolated trHbs *in vitro*. A such, when predicting possible trHb functions starting from the computed properties, we do not consider several issues like quaternary and cooperative effects; protein localization and interaction with other proteins or membranes; and the particular circumstances of each organism living (e.g. aerobic/anaerobic, type of metabolism), which provide the proper context. Therefore, our predictions should be taken as a starting point or working hypothesis to further study each trHb function in a biological relevant context, in a similar manner as what is done with *in vitro* kinetic measurements.

Possibly the most studied trHb is Mt-trHbN, paradigm of the N group trHbs. This protein likely function is to detoxify NO through its oxidation to nitrate by the oxy heme. To fulfill this task, a high oxygen stabilization is required, and the presence of multiple tunnels is likely an important factor [35,45–48]. Most of the Nd subgroup proteins share these properties, and thus NO detoxification seems a likely function. Interestingly, two others subgroups of group N (Na and Nc) show a larger *k*_*off*_ and thus reduced oxygen affinity more similar to that of Pc-trHbN or Mb cases [22,49]. For these cases, as well as other trHbs sharing a large *k*_*off*_ and presence of one or two open tunnels, a role involving oxygen storage or transport seems more likely, since a moderate *k*_*on*_ and moderate to large *k*_*off*_ is a prerequisite to allow efficient oxygen uptake and delivery.

Another case, could be represented by Tf or Mt trHbO, paradigms for group O trHb, which have been proposed to work in relation with reactive oxygen species, in catalase-peroxidase like functions [50,51]. Key properties of these proteins to perform these tasks are the presence of a tight distal hydrogen bond network, revealed in a low or very low oxygen *k*_*off*_ and the presence of an open E7G that provides shorter and more polar access to the heme than STG8 and LT and could thus be particularly suited for the entry/escape of polar or charged ligands such as superoxide. Also noteworthy is that heme proteins performing these tasks usually display polar aromatic (Trp-Tyr-Arg-His) residues in their active sites that can participate in redox reactions stabilizing free radical species. In correspondence with this idea, Wang and coworkers [11] recently showed that a trHb from *Roseobacter denitrificans* -which belongs to the Oc group- has peroxidase activity. Although they did not analyze ligand binding properties, the presence of TyrB10, TyrE7 GlnE11 and TrpG8 suggest low *k*_*off*_ and open E7G, consistent with our proposal. These functions thus emerge as likely candidates for many (even most) group O (or II) trHbs sharing the mentioned properties.

Less is known concerning members of the P group, the best characterized member being Cj-trHbP. Although its function is not clear, it displays structural and ligand binding properties that reveal a tight hydrogen bond network and the presence of E7G (like previously described trHbO). These properties however are not shared by all group members and high variability in terms of ligand interactions and tunnel openness is revealed, preventing a general prediction about their function. The reason that P trHbs forms a distinct clade is explained by strict conservation of HisE7.

### trHbs organisms based functional distribution

Given the hierarchic clustering of the trHbs and taking into account their functional key characteristics (*k*_*on*_, *k*_*off*_, p50 and tunnel openness) we can now analyze trHb distribution in their hosting organisms. The 1107 trHbs genes belong to over 600 different species, with most of them (73%) harboring only one type of trHb, 23% displaying two different trHbs, and a few organisms more than two. Analysis of the phylogeny shows that for those organisms displaying two types of trHbs, almost half of them have an O and N types, ca 40% an O and P types of trHbs, and only about 15% N and P types together. These results are similar as those observed previously by Vuletich et. al. [19] and seem to point out that O is the ancestral group.

Based on previous description a rough functional assignment of trHb was performed by defining an NO/O_2_ multiligand chemistry type (Mt-trHbN type), an oxygen transport type (Mb-like type) and a catalase-peroxidase functional type (Tf-trHbO and Mt-trHbO type). Analysis of type related presence in each organism, shows that those species having only one trHb show predominantly a catalase-peroxidase functional type of protein (64%), followed by oxygen transport and NO/O_2_ multiligand chemistry types, both with similar populationsize (18%) which is the expected distribution based on the relative abundance of each functional type. For those organisms having two trHbs combination again reflects expected distribution. Thus, the available data does not show any evidence of functional diversification for coexisting trHbs.

We also looked for clustering of the three major types of trHbs. In group N, 57% are predicted to work in NO/O_2_ multiligand chemistry while interestingly the remaining 43% is predicted to be involved in oxygen transport. Catalase-peroxidase proposed function emerges as the likely candidate for most (86%) of group O trHbs, all sharing the mentioned properties, with the remainder being shared similarly between other functional types. Also in group P most trHbs (78%) share structural and ligand binding properties as those previously mentioned for a catalase-peroxidase like function. The remaining 22% being assigned as oxygen transport like due to their higher dissociation rate. Finally, all members of the newly identified group Q (IV) are assigned as catalase-peroxidase like. In any case, it is interesting to note that different functional types are found among the same phylogenetic group and thus care should be taken in assigning functional solely based on phylogeny.

### Final remark on trHb structural evolution

Taken together our results provide a rough evolutionary pattern of possible trHbs functions, in the sense that they were determined by the properties related to ligand reactivity, which are distributed, despite some general trends mentioned above, quite heterogeneously (or randomly) in the phylogenetic tree. This behavior could point to either functional plasticity or to high flexibility in terms of sequence-evolution to function relationships, thus resulting in multiple events of divergent and convergent evolution in terms of the studied properties, along a given evolutionary line. In other words the structural fold of trHbs seems flexible enough to allow the switch from a high affinity (or multiple open tunnels) structure to that of a low (or one/no-tunnel at all) structure in a few evolutionary steps, thus allowing for multiple rounds of reactivity/affinity and thus functional adaptation. The presence of multiple trHb paralogs in all kingdoms of life [17,52] which always appeared as a strange fact which lacked an explanation, clearly substantiates the above mentioned plasticity and evolution-to-functional flexibility and diversity.

This work also represents a proof of concept for the hypothesis that states that it is possible to infer protein function in detail -beyond family assignment- starting solely from sequence information, through the determination of key structure related chemical reactivity properties. In this context future extension of the developed methodology to other protein families, like the more structurally diverse and functionally complex 3-over-3 globins can be expected.

## Materials and Methods

### Protein sequence based phylogenetic analysis

#### Data resources and identification of trHbs sequences

Our starting sequence set was comprised by the 111 cases assigned by Vuletich and Lecomte [19] to N, O and P trHbs groups plus ca. 200 additional sequences derived and manually checked from the Pfam and PDB databases [53,54]. Separate HMMER profiles were built for each trHb group (N, O and P) by means of *hmmbuild* using default settings (HMMER Version 3.0 [55]). The complete SwissProt, Uniprot and PDB databases were then subsequently screened by *hmmsearch* using the three built profiles and default settings in order to acquire all possible available trHbs sequences. All sequences identified by the matrices with a full sequence E-value smaller than the HMMER exclusion threshold were considered as trHbs. Redundant sequences were discarded by means of CD-Hit [56] using 90% identity as upper threshold.

#### Multiple Sequence Alignments (MSA)

Multiple protein sequence alignments of all considered sequences were made using the Promals3D program [57] with default settings and including structural information considering the following seventeen PDBs which corresponds to IDs: 2BKM, 1UX8, 3AQ5, 2BMM, 1NGK, 3AQ9, 1DLY, 1UVX, 2HZ2, 1S69, 1MWB, 1IDR, 2KSC, 2XYK, 2GKN, 1DLW, 2IG3. The inclusion of X-ray structures enhances the quality of the MSA by considering key properties of the fold. The MSA was subsequently manually optimized using Jalview 2.8 [58] in order to: i) retain only sequences shorter than 160 amino acids, ii) discard sequences without the typical trHb hallmark, the conserved heme ligand HisF8, iii) manually improve of the alignment. The final MSA was checked with the X-ray structures of above cited trHbs. Finally, a total of 1107 sequences were identified as trHbs, consistent with the work by Vinogradov et. al. two years ago [7].

#### Phylogenetic analysis

Since the 1107 sequences have divergent regions and specially the terminals due to different evolutionary histories, the MSA contains blocks of poorly aligned subsequences. These were removed by Block Mapping and Gathering with Entropy (BMGE) [59], which permits selection of parts of the alignment that are suitable for proper phylogenetic inference. Trimming for phylogeny was performed with Blosum62, gap frequency at 0.2 and entropy at 0.9 resulting in a trim from 356 to 143 columns. The trimmed MSA was used to build Maximum likelihood (ML) and Bayesian phylogenies, using PhyML 3.0 [60] and MrBayes 3, respectively.

Specifically, PhyML analyses were conducted upon selection of the model using ProtTest [61] selecting the WAG model, estimated proportion of invariable sites, four rate categories, estimated gamma distribution parameter, and optimized starting BIONJ tree, with SH-aLRT branch support measures. Bayesian analysis were initiated with the ML-trees using 10 perturbations. Convergence was checked by using Awty (http://www.ncbi.nlm.nih.gov/pubmed/17766271). The resulting phylogenetic trees were viewed and edited with iTol v2.2.2 [62] and Inkscape (GNU license, www.inkscape.org).

As a control case, a phylogenetic tree was built using only the available sequences in 2006 and, as expected, we obtained the same tree’s topology reported previously [19] (S8 Fig).

#### Additional analysis

An analysis for Cluster and Specificity Determining Positions (CDP and SDPs respectively) was performed in order see if certain physicochemical aspects can be attributed to certain residues at certain positions and to include as many unforeseen aspects as possible. CDPs are positions in a protein structure, with corresponding columns in the MSA, that significantly contribute to the observed clustering and can be determined by statistical methods. SDPs are CDPs that affect function and are identified by cross analyzing the obtained data with mutual information (MI). SDPs. CDPs were determined using SDPfox [63] and Mistic [64] was used to determine MI. A table with the results of the performed SDP and MI analysis is available at S1 Table.

### Computational methods

#### Set up of the systems and classical simulation parameters

The starting structures for modeling all studied trHbs corresponds to Mt-trHbN, Mt-trHbO and Cj-trHbP crystal structures (Protein Data Bank entries 1IDR, 1NGK and 2IG3) as determined by Milani *et*. *al*. [8,65] and Nardini *et*. *at*. [66], respectively. In all cases, amino acids protonation states were assumed to correspond to physiological pH (Asp and Glu negatively charged, Lys and Arg positively charged), all solvent exposed His were protonated at the N-δ delta atom, as well as HisF8, which is coordinated to the iron heme. Since different protonation states of HCD1 could cause a different H-bond pattern related to ligand stabilization, in this case the protonation state was carefully chosen based on two aspects: i) experimental crystal structures that suggest a given H-bond pattern [67,68] and ii) correlation between computed and experimental dissociation rate constant for a given tautomer in cases where experimental data is available. Once completed, proteins were immersed in a pre-equilibrated octahedral box with ~4910 TIP3P water molecules, where the minimum distance between the protein and the extreme of the box was 10 Å. All used residue parameters correspond to AMBER ff99SB force field [69] except for the heme which correspond to those developed [70] and widely used in several heme-proteins studies from our group [5,20,26–30,71–75]. All simulations were performed using periodic boundary conditions with a 9 Å cutoff and particle mesh Ewald (PME) summation method for treating the electrostatic interactions. The covalent bonds involving hydrogen atoms were kept at their equilibrium distance by using the SHAKE algorithm, while temperature and pressure where kept constant with Berendsen thermostat and barostat, respectively, as implemented in the AMBER12 package [76]. Equilibration protocol consisted of (i) slowly heating the whole system from 0 to 300K for 20 ps at constant volume, with harmonic restraints of 80 Kcal per mol Å^2^ for all C_α_ atoms (ii) pressure equilibration of the entire system simulated for 1 ns at 300K with the same restrained atoms. After these two steps an unconstrained 50 ns molecular dynamics (MD) simulation at constant temperature (300K) was performed.

#### Homology models

All modeled variants of trHbs where built starting from the corresponding subgroup crystal structures described above, and changing the corresponding tunnel and active site residues *in-silico*. The resulting variants were equilibrated and simulated using the same protocol as used for *wild type* (wt) forms. All structures were found to be stable during the MD simulation timescale, as evidenced by the Root Mean Square Deviation analyses.

#### Oxygen migration free energy profiles

Free energy profiles (FEP) for the O_2_ migration process along the protein tunnel/cavity system were computed using the Implicit Ligand Sampling (ILS) approach [77], which has been widely used to study these process and was shown previously by our group to yield accurate results [20,36,78]. ILS calculations were performed in a rectangular grid (0.5 Å resolution) that includes the whole simulation box (i.e. protein and the solvent), the used probe was an O_2_ molecule. Calculations were performed on 5000 frames taken from the 30 ns of the production simulations. The values for grid size, resolution and frame numbers were thoroughly tested in our previous work [25]. Analysis of the ILS data was performed using an *ad-hoc* TCL program (the code is available in S1, S2 and S3 Scripts), determining in each case the magnitude of the corresponding wells and barriers scaled, so that the free energy of the ligand in the bulk solvent is set to zero.

#### Oxygen binding energy ($\Delta E_{O_{2}}$)

QM-MM calculations were performed for all O_2_ bound proteins. QM/MM methods are able to account for the active site microenvironment polarity and specific short range interactions that modulate heme reactivity and particularly the $\Delta E_{O_{2}}$. The initial structures for the QM-MM calculations were obtained from the corresponding previously described MD simulations. Selected snapshots based on the structure and dynamics analysis of the hydrogen bonds (H-Bonds) pattern for each case were selected and cooled down slowly to 0 K. Starting from these frozen structures full hybrid QM-MM geometry optimizations were performed using a conjugate gradient algorithm, at the DFT level with the SIESTA code using our own QM-MM implementation [27,70,79], with the PBE exchange and correlation functional. For all atoms, basis sets of double beta plus polarization quality were employed. All calculations were performed using the generalized gradient approximation functional proposed by Perdew *et*. *al*. [80]. Only residues located less than 10 Å apart from the heme reactive center were allowed to move freely in the QM-MM runs. The iron porphyrinate, the distal ligand and the imidazol of the proximal histidine were selected as the quantum subsystem. The rest of the protein unit, together with water molecules, was treated classically. The interface between the QM and MM portions of the system was treated by the scaled position link atom method. Further technical details about the QM-MM implementation can be found elsewhere [79]. O_2_ binding energies, $\Delta E_{O_{2}}$ [*kcalmol* ^−1^ values were calculated as:

$$\Delta E_{Prot-O_{2}}=E_{Prot-O_{2}}-\left(E_{O_{2}}+E_{Prot}\right)$$ (1)

where $E_{Prot-O_{2}}$ is the energy of the oxygenated protein, *E*_*prot*_ is the energy of the deoxygenated protein and $E_{O_{2}}$ is the energy of the isolated oxygen molecule. The oxygenated proteins were simulated in the singlet spin state, the deoxygenated proteins in the quintet spin state, and the free oxygen in the triplet state, which are the known ground states for each case. All simulations were performed at the unrestricted spin approximation. These methods have been widely and successfully used in our group to study oxygen (as well as other ligands) affinity in previous works [5].

### Determination of ligand association rates

The complete model used to determine the ligand association (and dissociation) rates using the above computed properties is thoroughly explained and validated elsewhere (Bustamante et. al. [34]) and will be presented here only briefly. The small ligand association involves two main processes, ligand migration from solvent bulk to the protein heme cavity through the tunnel cavity system, and formation of the Fe-O_2_ bond, which may involve the displacement of a water molecule from top of the heme. To estimate the tunnel contribution, with the information derived from the tunnel FEP, we used a generic kinetic model (Scheme 1) that considers the presence of several secondary docking sites (wells in the FEP) and their associated barriers [22,43].

$$Hb\;+\;O_{2}\underset{kb_{n}}{\overset{kf_{n+1}}{↔}}\;{\left(Hb\;:\;O_{2}\right)}_{n}\;\underset{kb_{\ldots }}{\overset{kf_{\ldots }}{↔}}\;\cdots \;\underset{kb_{2\cdot 1}}{\overset{kf_{2\cdot 1}}{↔}}\;{\left(Hb\;:\;O_{2}\right)}_{1}\;\overset{k_{bond}}{↔}\;\;Hb\;-\;O_{2}$$

#### Scheme 1

Generic kinetic scheme for the proposed model of O_2_ kinetics. (*Hb*: *O*_2_)_*n*_ indicate secondary docking sites for the O_2_ inside the distal pocket along the exit (entry) pathway to (from) the solvent.

Assuming a fast equilibrium between the bulk solvent and the internal protein docking sites, the ligand migration rate from the solvent to the heme active site through a given tunnel, is given by eq (2):

$$k_{t}=\frac{kf_{t}}{kf_{t}+kb_{t}}$$ (2)

where the *t* subscripts correspond to each possible tunnel (LT, E7, G8), *kf* and *kb* corresponds respectively, and according to the Scheme 1, to the rates of ligand movement towards the active site, and back escape to the solvent, which are determined from the corresponding barriers along the FEP (shown in Fig 3). Using this scheme we computed thus each tunnel dependent entry rate for all possible residue combinations defining each of the three tunnel (LT, STG8 and E7G) topologies. The complete list of computed values for all residue combinations in all trHbs is presented in S2 Table.

The three obtained constants are then combined to obtain a global tunnel dependent association rate for any given trHb, according to:

$$k_{tunnels}=k_{LT}+k_{STG8}+k_{E7G}$$ (3)

As already mentioned, it is important to account also for the presence of water molecules inside the active site and on top of the heme which block the oxygen binding process. We assume that each trHb exists in an equilibrium between a water blocked and free heme reactive state which is characterized by the corresponding state equilibrium constant $K_{H_{2}O}$ and its associated free energy $\Delta G_{H_{2}O}$, which is determined and computed as the product between $\Delta G_{H_{2}O}=2,95kcalmol^{-1}$ (taken from Bustamante et. al. [34]) and the number of hydrogen bonds that the protein established with the water in each trHb. As for the other constants, the values of $K_{H_{2}O}$ for all combinations of trHb active sites are shown in S2 Table.

For the overall ligand association process, we can assume the following mechanism:

$$Protein-H_{2}O↔Protein+H_{2}O\;\;\;\;\;\;\;\;\;\;\;(K_{H_{2}O})$$

$$\;Protein+O_{2}\to Protein-O_{2}\;\;\;\;\;\;\;\;\;\;\;\;\;\;\;\;\;(k_{tunnels})$$

If the first preequilibrium step is fast, the association reaction rate would be given by the second step rate, which is: $v=k_{tunnels}\left[Protein\right]\left[O_{2}\right]=k_{tunnels}K_{H_{2}O}\left[Protein-H_{2}O\right]\left[O_{2}\right]$, since $K_{H_{2}O}=\left[Protein\right]\left[Protein-H_{2}O\right]$ assuming solvent activity unitary. By this way, the effective *k*_*on*_ rate constant for a given protein is given by the product of *k*_*tunnels*_ and $K_{H_{2}O}$, in which *k*_*tunnels*_ can be modeled by the ligand entry barriers through all tunnels, as given in eq (3) and by using Eyring’s equation, yielding final eq (4).

$$k_{on}calc=\frac{k_{B}T}{h}\cdot k_{tunnels}\cdot K_{H_{2}O}$$ (4)

where *k*_*B*_ and *h* are the Boltzmann and Plank constants, respectively, and T is the temperature = 300K. It is important to remark that a more detailed estimation of the observed association rates, should include also an estimation of the barrier defining the oxygen to heme binding process (*k*_*bond*_). However, given that previous QM/MM studies from us and others showed that there is only a tiny barrier due to quintet to singlet spin transition and iron in plane movement, its effect is not expected to be large or change significantly among different trHbs all displaying the same proximal histidine ligand [27,81].

Another important point of notice concerning our estimation of *k*_*on*_, is that our model assumes that heme is pentacoordinated (5c), in the sense that no other protein residue binds and thus blocks the heme. In trHbs, hexacoordination (6c) has been observed in some cases, like *Synechococcus* and *Synechocystis*, in both the ferric and ferrous states [82–86]. Thus and although for other trHbs the data concerning hexacoordination is not completely clear, particular care should be taken when analyzing those trHbs displaying potential coordinating residues in the distal site, like His or Lys in position E10 [87]. For trHbs displaying a 6c state, ligand binding occurs exclusively to the 5c state, and both states are in dynamic equilibrium. Therefore, in these cases, the computed *k*_*on*_ represents an upper estimate of the “real” rate, since it assumes 5c ↔ 6c equilibrium is completely displaced to the 5c state. Generally speaking, for those proteins where the 6th ligand is loosely bound, the predicted value will be closer to the observed experimental value, while for those where there is a strong bond, the predicted value will be overestimated. As an example, for 6c *Synecocystis* trHb, the predicted value is 2,0⋅10^7^ *M* ^−1^ *s* ^−1^ (Fig 4) while experimental one is 2,4⋅10^8^ *M* ^−1^ *s* ^−1^, which is an approximate 10-fold overestimation.

### Estimation of the ligand dissociation rates

As in the case of the small ligand association process, the dissociation also involves two main processes, breaking the ligand stabilization network and further ligand migration from protein heme cavity to the bulk solvent, however only the former contributes significantly to the dissociation and is used in the model (Bustamante et. al. [34]). Previously, we showed that QM/MM computed oxygen dissociation energy provides a good estimate of the thermal barrier for oxygen release (and thus *k*_*off*_) [5,70,81]. Note that the oxygen release process is a unimolecular reaction, so the proposed model is:

$$k_{off}calc=e^{\frac{-\Delta E_{O_{2}}}{RT}}$$ (5)

This kind of approach was successfully previously used to study NO dissociation from porphyrins [88,89]. However, if $\Delta E_{O_{2}}$ values are used directly in eq 5, *k*_*off*_ calculated values are significantly underestimated and thus further corrections need to be performed. First, it is well known that computed oxygen dissociation energies from the heme are significantly overestimated due to the fact that a low (singlet) to high spin (quintet) spin transition is involved and DFT overestimates the energy of the spin gap, favoring low spin configurations [90]. Second, $\Delta E_{O_{2}}$ values are computed for the optimized, i.e. best possible conformation at 0K, and kinetic values are computed at room temperature. Last but not least, due to errors intrinsic to DFT-based QM/MM methods, the computed energies are strongly dependent on the exchange-correlation functional and basis set. This can be partially considered and corrected by estimating the oxygen binding energy relative to that of a free heme, using eq 6.

$$k_{off}calc=e^{\frac{-\Delta \Delta E_{O_{2}}}{RT}}$$ (6)

where $\Delta \Delta E_{O_{2}}$ corresponds to the $\Delta E_{O_{2}}$ (oxygen binding energy computed as described above) and the difference between *ΔE*_*heme*_, the calculated oxygen binding energy of an isolated imidazol bound heme in vacuum (which is 22*Kcalmol* ^−1^) and $k_{off}{}_{{}_{freeheme}}$ value (10^4^ s^−1^) [43,70]. The computed *k*_*off*_ values for all possible combinations of active site residues are presented in S2 Table.

## Supporting Information

Tables containing all computed rates for all possible trHbs. Bayesian trees for the four main trHb groups and additional graphs for the analysis of rate constants are also available.

S1 Fig Bayesian phylogenetic tree presented in Fig 2A, main text, with each leaf labeled with its corresponding UniProt ID. (TIF)

S2 Fig Bayesian phylogenetic tree built for N group of trHbs using MrBayes 3, with each leaf labeled with its corresponding UniProt ID. Tree is reconstructed with independently BMGE-trimmed subMSA consisting of N clade's sequences and shown as radial phylograms. The scale bar represents a distance of 0.1 accepted amino acid substitutions per site.
(EPS)

S3 Fig Bayesian phylogenetic tree built for O group of trHbs using MrBayes 3, with each leaf labeled with its corresponding UniProt ID. Tree is reconstructed with independently BMGE-trimmed subMSA consisting of O clade's sequences and shown as radial phylograms. The scale bar represents a distance of 0.1 accepted amino acid substitutions per site.
(EPS)

S4 Fig Bayesian phylogenetic tree built for P and Q groups of trHbs using MrBayes 3, with each leaf labeled with its corresponding UniProt ID. Trees are reconstructed with independently BMGE-trimmed subMSAs consisting of P and Q clade's sequences and shown as radial phylograms. The scale bar represents a distance of 0.1 accepted amino acid substitutions per site.
(EPS)

S5 Fig Plot of computed *log*(*k*_*off*_ *calc*) vs *log*(*k*_*on*_ *calc*) values. The larger the circle's size, the greater the number of proteins with the same computed values.
(TIF)

S6 Fig Histogram of p50 values for trHbs cases with all their available physicochemical computed values. (TIF)

S7 Fig Phylogenetic trees for each group of trHbs are shown as circular phylograms with mapped *k*_*off*_ and *k*_*on*_ in a logarithmic scale as concentric circles using heat maps. The phylograms show the topology derived from Fig 2A.
(TIF)

S8 Fig Maximum Likelihood tree built using phyML 3 with sequences taken from Vuletich and Lecomte’s work [16]. **T**he same phylogenetic topology with clustering of N, O and P (or I, II and III) groups is observed.
(TIF)

S1 Table Analysis of Specificity Determining Positions (SDP) and Mutual Information (MI) and cumulative MI with their corresponding structural positions. Shown are the data obtained with SDPfox and Mistic. SDPs were rated according to their MI with SDP E7.
(DOCX)

S2 TableActive site and tunnel residues combinations with their respective computed parameters to define the kinetic constants (*k*_*on*_ *calc*) and (*k*_*off*_ *calc*) for all the trHbs of each organisms.In order to obtain (*k*_*on*_ *calc*), the following equation should to be used: $k_{on}calc=\frac{k_{B}T}{h}\cdot k_{tunnels}\cdot K_{H_{2}O}$ (explained in detail in Methods section).(DOCX)

S3 TableProteins plotted at Fig 4, with their corresponding values for *log*(*k*_*on*_), *log*(*k*_*on*_ *calc*), $K_{H_{2}O}$ and the active site residues.(DOCX)

S4 Table Proteins plotted at Fig 5, with their corresponding values for *log*(*k*_*off*_), *log*(*k*_*off*_ *calc*) and the active site residues. (DOCX)

S1 Script *Ad-hoc* TCL program to perform an analysis of the ILS results. Step 1 of 3.
(TCL)

S2 Script *Ad-hoc* TCL program to perform an analysis of the ILS results. Step 2 of 3.
(TCL)

S3 Script *Ad-hoc* TCL program to perform an analysis of the ILS results. Step 3 of 3.
(TCL)
