# Supplementary figures and images for: Evolutionary and Functional Relationships in the Truncated Hemoglobin Family
Source: PLoS Comput Biol. 2016 Jan 20;12(1):e1004701. doi: 10.1371/journal.pcbi.1004701 (PMC4720485; doi:10.1371/journal.pcbi.1004701)

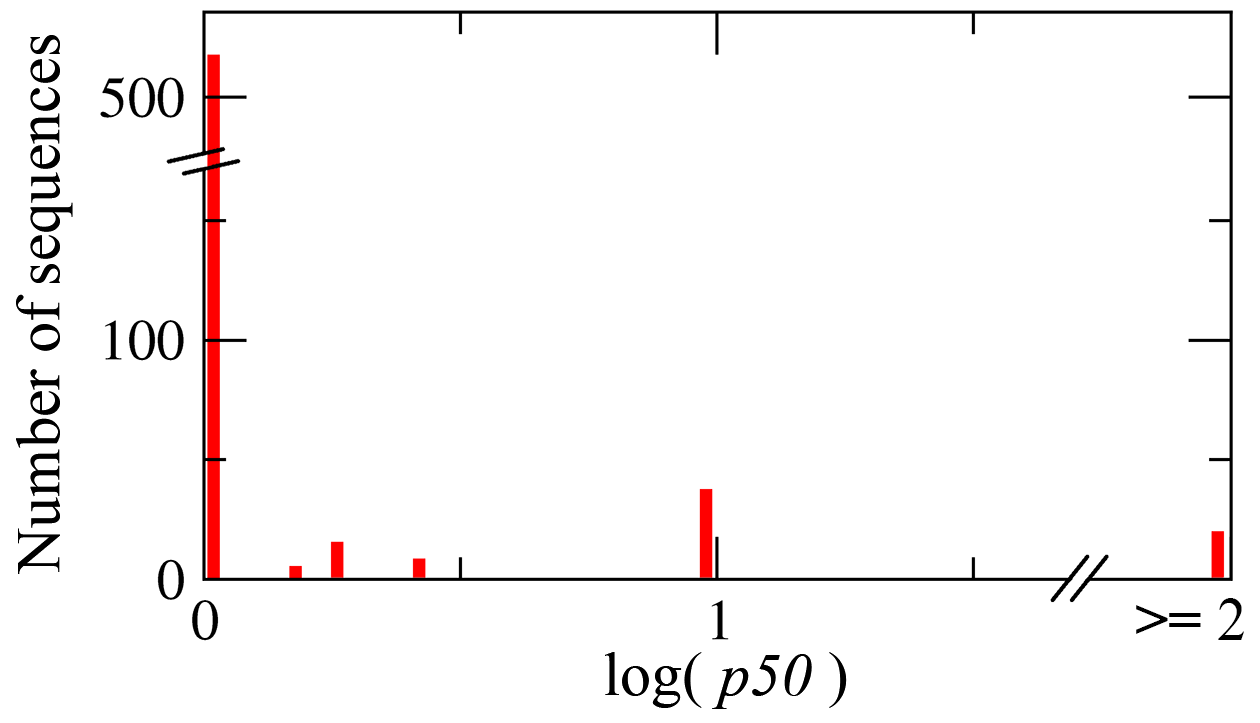

Supplement: S5 Fig — The larger the circle's size, the greater the number of proteins with the same computed values. (TIF) [file pcbi.1004701.s005.tif]

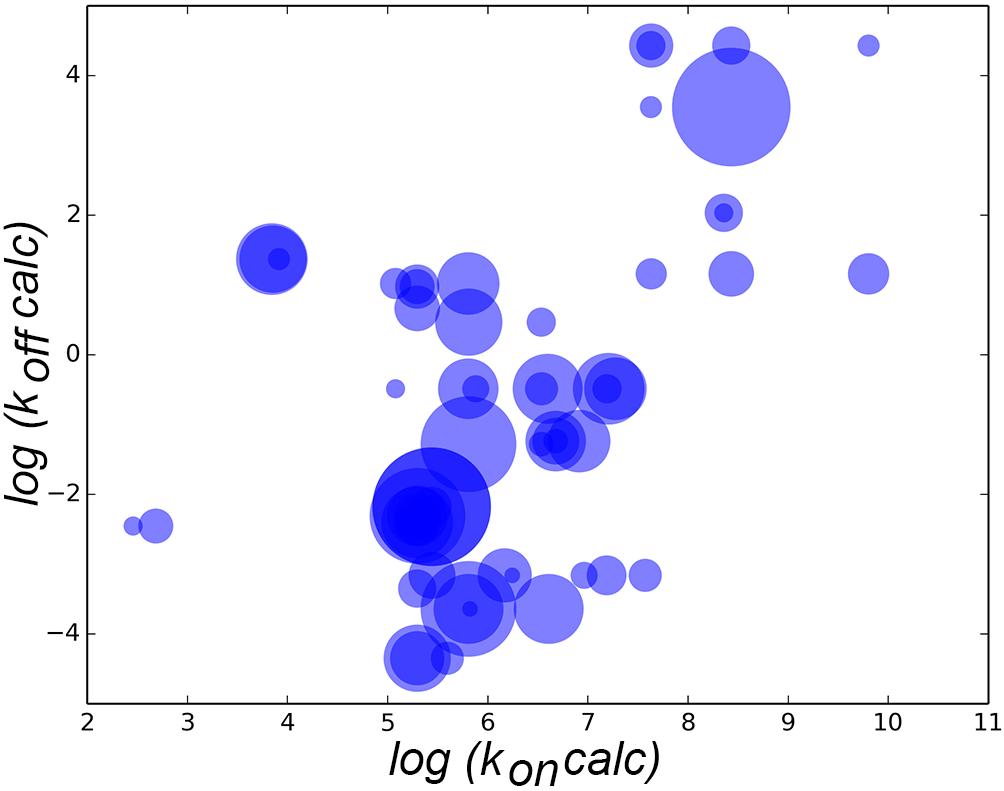

Supplement: S6 Fig — (TIF) [file pcbi.1004701.s006.tif]

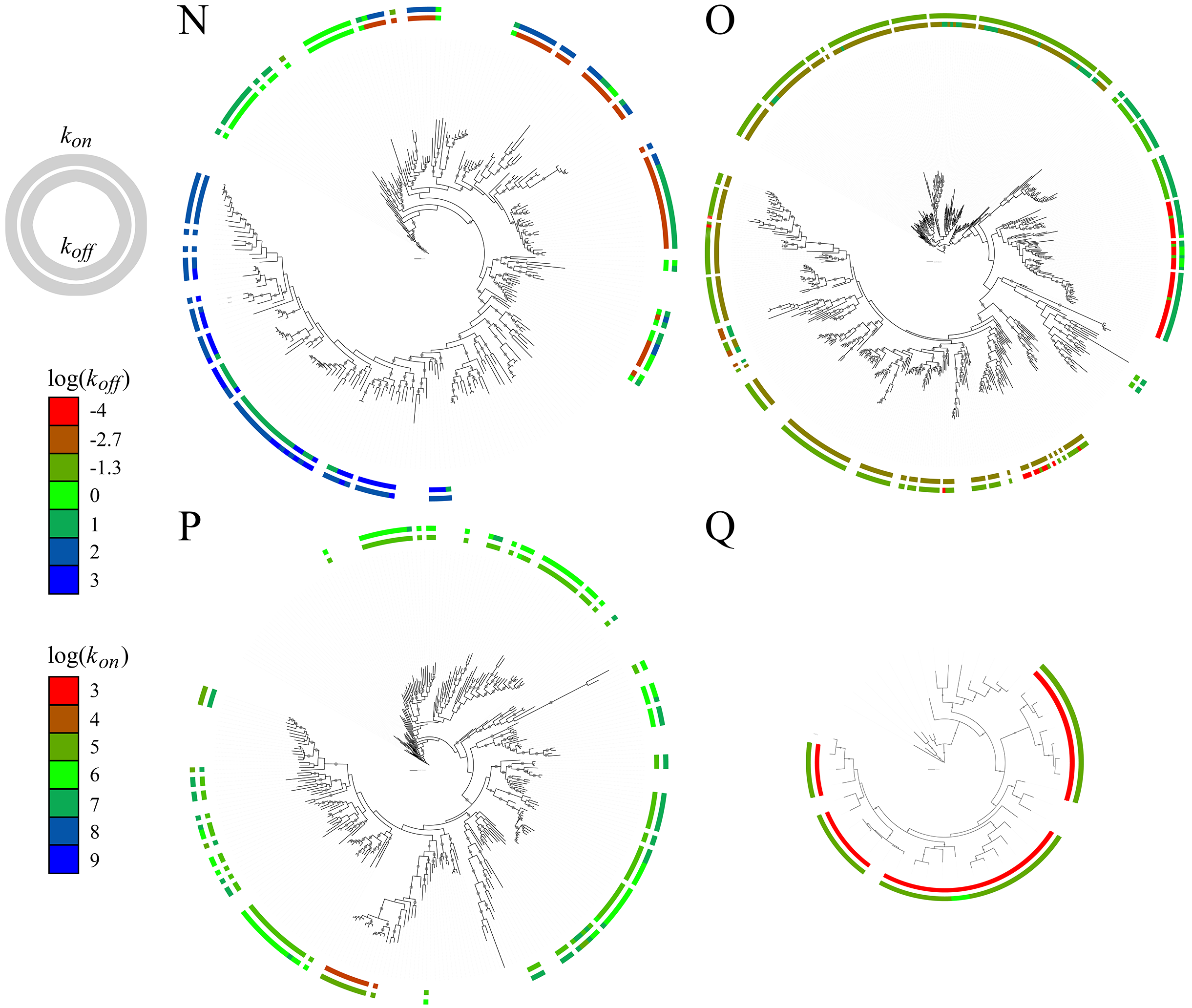

Supplement: S7 Fig — The phylograms show the topology derived from Fig 2A. (TIF) [file pcbi.1004701.s007.tif]

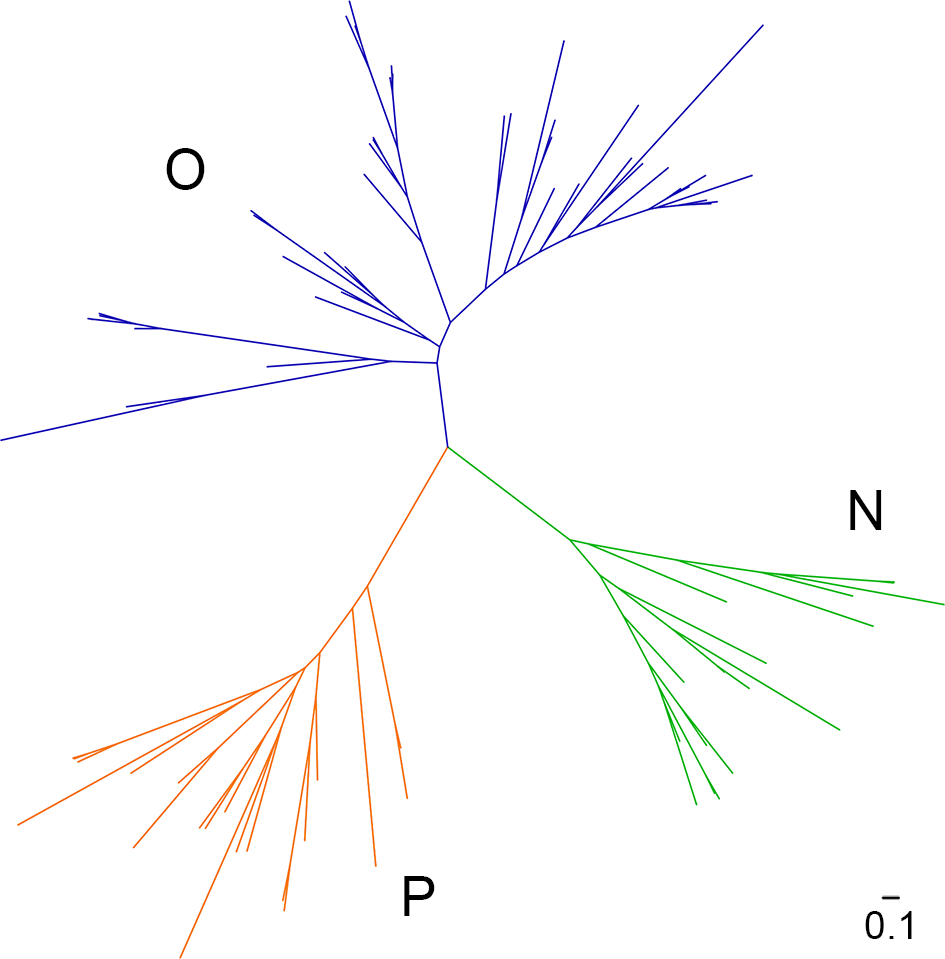

Supplement: S8 Fig — The same phylogenetic topology with clustering of N, O and P (or I, II and III) groups is observed. (TIF) [file pcbi.1004701.s008.tif]
