# Supplementary material for: Evolutionary and Functional Relationships in the Truncated Hemoglobin Family
Source: PLoS Comput Biol. 2016 Jan 20;12(1):e1004701. doi: 10.1371/journal.pcbi.1004701 (PMC4720485; doi:10.1371/journal.pcbi.1004701)
Supplement: S1 Table — Shown are the data obtained with SDPfox and Mistic. SDPs were rated according to their MI with SDP E7. (DOCX) [file pcbi.1004701.s009.docx]

| **Combined Rate** | **SDPfox** | | **Mistic** | | **Structural Position** |
| --- | --- | --- | --- | --- | --- |
|  | **Z-score** | **P-value** | **MI with E7** | **cMI** |  |
| 1 | 86,90 | -3775,22 | NA | 223,82 | E7 |
| 2 | 56,57 | -6400,31 | 19,67 | 208,72 | E15 |
| 3 | 81,76 | -6683,97 | 15,34 | 214,79 | G12 |
| 4 | 33,81 | -8009,17 | 15,24 | 119,32 | H9 |
| 5 | 35,42 | -6903,53 | 14,59 | 189,96 | G8 |
| 6 | 29,97 | -8995,46 | 14,10 | 96,49 | G11 |
| 7 | 59,80 | -5363,92 | 12,77 | 164,15 | F4 |
| 8 | 41,87 | -5257,86 | 11,84 | 154,70 | E4 |
| 9 | 30,50 | -8845,49 | 10,71 | 64,19 | C6 |
| 10 | 51,02 | -6506,17 | 10,56 | 124,83 | G5 |
| 11 | 33,07 | -8208,31 | 8,80 | 225,91 | E20 |
| 12 | 38,29 | -5865,07 | 8,66 | 130,37 | E18 |
| 13 | 34,63 | -7200,55 | 7,68 | 127,17 | EF8 |
| 14 | 36,63 | -6039,21 | 7,45 | 61,20 | F7 |
| 15 | 30,64 | -8460,71 | 7,42 | 72,42 | F5 |
| 16 | 31,74 | -8066,83 | 6,55 | 85,80 | H13 |
|  | 31,61 | -8502,18 | 5,53 | 77,03 | H16 |
|  | 35,94 | -6459,36 | 5,19 | 63,01 | FG1 |
|  | 33,85 | -7452,90 | 4,46 | 64,40 | H12 |
|  | 40,95 | -5868,73 | 4,04 | 36,43 | E17 |
