# Supplementary material for: Evolutionary and Functional Relationships in the Truncated Hemoglobin Family
Source: PLoS Comput Biol. 2016 Jan 20;12(1):e1004701. doi: 10.1371/journal.pcbi.1004701 (PMC4720485; doi:10.1371/journal.pcbi.1004701)
Supplement: S2 Table — In order to obtain (k on calc), the following equation should to be used: koncalc=kBTh⋅ktunnels⋅KH2O (explained in detail in Methods section). (DOCX) [file pcbi.1004701.s010.docx]

| Active site residues B10-CD1-E7-E11-G8 | $\boldsymbol{k}_{\boldsymbol{off}}\boldsymbol{calc}$  $\boldsymbol{[}\boldsymbol{s}^{\boldsymbol{-1}}\boldsymbol{]}$ | $\boldsymbol{K}_{\boldsymbol{H}_{\boldsymbol{2}}\boldsymbol{O}}$ | Specie (uniprotID) |
| --- | --- | --- | --- |
| YHTLW | 3,52E-03 | 3,04E-05 | O: Polaromonas naphthalenivorans CJ2(A1VPS5_POLNA), Marinomonas sp MWYL1(A6VRD1_MARMS), Xanthobacter autotrophicus Py2(A7IBJ8_XANP2), mine drainage metagenome(E6PT94_9ZZZZ), Methylosinus trichosporium OB3b(D5QKG5_METTR), Nitrospirillum amazonense Y2(G1XWD4_9PROT), Thiomonas intermedia K12(D5X1M5_THIK1), Burkholderiales bacterium JOSHI_001(H5WW47_9BURK), Azorhizobium caulinodans ORS 571(A8IIN8_AZOC5), Beggiatoa sp PS(A7BTI1_9GAMM), marine gamma proteobacterium HTCC2143(A0YE49_9GAMM), Burkholderia xenovorans LB400(Q13N39_BURXL), Methylocella silvestris BL2(B8EJ43_METSB,B8ELT2_METSB) |
| YFMKW | 4,47E-04 | 3,04E-05 | P: Rhizobium leguminosarum bv trifolii(B5ZXR3_RHILW), Agrobacterium sp ATCC 31749(F5J5A9_9RHIZ), Agrobacterium tumefaciens F2(F7U441_RHIRD), Sinorhizobium medicae WSM419(A6ULS3_SINMW), Agrobacterium tumefaciens CCNWGS0286(G6XXM2_RHIRD), Sinorhizobium fredii NGR234(C3KMT5_RHISN), Rhizobium sp PDO1-076(H4F0B9_9RHIZ), Rhizobium etli CFN 42(Q2KDK7_RHIEC), Agrobacterium tumefaciens 5A(H0HAA1_RHIRD), Rhizobium etli CIAT 652(B3PYD4_RHIE6), Rhizobium leguminosarum bv viciae(Q1MMQ0_RHIL3), Sinorhizobium meliloti CCNWSX0020(H0FT84_RHIML) |
| YFQVW | 1,05E+01 | 9,85E-05 | P: Lysobacter sp ATCC 53042(F8TUD7_9GAMM), Roseobacter sp AzwK-3b(A6FK96_9RHOB) |
| VFLLL | 2,69E+04 | 1,87E-01 | N: Acidithiobacillus caldus SM-1(F9ZLS8_ACICS,F9ZPY0_ACICS), Limnobacter sp MED105(A6GNQ3_9BURK), Halothiobacillus neapolitanus c2(D0L0N5_HALNC), Hyphomonas neptunium ATCC 15444(Q0BZ22_HYPNA), Oxalobacteraceae bacterium IMCC9480(F1VZN5_9BURK), Acidiphilium cryptum JF-5(A5G112_ACICJ), Burkholderiales bacterium JOSHI_001(H5WPM9_9BURK), Brevundimonas subvibrioides ATCC 15264(D9QNS8_BRESC), Cupriavidus necator N-1(F8GPA5_CUPNN), Ramlibacter tataouinensis TTB310(F5Y5W2_RAMTT), Methylibium petroleiphilum PM1(A2SF55_METPP), Curvibacter putative symbiont of(C9Y9G0_9BURK), Candidatus Nitrospira defluvii(D8PAF5_9BACT,D8P7L8_9BACT), Acidovorax sp NO-1(H0C3W6_9BURK) |
| YHMLW | 2,35E+01 | 3,04E-05 | O: Novosphingobium aromaticivorans DSM 12444(Q2GCA5_NOVAD), Novosphingobium pentaromativorans US6-1(G6EBK2_9SPHN), Sphingobium sp SYK-6(G2ISN4_9SPHN), Caulobacter segnis ATCC 21756(D5VQ98_CAUST), Novosphingobium sp PP1Y(F6IFD3_9SPHN), Caulobacter sp K31(B0T7B3_CAUSK), Novosphingobium nitrogenifigens DSM 19370(F1Z5X0_9SPHN), Sphingobium chlorophenolicum L-1(F6ESW7_SPHCR), Sphingomonas wittichii RW1(A5V5K0_SPHWW), marine gamma proteobacterium HTCC2148(B7RWE6_9GAMM) |
| YHSFW | 3,70E-03 | 3,04E-05 | O: Rhizobium leguminosarum bv trifolii(B5ZZM9_RHILW), Agrobacterium sp ATCC 31749(F5J978_9RHIZ), Agrobacterium tumefaciens F2(F7U5G1_RHIRD), Sinorhizobium medicae WSM419(A6UBY2_SINMW), Limnobacter sp MED105(A6GP10_9BURK), Agrobacterium vitis S4(B9JR55_AGRVS), Asticcacaulis excentricus CB 48(E8RL60_ASTEC), Sinorhizobium fredii NGR234(C3MGD5_RHISN), Rhizobium sp PDO1-076(H4FB18_9RHIZ), Rhizobium etli CFN 42(Q2K563_RHIEC), Collimonas fungivorans Ter331(G0ACD4_COLFT), Agrobacterium tumefaciens 5A(H0H881_RHIRD), Rhizobium etli CIAT 652(B3PX70_RHIE6), Rhizobium leguminosarum bv viciae(Q1MD01_RHIL3), Agrobacterium radiobacter K84(B9J857_AGRRK), Burkholderia xenovorans LB400(Q142K1_BURXL), Sinorhizobium meliloti CCNWSX0020(H0GA12_RHIML) |
| YFLYW | 5,25E+01 | 3,04E-05 | P: Flavobacteriaceae bacterium 3519-10(C6WZG4_FLAB3), Gramella forsetii KT0803(A0M173_GRAFK), Muricauda ruestringensis DSM 13258(G2PIT9_MURRD), Bdellovibrio bacteriovorus HD100(Q6MK94_BDEBA), Pedobacter heparinus DSM 2366(C6XVI0_PEDHD), Haliscomenobacter hydrossis DSM 1100(F4L2U7_HALH1), Spirosoma linguale DSM 74(D2QM53_SPILD), Niabella soli DSM 19437(H1NKD8_9SPHI,H1NM30_9SPHI), Elizabethkingia anophelis Ag1(H0KQP6_9FLAO), Riemerella anatipestifer RA-GD(F0TNT5_RIEAR), Flavobacterium johnsoniae UW101(A5FIM9_FLAJ1,A5FCK0_FLAJ1), Pedobacter saltans DSM 12145(F0SCW7_PEDSD), Fluviicola taffensis DSM 16823(F2I9I0_FLUTR), Meiothermus ruber DSM 1279(D3PTQ1_MEIRD), Chthoniobacter flavus Ellin428(B4D1F1_9BACT), Sphingobacterium spiritivorum ATCC 33861(D7VPR8_9SPHI), Leadbetterella byssophila DSM 17132(E4RVD1_LEAB4), Runella slithyformis DSM 19594(F8EAH9_RUNSL), Owenweeksia hongkongensis DSM 17368(G8R6F6_OWEHD), Myroides odoratimimus CIP 101113(H1H5B0_9FLAO), Marivirga tractuosa DSM 4126(E4TR24_MARTH), Dysgonomonas mossii DSM 22836(F8X358_9PORP), Flavobacterium frigoris PS1(H7FR52_9FLAO), unidentified eubacterium SCB49(A6ENP0_9BACT), Ktedonobacter racemifer DSM 44963(D6TP19_9CHLR), Zobellia galactanivorans(G0L0F4_ZOBGA), Chryseobacterium gleum ATCC 35910(D7VV61_9FLAO), Nitrobacter winogradskyi Nb-255(Q3SUN3_NITWN), Niastella koreensis GR20-10(G8TDR8_NIAKG), Nitrobacter hamburgensis X14(Q1QHS5_NITHX), Flavobacterium columnare ATCC 49512(G8X694_FLACA), Myroides odoratus DSM 2801(H1ZC40_9FLAO), Nitrobacter sp Nb-311A(A3WUX8_9BRAD), Weeksella virosa DSM 16922(F0P268_WEEVC), Cyclobacterium marinum DSM 745(G0J1W9_CYCMS), Gallibacterium anatis UMN179(F4H9I5_GALAU), Solitalea canadensis DSM 3403(H8KRX3_FLECA), Pedobacter sp BAL39(A6EG59_9SPHI) |
| LFFFV | 1,08E+02 | 1,87E-01 | N: Halomonas sp TD01(F7SSB5_9GAMM), Congregibacter litoralis KT71(A4ACE9_9GAMM) |
| YFKVW | 2,93E+00 | 5,27E-04 | P: mine drainage metagenome(E6Q9Q5_9ZZZZ), Leadbetterella byssophila DSM 17132(E4RWM0_LEAB4) |
| YYALW | 6,65E-03 | 3,04E-05 | O: Mycobacterium tuberculosis NCGM2209(G2UUQ6_MYCTU), Clavibacter michiganensis subsp sepedonicus(B0RAR2_CLAMS), Micrococcus luteus SK58(D3LL05_MICLU), Corynebacterium efficiens YS-314(Q8FN04_COREF), Streptomyces sviceus ATCC 29083(D6XBM2_9ACTO), Microbacterium laevaniformans OR221(H8E4L0_9MICO), Mycobacterium rhodesiae NBB3(G8RIA5_MYCRN), Leifsonia xyli subsp xyli(Q6AFT0_LEIXX), Tsukamurella paurometabola DSM 20162(D5UWZ7_TSUPD), Actinoplanes sp SE50110(G8S1V0_ACTS5), Mycobacterium intracellulare MOTT-64(H8JRE9_MYCIT), Cellulomonas flavigena DSM 20109(D5UCE3_CELFN), Corynebacterium glutamicum ATCC 14067(G6WV80_CORGT), Streptomyces venezuelae ATCC 10712(F2R2J2_STRVP), Blastococcus saxobsidens DD2(H6RIR9_BLASD), Xylanimonas cellulosilytica DSM 15894(D1BZH5_XYLCX), Intrasporangium calvum DSM 43043(E6S6Y0_INTC7), Nocardioidaceae bacterium Broad-1(E9V271_9ACTO), Gordonia effusa NBRC 100432(H0QZ84_9ACTO), Janibacter sp HTCC2649(A3TFS8_9MICO), Streptomyces zinciresistens K42(G2GKZ3_9ACTO), Beutenbergia cavernae DSM 12333(C5BXJ9_BEUC1), Frankia alni ACN14a(Q0RPN6_FRAAA), Segniliparus rotundus DSM 44985(D6Z7C0_SEGRD), Mycobacterium tusciae JS617(H1JSN5_9MYCO), Corynebacterium amycolatum SK46(E2MXD8_9CORY), Corynebacterium variabile DSM 44702(G0HB35_CORVD), Corynebacterium accolens ATCC 49725(C0WJJ8_9CORY), Arthrobacter arilaitensis Re117(E1VWS4_ARTAR), Saccharomonospora marina XMU15(H5X3G1_9PSEU), Mycobacterium parascrofulaceum ATCC BAA-614(D5PH46_9MYCO), Mycobacterium avium 104(A0QDE2_MYCA1), Streptomyces ghanaensis ATCC 14672(D6A297_9ACTO), Mycobacterium vanbaalenii PYR-1(A1TCE3_MYCVP), Nocardia farcinica IFM 10152(Q5Z082_NOCFA), Corynebacterium diphtheriae NCTC 13129(Q6NFT6_CORDI), Saccharomonospora cyanea NA-134(H5XE69_9PSEU), Brachybacterium faecium DSM 4810(C7MBP3_BRAFD), Mycobacterium colombiense CECT 3035(F9QGJ2_9MYCO), Corynebacterium lipophiloflavum DSM 44291(C0XRD8_9CORY), Corynebacterium resistens DSM 45100(F8DZ36_CORRG), Frankia sp CN3(G6HKE2_9ACTO), Streptomyces griseoaurantiacus M045(F3NCP4_9ACTO), Mycobacterium abscessus subsp bolletii(H0IML9_MYCAB), Nakamurella multipartita DSM 44233(C8XGN0_NAKMY), marine actinobacterium PHSC20C1(A4AGR0_9ACTN), Isoptericola variabilis 225(F6FQG9_ISOV2), Jonesia denitrificans DSM 20603(C7R2X0_JONDD), Frankia sp EAN1pec(A8L215_FRASN), Thermobifida fusca(2BMM), Corynebacterium ammoniagenes DSM 20306(D5NXP9_CORAM), Pseudomonas stutzeri A1501(A4VJW3_PSEU5), Dietzia cinnamea P4(E6J538_9ACTO), Algoriphagus machipongonensis(A3I308_9BACT), Corynebacterium casei UCMA 3821(G7HW23_9CORY), Mycobacterium gilvum PYR-GCK(A4T2Q7_MYCGI), Streptomyces cattleya NRRL 8057(F8JS37_STREN), Gordonia araii NBRC 100433(G7H143_9ACTO), Saccharomonospora paurometabolica YIM 90007(G4J0W0_9PSEU), Streptomyces griseoflavus Tu4000(D9Y0C3_9ACTO), Streptomyces viridochromogenes DSM 40736(D9X9K7_STRVR), Corynebacterium pseudogenitalium ATCC 33035(E2S653_9CORY), Micromonospora sp ATCC 39149(C4RPG9_9ACTO), Corynebacterium glutamicum ATCC 13032(Q8NMW6_CORGL), Stackebrandtia nassauensis DSM 44728(D3Q6N6_STANL), Saccharomonospora glauca K62(H1JG78_9PSEU), Frankia sp CcI3(Q2JDW4_FRASC), Mycobacterium rhodesiae JS60(G4I1H8_MYCRH), Streptomyces hygroscopicus subsp jinggangensis(H2K910_STRHJ), Thermobispora bispora DSM 43833(D6Y4S6_THEBD), Mycobacterium thermoresistibile ATCC 19527(G7CJC9_MYCTH), Gordonia sputi NBRC 100414(H5U4M1_9ACTO), Mycobacterium leprae(Q9X7B3_MYCLE), Gordonia otitidis NBRC 100426(H5TSE0_9ACTO), Nocardia brasiliensis ATCC 700358(H5RS95_9NOCA), Mycobacterium sp MCS(Q1B5W7_MYCSS), Kocuria rhizophila DC2201(B2GG22_KOCRD), Corynebacterium striatum ATCC 6940(C2CLE9_CORST), Corynebacterium jeikeium K411(Q4JWW0_CORJK), Corynebacterium glucuronolyticum ATCC 51866(C2GJU6_9CORY), Micromonospora aurantiaca ATCC 27029(D9TAK4_MICAI), Streptomyces violaceusniger Tu 4113(G2NX27_STRVO), Microbacterium testaceum StLB037(E8NDL4_MICTS), Streptomyces sp SPB78(D9UM81_9ACTO), Mycobacterium marinum M(B2HNH8_MYCMM), Frankia sp EuI1c(E3J5P0_FRASU), Saccharopolyspora erythraea NRRL 2338(A4F9C9_SACEN), Saccharomonospora viridis DSM 43017(C7MZW7_SACVD), Streptomyces himastatinicus ATCC 53653(D9WQC1_9ACTO), Gordonia alkanivorans NBRC 16433(F9VZA5_9ACTO), Corynebacterium aurimucosum ATCC 700975(C3PII6_CORA7), Salinispora tropica CNB-440(A4XAM0_SALTO), Mycobacterium smegmatis str MC2(A0R1B8_MYCS2), Pseudomonas stutzeri ATCC 14405(H7EU46_PSEST), Planctomyces maris DSM 8797(A6CB44_9PLAN), Streptomyces bingchenggensis BCW-1(D7C3F4_STRBB), Saccharomonospora azurea NA-128(H8GDG3_9PSEU), Mycobacterium sp JDM601(F5YY56_MYCSD), Streptomyces sp e14(D6K0W4_9ACTO), Frankia sp EUN1f(D3D1B2_9ACTO), Gordonia neofelifaecis NRRL B-59395(F1YI30_9ACTO), Kytococcus sedentarius DSM 20547(C7NFL3_KYTSD), Gordonia bronchialis DSM 43247(D0LAH0_GORB4), Corynebacterium ulcerans 809(G0CMU9_CORUL), Salinispora arenicola CNS-205(A8M1P3_SALAI), Cellulomonas fimi ATCC 484(F4H5S7_CELFA), Nocardiopsis dassonvillei subsp dassonvillei(D7B3R3_NOCDD), Gordonia amarae NBRC 15530(G7GMX0_9ACTO), Catenulispora acidiphila DSM 44928(C7Q9L1_CATAD), Nocardia cyriacigeorgica GUH-2(H6R5I9_NOCCG), Gordonia polyisoprenivorans VH2(H6MX45_GORPV), Thermomonospora curvata DSM 43183(D1A9X9_THECD), Geodermatophilus obscurus DSM 43160(D2SCM4_GEOOG), Mobilicoccus pelagius NBRC 104925(H5UVC5_9MICO), Corynebacterium pseudotuberculosis 106-A(G7U1I8_CORPS), Planctomyces limnophilus DSM 3776(D5SYN7_PLAL2), Frankia symbiont of Datisca(D3M5P7_9ACTO), Corynebacterium genitalium ATCC 33030(D7WC24_9CORY), Brevibacterium mcbrellneri ATCC 49030(D4YQV3_9MICO), Streptomyces scabiei 8722(C9Z5X1_STRSW), Acidothermus cellulolyticus 11B(A0LVC1_ACIC1) |
| YFLTW | 1,06E-02 | 5,27E-04 | P: Kordia algicida OT-1(A9E7W3_9FLAO), Mesorhizobium alhagi CCNWXJ12-2(H0HZ33_9RHIZ), Gramella forsetii KT0803(A0M1A1_GRAFK), Streptomyces venezuelae ATCC 10712(F2R152_STRVP), Psychroflexus torquis ATCC 700755(Q1VP43_9FLAO), Acidobacterium capsulatum ATCC 51196(C1F2U7_ACIC5), Mesorhizobium amorphae CCNWGS0123(G6YLR0_9RHIZ), Mesorhizobium australicum WSM2073(G4K804_9RHIZ), Capnocytophaga canimorsus Cc5(F9YVW9_CAPCC), Sulfurimonas denitrificans DSM 1251(Q30PW1_SULDN), Kitasatospora setae KM-6054(E4N1M3_KITSK), Flavobacterium johnsoniae UW101(A5FJK5_FLAJ1), Citreicella sp SE45(D0DE45_9RHOB), Pelagibacterium halotolerans B2(G4RBW3_PELHB), Gillisia limnaea DSM 15749(H2BYC9_9FLAO), Phenylobacterium zucineum HLK1(B4RHN0_PHEZH), Bizionia argentinensis JUB59(G2ECI5_9FLAO), Zobellia galactanivorans(G0LBS1_ZOBGA), Rhodopseudomonas palustris CGA009(Q6N698_RHOPA), Cellulophaga lytica DSM 7489(F0RE10_CELLC), Flavobacteria bacterium BBFL7(Q26HY6_FLABB), Alkalilimnicola ehrlichii MLHE-1(Q0A5B2_ALHEH), Acetobacter pasteurianus IFO 3283-12(C7L997_ACEPA), Sphingomonas wittichii RW1(A5VB57_SPHWW), Sulfurimonas gotlandica GD1(B6BLP9_9PROT), Burkholderia sp YI23(G8M460_9BURK), Cyclobacterium marinum DSM 745(G0IZJ2_CYCMS), Flavobacteriales bacterium ALC-1(A8ULC5_9FLAO), Cellulophaga algicola DSM 14237(E6XF16_CELAD,E6XCC0_CELAD) |
| YHALW | 4,92E-03 | 3,04E-05 | O: Methylotenera versatilis 301(D7DIW7_METS0), Comamonas testosteroni ATCC 11996(H1RK32_COMTE), Bordetella petrii DSM 12804(A9IJT5_BORPD), Methylobacter tundripaludum SV96(G3IT70_9GAMM), Variovorax paradoxus EPS(E6UXE4_VARPE), Alicycliphilus denitrificans K601(F4GAB4_ALIDK), Variovorax paradoxus S110(C5CWP3_VARPS), Methylomonas methanica MC09(G0A2B2_METMM), Methylomicrobium alcaliphilum 20Z(G4T327_META2), Polaromonas sp JS666(Q12EZ1_POLSJ), Novosphingobium aromaticivorans DSM 12444(Q2G523_NOVAD), Acidovorax avenae subsp avenae(F0Q0V5_ACIAP), Marinomonas mediterranea MMB-1(F2JVC2_MARM1), Neisseria wadsworthii 9715(G4CMK5_9NEIS), Polaromonas naphthalenivorans CJ2(A1VKI6_POLNA), Rhodoferax ferrireducens T118(Q21TE0_RHOFD), Methylovorus sp MP688(E4QIY5_METS6), Methylobacillus flagellatus KT(Q1H1W3_METFK), Methylococcus capsulatus str Bath(Q606Y3_METCA), Gallionella capsiferriformans ES-2(D9SK81_GALCS), Achromobacter arsenitoxydans SY8(H0F9F5_9BURK), Achromobacter xylosoxidans C54(E5U1W4_ALCXX), Beijerinckia indica subsp indica(B2IE39_BEII9), Azotobacter vinelandii DJ(C1DDP7_AZOVD), Achromobacter xylosoxidans A8(E3HFM9_ACHXA), Acidovorax ebreus TPSY(B9MEH6_ACIET), Bradyrhizobium sp STM 3843(H0TRL7_9BRAD), Sphingomonas sp SKA58(Q1NF48_9SPHN), Bordetella avium 197N(Q2KYN5_BORA1), Novosphingobium nitrogenifigens DSM 19370(F1ZB73_9SPHN), Mariprofundus ferrooxydans PV-1(Q0F2H6_9PROT), Achromobacter piechaudii ATCC 43553(D4XAA8_9BURK), Azoarcus sp BH72(A1K669_AZOSB), Acidovorax delafieldii 2AN(C5TD07_ACIDE), Leptothrix cholodnii SP-6(B1Y0J1_LEPCP), Azoarcus sp KH32C(H0PVQ4_9RHOO), Burkholderia vietnamiensis G4(A4JRN5_BURVG), Methylomicrobium album BG8(H8GH79_METAL), Ramlibacter tataouinensis TTB310(F5XW43_RAMTT), Methylotenera mobilis JLW8(C6WW87_METML), Sphingobium chlorophenolicum L-1(F6F2Z0_SPHCR), Herbaspirillum seropedicae SmR1(D8IZK7_HERSS), Curvibacter putative symbiont of(C9Y8P3_9BURK), Sphingobium japonicum UT26S(D4Z631_SPHJU), Bacteriovorax marinus SJ(E1WZL7_BACMS), Achromobacter xylosoxidans AXX-A(F7T4L5_ALCXX), Acidovorax sp NO-1(H0BTR3_9BURK), Bordetella pertussis Tohama I(Q7VWB0_BORPE), Methylomonas sp 16a(A3QVH5_9GAMM), Delftia acidovorans SPH-1(A9BT93_DELAS), Hylemonella gracilis ATCC 19624(F3KVD3_9BURK) |
| YFQTV | 2,06E+00 | 9,85E-05 | N: Paramecium multimicronucleatum(Q27213_9CILI), Paramecium triaurelia(Q7JQC9_9CILI), Paramecium tetraurelia(Q3SEA1_PARTE), Paramecium caudatum(TRHBN_PARCA) |
| YYAFW | 9,49E+00 | 3,04E-05 | O: Arthrobacter aurescens TC1(A1R7B2_ARTAT), Streptomyces sp AA4(D9VAR2_9ACTO), Arthrobacter sp FB24(A0JXM3_ARTS2), Streptomyces clavuligerus ATCC 27064(B5GVB3_STRCL), Arthrobacter chlorophenolicus A6(B8HA44_ARTCA), Kribbella flavida DSM 17836(D2PTJ8_KRIFD), Streptomyces sp Mg1(B4V9I1_9ACTO), Dermacoccus sp Ellin185(E3BBA3_9MICO), Streptomyces albus J1074(D6B196_9ACTO), Kitasatospora setae KM-6054(E4NIA7_KITSK), Streptomyces sp SirexAA-E(G2NKL6_9ACTO), Streptomyces griseus XylebKG-1(G0PQ09_STRGR), Streptosporangium roseum DSM 43021(D2BDZ5_STRRD), Amycolicicoccus subflavus DQS3-9A1(F6EQ94_AMYSD), Streptomyces avermitilis MA-4680 =(Q82CH9_STRAW), Actinosynnema mirum DSM 43827(C6WQ34_ACTMD), Arthrobacter phenanthrenivorans Sphe3(F0M4E0_ARTPP), Streptomyces sp SPB74(B5G7N3_9ACTO), Microlunatus phosphovorus NM-1(F5XP72_MICPN), Aeromicrobium marinum DSM 15272(E2SBB9_9ACTO), Streptomyces pratensis ATCC 33331(E8W9Z7_STRFA), Nocardioides sp JS614(A1SGF5_NOCSJ), Pseudonocardia dioxanivorans CB1190(F4D1N5_PSEUX), Arthrobacter globiformis NBRC 12137(H0QPD5_ARTGO), Streptomyces coelicolor A3(2)(Q9L250_STRCO), Streptomyces sp C(D9VWG0_9ACTO), Amycolatopsis mediterranei S699(G0G0V5_AMYMD) |
| YFTQW | 2,29E-04 | 5,27E-04 | O: Bacillus pumilus SAFR-032(A8FC02_BACP2), Deinococcus radiodurans R1(Q9RVM5_DEIRA), Macrococcus caseolyticus JCSC5402(B9EAR3_MACCJ), Staphylococcus pettenkoferi VCU012(H0DI57_9STAP), Bacillus subtilis subsp subtilis(), Oceanobacillus iheyensis HTE831(Q8ERT2_OCEIH), Bacillus halodurans C-125(Q9K8Z8_BACHD), Staphylococcus hominis subsp hominis(E5CNM8_STAHO), Bacillus sp NRRL B-14911(Q2B8X7_9BACI), Deinococcus geothermalis DSM 11300(Q1IY16_DEIGD), Staphylococcus lugdunensis N920143(F8KNU5_STALN), Staphylococcus aureus subsp aureus(G7ZMA6_STAAU), Bacillus sp 2_A_57_CT2(E5WE36_9BACI), Bacillus clausii KSM-K16(Q5WF01_BACSK), Planococcus donghaensis MPA1U2(E7RF73_9BACL), Geobacillus stearothermophilus(2BKM), Staphylococcus pseudintermedius ED99(F0P8H6_STAPE), Staphylococcus carnosus subsp carnosus(B9DIR9_STACT), Bacillus atrophaeus 1942(E3DVK0_BACA1), Bacillus sp BT1B_CT2(E5W4T9_9BACI), Anoxybacillus flavithermus WK1(B7GLK9_ANOFW), Bacillus sp SG-1(A6CQW7_9BACI), Prevotella dentalis DSM 3688(H1M638_9BACT), Solibacillus silvestris StLB046(F2F888_SOLSS), Bacillus selenitireducens MLS10(D6XTC7_BACIE), Sporosarcina newyorkensis 2681(F9DPW8_9BACL), Caldalkalibacillus thermarum TA2A1(F5L510_9BACI), Deinococcus maricopensis DSM 21211(E8U893_DEIML), Geobacillus thermoglucosidasius C56-YS93(F8CZM8_BACTR), Bacillus megaterium QM B1551(D5E038_BACMQ), Staphylococcus caprae M23864:W1(C5QQU4_STAEP), Staphylococcus capitis VCU116(F9L8I2_STACP), Bacillus smithii 7_3_47FAA(G9QK17_9BACI), Bacillus cytotoxicus NVH 391-98(A7GM87_BACCN), Bacillus pseudofirmus OF4(D3FV17_BACPE), Staphylococcus epidermidis RP62A(Q5HQG7_STAEQ), Bacillus cereus Rock3-44(C2W574_BACCE), Deinococcus deserti VCD115(C1D0M8_DEIDV), Staphylococcus haemolyticus JCSC1435(Q4L511_STAHJ), Staphylococcus saprophyticus subsp saprophyticus(Q49WD2_STAS1), Geobacillus sp WCH70(C5D799_GEOSW), Bacillus thuringiensis serovar huazhongensis(C3GXW6_BACTU), Bacillus sp B14905(A3IFZ5_9BACI), Bacillus amyloliquefaciens subsp plantarum(H8XIT3_BACAM), Bacillus cellulosilyticus DSM 2522(E6TX59_BACCJ) |
| YFHVW | 4,49E-05 | 3,04E-05 | Q: Streptomyces clavuligerus ATCC 27064(B5GVJ7_STRCL), Streptomyces venezuelae ATCC 10712(F2RB89_STRVP), Nocardioidaceae bacterium Broad-1(E9UUY1_9ACTO), Yersinia ruckeri ATCC 29473(C4ULC2_YERRU), Kribbella flavida DSM 17836(D2PQN0_KRIFD), Photorhabdus asymbiotica subsp asymbiotica(B6VNR2_PHOAA), Streptomyces sp Mg1(B4V629_9ACTO), Mesorhizobium amorphae CCNWGS0123(G6Y9K6_9RHIZ), Spirosoma linguale DSM 74(D2QK90_SPILD), Campylobacter jejuni(A0A0E2UWT7_CAMJU), Mesorhizobium australicum WSM2073(G4K8I2_9RHIZ), Fluviicola taffensis DSM 16823(F2IK57_FLUTR), Frankia sp CN3(G6H1I3_9ACTO), Nocardia brasiliensis ATCC 700358(), Frankia sp EuI1c(E3J683_FRASU), Ktedonobacter racemifer DSM 44963(D6U4L4_9CHLR), Pseudoalteromonas sp SANK 73390(F8J3E6_9GAMM), Streptomyces himastatinicus ATCC 53653(D9WP19_9ACTO), Brevundimonas subvibrioides ATCC 15264(D9QFN8_BRESC), Niastella koreensis GR20-10(G8TQW4_NIAKG), Streptomyces bingchenggensis BCW-1(D7CHT6_STRBB), Mesorhizobium opportunistum WSM2075(F7YDW2_MESOW), Mesorhizobium ciceri biovar biserrulae(E8TLH5_MESCW), Frankia sp EUN1f(D3D2S0_9ACTO), Streptomyces sp C(D9VK26_9ACTO), Mesorhizobium loti MAFF303099(Q98N74_RHILO), Hylemonella gracilis ATCC 19624(F3KNL2_9BURK), Amycolatopsis mediterranei S699(G0G7F8_AMYMD), Xenorhabdus bovienii SS-2004(D3V2A9_XENBS), Solitalea canadensis DSM 3403(H8KRM2_FLECA) |
| YFQQV | 6,91E-04 | 2,28E-04 | N: Synechocystis sp PCC 6803(H0PMQ1_9SYNC), Methylobacter tundripaludum SV96(G3IQT3_9GAMM,G3IQS9_9GAMM), Sideroxydans lithotrophicus ES-1(D5CMC9_SIDLE,D5CR69_SIDLE), Methylomonas methanica MC09(G0A4H3_METMM,F9ZWQ0_METMM), Methylomicrobium alcaliphilum 20Z(G4SUM0_META2), Tetrahymena pyriformis (TRHBN_TETPY), Corallococcus coralloides DSM 2259(H8N066_MYXCO), Plesiocystis pacifica SIR-1(A6G520_9DELT), Chlamydomonas moewusii(TRHN2_CHLMO,TRHN1_CHLMO), Methylococcus capsulatus str Bath(Q604N2_METCA), Arthrospira sp PCC 8005(H1WKW8_9CYAN), Cyanothece sp PCC 8802(C7QR53_CYAP0), Batrachochytrium dendrobatidis JAM81(F4P5H7_BATDJ), Legionella pneumophila subsp pneumophila(Q5ZSI2_LEGPH), Myxococcus xanthus DK 1622(Q1CX39_MYXXD), mine drainage metagenome(E6QPM5_9ZZZZ,E6QSU8_9ZZZZ), Legionella longbeachae NSW150(D3HLV8_LEGLN), Ichthyophthirius multifiliis strain G5(G0QQ62_ICHMG), Bermanella marisrubri(Q1N6W8_9GAMM), Volvox carteri(D8THA8_VOLCA), Methylocystis sp ATCC 49242(E8L3Y3_9RHIZ), Acaryochloris marina MBIC11017(B0CBZ4_ACAM1), Myxococcus fulvus HW-1(F8C695_MYXFH), Methylomicrobium album BG8(H8GM14_METAL), Chlamydomonas eugametos(TRHN1_CHLMO), Chlamydomonas reinhardtii(A8JAR3_CHLRE,A8JAR4_CHLRE), Coraliomargarita akajimensis DSM 45221(D5EIY8_CORAD), Methylomonas sp 16a(A3QVH3_9GAMM) |
| YFLKW | 4,62E+00 | 3,04E-05 | P: Oceanicaulis sp HTCC2633(A3UC56_9RHOB), Pseudovibrio sp JE062(B6R8B7_9RHOB), Novosphingobium aromaticivorans DSM 12444(Q2G809_NOVAD), Caulobacter segnis ATCC 21756(D5VN99_CAUST), Hyphomonas neptunium ATCC 15444(Q0C302_HYPNA), gamma proteobacterium NOR5-3(B8KFJ6_9GAMM), Xanthobacter autotrophicus Py2(A7IJS8_XANP2), Polymorphum gilvum SL003B-26A1(F2J051_POLGS), Maricaulis maris MCS10(Q0ARP0_MARMM), Hirschia baltica ATCC 49814(C6XNL3_HIRBI), Novosphingobium sp PP1Y(F6IGG4_9SPHN), Brevundimonas sp BAL3(B4WCH2_9CAUL), Congregibacter litoralis KT71(A4A461_9GAMM), Sphingomonas wittichii RW1(A5VGI9_SPHWW), Afipia sp 1NLS2(D6V129_9BRAD), marine gamma proteobacterium HTCC2148(B7RT24_9GAMM) |
|  |  |  |  |
| E7G key residues B10-CD1-E7-E11 | $\boldsymbol{k}_{\boldsymbol{E}\boldsymbol{7}\boldsymbol{G}}$  **[**$\boldsymbol{M}^{\boldsymbol{-1}}\boldsymbol{s}^{\boldsymbol{-1}}$**]** | | **Specie (uniprotID)** |
| YFTQ | 1,22E-03 | | O: Bacillus pumilus SAFR-032(A8FC02_BACP2), Deinococcus radiodurans R1(Q9RVM5_DEIRA), Macrococcus caseolyticus JCSC5402(B9EAR3_MACCJ), Staphylococcus pettenkoferi VCU012(H0DI57_9STAP), Oceanobacillus iheyensis HTE831(Q8ERT2_OCEIH), Bacillus halodurans C-125(Q9K8Z8_BACHD), Staphylococcus hominis subsp hominis(E5CNM8_STAHO), Bacillus sp NRRL B-14911(Q2B8X7_9BACI), Deinococcus geothermalis DSM 11300(Q1IY16_DEIGD), Staphylococcus lugdunensis N920143(F8KNU5_STALN), Staphylococcus aureus subsp aureus(G7ZMA6_STAAU), Bacillus sp 2_A_57_CT2(E5WE36_9BACI), Bacillus clausii KSM-K16(Q5WF01_BACSK), Planococcus donghaensis MPA1U2(E7RF73_9BACL), Staphylococcus pseudintermedius ED99(F0P8H6_STAPE), Staphylococcus carnosus subsp carnosus(B9DIR9_STACT), Bacillus atrophaeus 1942(E3DVK0_BACA1), Bacillus sp BT1B_CT2(E5W4T9_9BACI), Anoxybacillus flavithermus WK1(B7GLK9_ANOFW), Bacillus sp SG-1(A6CQW7_9BACI), Prevotella dentalis DSM 3688(H1M638_9BACT), Solibacillus silvestris StLB046(F2F888_SOLSS), Bacillus selenitireducens MLS10(D6XTC7_BACIE), Sporosarcina newyorkensis 2681(F9DPW8_9BACL), Caldalkalibacillus thermarum TA2A1(F5L510_9BACI), Deinococcus maricopensis DSM 21211(E8U893_DEIML), Geobacillus thermoglucosidasius C56-YS93(F8CZM8_BACTR), Bacillus megaterium QM B1551(D5E038_BACMQ), Staphylococcus caprae M23864:W1(C5QQU4_STAEP), Staphylococcus capitis VCU116(F9L8I2_STACP), Bacillus smithii 7_3_47FAA(G9QK17_9BACI), Bacillus cytotoxicus NVH 391-98(A7GM87_BACCN), Bacillus pseudofirmus OF4(D3FV17_BACPE), Staphylococcus epidermidis RP62A(Q5HQG7_STAEQ), Bacillus cereus Rock3-44(C2W574_BACCE), Deinococcus deserti VCD115(C1D0M8_DEIDV), Staphylococcus haemolyticus JCSC1435(Q4L511_STAHJ), Staphylococcus saprophyticus subsp saprophyticus(Q49WD2_STAS1), Geobacillus sp WCH70(C5D799_GEOSW), Bacillus thuringiensis serovar huazhongensis(C3GXW6_BACTU), Bacillus sp B14905(A3IFZ5_9BACI), Bacillus amyloliquefaciens subsp plantarum(H8XIT3_BACAM), Bacillus cellulosilyticus DSM 2522(E6TX59_BACCJ) |
| YFLK | 1,18E-11 | | P: Oceanicaulis sp HTCC2633(A3UC56_9RHOB), Pseudovibrio sp JE062(B6R8B7_9RHOB), Novosphingobium aromaticivorans DSM 12444(Q2G809_NOVAD), Caulobacter segnis ATCC 21756(D5VN99_CAUST), Hyphomonas neptunium ATCC 15444(Q0C302_HYPNA), gamma proteobacterium NOR5-3(B8KFJ6_9GAMM), Xanthobacter autotrophicus Py2(A7IJS8_XANP2), Polymorphum gilvum SL003B-26A1(F2J051_POLGS), Maricaulis maris MCS10(Q0ARP0_MARMM), Hirschia baltica ATCC 49814(C6XNL3_HIRBI), Novosphingobium sp PP1Y(F6IGG4_9SPHN), Brevundimonas sp BAL3(B4WCH2_9CAUL), Congregibacter litoralis KT71(A4A461_9GAMM), Sphingomonas wittichii RW1(A5VGI9_SPHWW), Afipia sp 1NLS2(D6V129_9BRAD), marine gamma proteobacterium HTCC2148(B7RT24_9GAMM) |
| YFLT | 1,18E-11 | | P: Kordia algicida OT-1(A9E7W3_9FLAO), Mesorhizobium alhagi CCNWXJ12-2(H0HZ33_9RHIZ), Gramella forsetii KT0803(A0M1A1_GRAFK), Streptomyces venezuelae ATCC 10712(F2R152_STRVP), Psychroflexus torquis ATCC 700755(Q1VP43_9FLAO), Acidobacterium capsulatum ATCC 51196(C1F2U7_ACIC5), Mesorhizobium amorphae CCNWGS0123(G6YLR0_9RHIZ), Mesorhizobium australicum WSM2073(G4K804_9RHIZ), Capnocytophaga canimorsus Cc5(F9YVW9_CAPCC), Sulfurimonas denitrificans DSM 1251(Q30PW1_SULDN), Kitasatospora setae KM-6054(E4N1M3_KITSK), Flavobacterium johnsoniae UW101(A5FJK5_FLAJ1), Citreicella sp SE45(D0DE45_9RHOB), Pelagibacterium halotolerans B2(G4RBW3_PELHB), Gillisia limnaea DSM 15749(H2BYC9_9FLAO), Phenylobacterium zucineum HLK1(B4RHN0_PHEZH), Bizionia argentinensis JUB59(G2ECI5_9FLAO), Zobellia galactanivorans(G0LBS1_ZOBGA), Rhodopseudomonas palustris CGA009(Q6N698_RHOPA), Cellulophaga lytica DSM 7489(F0RE10_CELLC), Flavobacteria bacterium BBFL7(Q26HY6_FLABB), Alkalilimnicola ehrlichii MLHE-1(Q0A5B2_ALHEH), Acetobacter pasteurianus IFO 3283-12(C7L997_ACEPA), Sphingomonas wittichii RW1(A5VB57_SPHWW), Sulfurimonas gotlandica GD1(B6BLP9_9PROT), Burkholderia sp YI23(G8M460_9BURK), Cyclobacterium marinum DSM 745(G0IZJ2_CYCMS), Flavobacteriales bacterium ALC-1(A8ULC5_9FLAO), Cellulophaga algicola DSM 14237(E6XF16_CELAD,E6XCC0_CELAD) |
| YFLV | 1,18E-11 | | P: Alicycliphilus denitrificans K601(F4GC46_ALIDK), Stenotrophomonas sp SKA14(B8L2E0_9GAMM), Acidovorax avenae subsp avenae(F0Q4M9_ACIAP), Polaromonas naphthalenivorans CJ2(A1VL14_POLNA), Herminiimonas arsenicoxydans(A4G4V5_HERAR), Hyphomonas neptunium ATCC 15444(Q0C1Z2_HYPNA), Burkholderia cenocepacia PC184(A2VXT3_9BURK), Lautropia mirabilis ATCC 51599(E7RZ21_9BURK), Roseibium sp TrichSKD4(E2CBJ4_9RHOB), Burkholderia gladioli BSR3(F2LCR3_BURGS), Comamonas testosteroni S44(D8D7D0_COMTE), Fluviicola taffensis DSM 16823(F2IAE8_FLUTR), Oceanicola batsensis HTCC2597(A3TSM3_9RHOB), Burkholderia ambifaria MEX-5(B1T3N9_9BURK), Mucilaginibacter paludis DSM 18603(H1Y0Q4_9SPHI), Burkholderia multivorans ATCC 17616(A9AG64_BURM1), Sphingobacterium sp 21(F4CFE1_SPHS2), Bordetella bronchiseptica RB50(Q7WJJ5_BORBR), Nitrosomonas eutropha C91(Q0AIE0_NITEC), Novosphingobium nitrogenifigens DSM 19370(F1Z895_9SPHN), Acidovorax citrulli AAC00-1(A1TMH1_ACIAC), Hahella chejuensis KCTC 2396(Q2SEJ4_HAHCH), Azoarcus sp BH72(A1K1I2_AZOSB), Acidovorax delafieldii 2AN(C5T6F2_ACIDE), Burkholderia glumae BGR1(C5AFG3_BURGB), Burkholderia vietnamiensis G4(A4JES7_BURVG), Janthinobacterium sp Marseille(A6SZL3_JANMA), Flavobacterium branchiophilum FL-15(G2Z6S2_FLABF), Burkholderia sp(Q39FM2_BURS3), Stenotrophomonas maltophilia K279a(B2FK64_STRMK), Acidovorax sp NO-1(H0C1D4_9BURK), Flavobacterium psychrophilum JIP0286(A6GZD3_FLAPJ), Roseovarius sp 217(A3W3T5_9RHOB), Stenotrophomonas maltophilia R551-3(B4SR62_STRM5) |
| YFLQ | 4,00E-21 | | N: Mycobacterium rhodesiae NBB3(G8RKF8_MYCRN,G8RPN1_MYCRN), Micromonas pusilla CCMP1545(C1N9B0_MICPC), Mycobacterium intracellulare MOTT-64(H8JFC0_MYCIT), Kribbella flavida DSM 17836(D2Q0K6_KRIFD), Frankia alni ACN14a(Q0RMX2_FRAAA), Mycobacterium tusciae JS617(H1JWA3_9MYCO), Haloarcula marismortui ATCC 43049(Q5UZ01_HALMA), Saccharomonospora marina XMU15(H5X4Y0_9PSEU), Mycobacterium parascrofulaceum ATCC BAA-614(D5P2P0_9MYCO), SAR116 cluster alpha proteobacterium(G5ZYB7_9PROT), Mycobacterium avium 104(A0QHM2_MYCA1), Mycobacterium bovis BCG str(G7QTX2_MYCBO), Mycobacterium vanbaalenii PYR-1(A1T9D6_MYCVP), Mycobacterium colombiense CECT 3035(F9QQ45_9MYCO), Haladaptatus paucihalophilus DX253(E7QW03_9EURY), Frankia sp EAN1pec(A8L0I3_FRASN), Mycobacterium gilvum PYR-GCK(A4TAL9_MYCGI), Perkinsus marinus ATCC 50983(C5KNH0_PERM5), Candidatus Pelagibacter ubique HTCC1062(Q4FLB5_PELUB), Desmospora sp 8437(F5SKL4_9BACL), Frankia sp CcI3(Q2J9U9_FRASC), Patulibacter medicamentivorans(H0EBK8_9ACTN), Mycobacterium sp MCS(Q1B3C2_MYCSS), Frankia sp EuI1c(E3IVK7_FRASU), halophilic archaeon DL31(G2MLY7_9ARCH), Mycobacterium smegmatis str MC2(A0R4A6_MYCS2), Frankia sp EUN1f(D3CSZ7_9ACTO), Haloarcula hispanica ATCC 33960(G0HPU0_HALHT), Halorubrum lacusprofundi ATCC 49239(B9LP51_HALLT), Thermomonospora curvata DSM 43183(D1A239_THECD), Mycobacterium ulcerans Agy99(A0PNY4_MYCUA) |
| YFLY | 1,18E-11 | | P: Flavobacteriaceae bacterium 3519-10(C6WZG4_FLAB3), Gramella forsetii KT0803(A0M173_GRAFK), Muricauda ruestringensis DSM 13258(G2PIT9_MURRD), Bdellovibrio bacteriovorus HD100(Q6MK94_BDEBA), Pedobacter heparinus DSM 2366(C6XVI0_PEDHD), Haliscomenobacter hydrossis DSM 1100(F4L2U7_HALH1), Spirosoma linguale DSM 74(D2QM53_SPILD), Niabella soli DSM 19437(H1NKD8_9SPHI,H1NM30_9SPHI), Elizabethkingia anophelis Ag1(H0KQP6_9FLAO), Riemerella anatipestifer RA-GD(F0TNT5_RIEAR), Flavobacterium johnsoniae UW101(A5FIM9_FLAJ1,A5FCK0_FLAJ1), Pedobacter saltans DSM 12145(F0SCW7_PEDSD), Fluviicola taffensis DSM 16823(F2I9I0_FLUTR), Meiothermus ruber DSM 1279(D3PTQ1_MEIRD), Chthoniobacter flavus Ellin428(B4D1F1_9BACT), Sphingobacterium spiritivorum ATCC 33861(D7VPR8_9SPHI), Leadbetterella byssophila DSM 17132(E4RVD1_LEAB4), Runella slithyformis DSM 19594(F8EAH9_RUNSL), Owenweeksia hongkongensis DSM 17368(G8R6F6_OWEHD), Myroides odoratimimus CIP 101113(H1H5B0_9FLAO), Marivirga tractuosa DSM 4126(E4TR24_MARTH), Dysgonomonas mossii DSM 22836(F8X358_9PORP), Flavobacterium frigoris PS1(H7FR52_9FLAO), unidentified eubacterium SCB49(A6ENP0_9BACT), Ktedonobacter racemifer DSM 44963(D6TP19_9CHLR), Zobellia galactanivorans(G0L0F4_ZOBGA), Chryseobacterium gleum ATCC 35910(D7VV61_9FLAO), Nitrobacter winogradskyi Nb-255(Q3SUN3_NITWN), Niastella koreensis GR20-10(G8TDR8_NIAKG), Nitrobacter hamburgensis X14(Q1QHS5_NITHX), Flavobacterium columnare ATCC 49512(G8X694_FLACA), Myroides odoratus DSM 2801(H1ZC40_9FLAO), Nitrobacter sp Nb-311A(A3WUX8_9BRAD), Weeksella virosa DSM 16922(F0P268_WEEVC), Cyclobacterium marinum DSM 745(G0J1W9_CYCMS), Gallibacterium anatis UMN179(F4H9I5_GALAU), Solitalea canadensis DSM 3403(H8KRX3_FLECA), Pedobacter sp BAL39(A6EG59_9SPHI) |
| YFHV | 6,48E-03 | | Q: Streptomyces clavuligerus ATCC 27064(B5GVJ7_STRCL), Streptomyces venezuelae ATCC 10712(F2RB89_STRVP), Nocardioidaceae bacterium Broad-1(E9UUY1_9ACTO), Yersinia ruckeri ATCC 29473(C4ULC2_YERRU), Kribbella flavida DSM 17836(D2PQN0_KRIFD), Photorhabdus asymbiotica subsp asymbiotica(B6VNR2_PHOAA), Streptomyces sp Mg1(B4V629_9ACTO), Mesorhizobium amorphae CCNWGS0123(G6Y9K6_9RHIZ), Spirosoma linguale DSM 74(D2QK90_SPILD), Mesorhizobium australicum WSM2073(G4K8I2_9RHIZ), Fluviicola taffensis DSM 16823(F2IK57_FLUTR), Frankia sp CN3(G6H1I3_9ACTO), Frankia sp EuI1c(E3J683_FRASU), Campylobacter jejuni(A0A0E2UWT7_CAMJU), Ktedonobacter racemifer DSM 44963(D6U4L4_9CHLR), Pseudoalteromonas sp SANK 73390(F8J3E6_9GAMM), Streptomyces himastatinicus ATCC 53653(D9WP19_9ACTO), Brevundimonas subvibrioides ATCC 15264(D9QFN8_BRESC), Niastella koreensis GR20-10(G8TQW4_NIAKG), Streptomyces bingchenggensis BCW-1(D7CHT6_STRBB), Mesorhizobium opportunistum WSM2075(F7YDW2_MESOW), Mesorhizobium ciceri biovar biserrulae(E8TLH5_MESCW), Frankia sp EUN1f(D3D2S0_9ACTO), Streptomyces sp C(D9VK26_9ACTO), Mesorhizobium loti MAFF303099(Q98N74_RHILO), Hylemonella gracilis ATCC 19624(F3KNL2_9BURK), Amycolatopsis mediterranei S699(G0G7F8_AMYMD), Xenorhabdus bovienii SS-2004(D3V2A9_XENBS), Solitalea canadensis DSM 3403(H8KRM2_FLECA) |
| LFFF | 2,69E-15 | | N: Halomonas sp TD01(F7SSB5_9GAMM), Congregibacter litoralis KT71(A4ACE9_9GAMM) |
| YHSL | 6,48E-03 | | O: Burkholderia pseudomallei(Q93SP0_BURPE), Cupriavidus basilensis OR16(H1S0E9_9BURK), Laribacter hongkongensis HLHK9(C1DCX8_LARHH), Acidithiobacillus ferrivorans SS3(G0JTF5_9GAMM), gamma proteobacterium HTCC5015(B5JSW2_9GAMM), Candidatus Accumulibacter phosphatis clade(C7RM33_ACCPU), Burkholderia dolosa AUO158(A2W8M7_9BURK), Cupriavidus metallidurans CH34(Q1LPZ1_RALME), Photobacterium profundum(Q6LPM9_PHOPR), Pseudoalteromonas haloplanktis TAC125(Q3IHN5_PSEHT), Shewanella pealeana ATCC 700345(A8GYI1_SHEPA), Sideroxydans lithotrophicus ES-1(D5CRB2_SIDLE), Rubrivivax benzoatilyticus JA2 =(F3LVH4_9BURK), Bdellovibrio bacteriovorus HD100(Q6MJX8_BDEBA), Photobacterium angustum S14(Q1ZQL4_PHOAS), Shewanella woodyi ATCC 51908(B1KCX2_SHEWM,B1KFI9_SHEWM), Pseudomonas mendocina NK-01(F4DUZ2_PSEMN), Herminiimonas arsenicoxydans(A4G6U6_HERAR), Shewanella loihica PV-4(A3QJE6_SHELP), Methylophaga thiooxydans DMS010(C0N247_9GAMM), Thiobacillus denitrificans ATCC 25259(Q3SKQ0_THIDA), Shewanella violacea DSS12(D4ZC95_SHEVD), Shewanella denitrificans OS217(Q12T98_SHEDO), Shewanella frigidimarina NCIMB 400(Q08A21_SHEFN), Gemmatimonas aurantiaca T-27(C1ACG4_GEMAT), Oxalobacteraceae bacterium IMCC9480(F1VZU8_9BURK), Dechloromonas aromatica RCB(Q47EI8_DECAR), Pseudogulbenkiania ferrooxidans 2002(B9Z208_9NEIS), Photobacterium leiognathi subsp mandapamensis(F2P7E9_PHOMO), Shewanella sp MR-7(Q0I0R5_SHESR), gamma proteobacterium IMCC2047(F3KDR8_9GAMM), Pseudoalteromonas sp BSi20429(G7F0K5_9GAMM), Ralstonia eutropha JMP134(Q473W9_CUPPJ), Nitrosospira multiformis ATCC 25196(Q2Y824_NITMU), Burkholderia thailandensis E264(Q2SZR5_BURTA), Shewanella oneidensis MR-1(Q8EKQ1_SHEON), Chromohalobacter salexigens DSM 3043(Q1QXX7_CHRSD), Ferrimonas balearica DSM 9799(E1SV92_FERBD), Burkholderia phymatum STM815(B2JE19_BURP8), Methylophaga aminisulfidivorans MP(F5SXI9_9GAMM), Shewanella amazonensis SB2B(A1S1K7_SHEAM), Burkholderiales bacterium JOSHI_001(H5WMW6_9BURK), Planctomyces brasiliensis DSM 5305(F0SGZ6_PLABD), Bermanella marisrubri(Q1N0I3_9GAMM), Ralstonia solanacearum(D8NAF7_RALSL), Lentisphaera araneosa HTCC2155(A6DTE6_9BACT), Shewanella sp HN-41(F7RUT6_9GAMM), Pseudomonas mendocina ymp(A4XYA7_PSEMY), Methyloversatilis universalis FAM5(F5RA92_9RHOO), Burkholderia vietnamiensis G4(A4JGG0_BURVG), Janthinobacterium sp Marseille(A6SXN6_JANMA), Thauera sp MZ1T(C4K927_THASP), Methylibium petroleiphilum PM1(A2SJB2_METPP), Neptuniibacter caesariensis(Q2BPK7_9GAMM), Thioalkalivibrio sulfidiphilus HL-EbGr7(B8GQJ2_THISH), Pseudomonas entomophila L48(Q1I987_PSEE4), Burkholderia sp CCGE1001(E8YI33_9BURK), Burkholderia sp CCGE1002(D5W5I0_BURSC), Photobacterium profundum 3TCK(Q1YXI7_PHOPR), Chromobacterium violaceum ATCC 12472(Q7NRT9_CHRVO), Polynucleobacter necessarius subsp asymbioticus(A4SYK5_POLSQ), Ralstonia syzygii R24(G2ZZY7_9RALS), Acidovorax sp NO-1(H0BSH7_9BURK), Saccharophagus degradans 2-40(Q21KH6_SACD2), Acidithiobacillus ferrooxidans ATCC 23270(B7JAC4_ACIF2), Marinomonas posidonica IVIA-Po-181(F6CYZ3_MARPP), Shewanella piezotolerans WP3(B8CHC0_SHEPW), Psychromonas ingrahamii 37(A1SZ58_PSYIN), Ralstonia pickettii 12D(C6BJV3_RALP1), Shewanella baltica OS625(G6DWT5_9GAMM), Cupriavidus taiwanensis(B3R3U0_CUPTR) |
| YFLC | 1,18E-11 | | P: Rhodococcus opacus B4(C1B1A1_RHOOB), Mycobacterium rhodesiae NBB3(G8RRI1_MYCRN), Frankia alni ACN14a(Q0RAX4_FRAAA), Mycobacterium tusciae JS617(H1K554_9MYCO), Chitinophaga pinensis DSM 2588(C7PCY5_CHIPD), Alcanivorax sp DG881(B4WYJ5_9GAMM), Xanthobacter autotrophicus Py2(A7IN60_XANP2,A7IQD1_XANP2), Legionella longbeachae NSW150(D3HKQ0_LEGLN), Legionella drancourtii LLAP12(G9EQ43_9GAMM), Burkholderia phymatum STM815(B2JG43_BURP8), Mycobacterium thermoresistibile ATCC 19527(G7CCQ9_MYCTH), Mycobacterium sp MCS(Q1B142_MYCSS), Acidiphilium multivorum AIU301(F0J070_ACIMA), Burkholderia sp H160(B5WNG3_9BURK), Frankia sp EuI1c(E3IW89_FRASU), Alcanivorax borkumensis SK2(Q0VRD9_ALCBS), Burkholderia sp CCGE1001(E8YPR1_9BURK), Burkholderia sp CCGE1002(D5W7R5_BURSC), Burkholderia sp CCGE1003(E1T6S0_BURSG), Rhodococcus jostii RHA1(Q0S323_RHOSR), Flavobacterium columnare ATCC 49512(G8X6A5_FLACA), Burkholderia rhizoxinica HKI 454(E5AQQ1_BURRH), Burkholderia xenovorans LB400(Q13Z95_BURXL), Mycobacterium ulcerans Agy99(A0PR02_MYCUA) |
| YFAQ | 9,04E-03 | | O: Vitis vinifera(D7SUH5_VITVI), Brevibacillus brevis NBRC 100599(C0ZIH8_BREBN), Thermobacillus composti KWC4(G4EHA5_9BACL), Paenibacillus mucilaginosus KNP414(F8FDK4_PAEMK), Paenibacillus dendritiformis C454(H3SCH5_9BACL), Paenibacillus terrae HPL-003(G7VWP9_PAETH), Brevibacillus laterosporus GI-9(H0UG21_BRELA), Carica papaya(C4PB37_CARPA), Arabidopsis thaliana(Q946U7_ARATH), Zea mays(B6SXE2_MAIZE), Medicago truncatula(G7J2X4_MEDTR), Leptospira biflexa serovar Patoc(B0SLD6_LEPBP), Paenibacillus polymyxa E681(E0RCP4_PAEP6), Wolffia australiana(H6U807_9ARAE), Physcomitrella patens(A9S385_PHYPA,A9TSV7_PHYPA), Arabidopsis lyrata subsp lyrata(D7M9Q6_ARALL), Paenibacillus sp HGF5(F3M7Q5_9BACL), Paenibacillus sp HGF7(F5LGN1_9BACL), Desmospora sp 8437(F5SFV7_9BACL), Paenibacillus curdlanolyticus YK9(E0I6V1_9BACL), Selaginella moellendorffii(D8S6W9_SELML), Paenibacillus sp JDR-2(C6CW20_PAESJ), Picea sitchensis(A9NND9_PICSI), Triticum aestivum(B5TQZ2_WHEAT), Paenibacillus sp oral taxon(C6J171_9BACL), Datisca glomerata(Q7Y079_DATGL), Populus trichocarpa(B9H9D4_POPTR), Bacillus sp 1NLA3E(H1I1M8_9BACI), Eutrema halophilum(E4MXS1_THEHA), Thalassiosira pseudonana(B8C835_THAPS), Oryza sativa Japonica Group(Q69XE4_ORYSJ), Glycine max(Q6QDC2_SOYBN,C6T2S6_SOYBN), Paenibacillus larvae subsp larvae(E7MC96_9BACL), Paenibacillus sp Aloe-11(H6CNJ3_9BACL), Ricinus communis(B9RS68_RICCO), Bacillus coagulans 36D1(G2TMS0_BACCO), Phaeodactylum tricornutum CCAP 10551(B7G0D0_PHATC), Kyrpidia tusciae DSM 2912(D5WU47_BACT2), Paenibacillus lactis 154(G4HLD4_9BACL) |
| LFFL | 2,69E-15 | | N: Shewanella denitrificans OS217(Q12SA8_SHEDO), Marinobacter manganoxydans MnI7-9(G6YYX6_9ALTE), Shewanella sp MR-4(Q0HLA8_SHESM), Marinobacter adhaerens HP15(E4PI43_MARAH), Shewanella amazonensis SB2B(A1SAB3_SHEAM), Reinekea blandensis MED297(A4BB88_9GAMM), Marinobacter sp ELB17(A3JBC8_9ALTE), Marinobacter algicola DG893(A6EY49_9ALTE), Rheinheimera sp A13L(F7NXD1_9GAMM), Saccharophagus degradans 2-40(Q21LC5_SACD2), Halomonas sp HAL1(G4F309_9GAMM) |
| YFMK | 2,69E-15 | | P: Rhizobium leguminosarum bv trifolii(B5ZXR3_RHILW), Agrobacterium sp ATCC 31749(F5J5A9_9RHIZ), Agrobacterium tumefaciens F2(F7U441_RHIRD), Sinorhizobium medicae WSM419(A6ULS3_SINMW), Agrobacterium tumefaciens CCNWGS0286(G6XXM2_RHIRD), Sinorhizobium fredii NGR234(C3KMT5_RHISN), Rhizobium sp PDO1-076(H4F0B9_9RHIZ), Rhizobium etli CFN 42(Q2KDK7_RHIEC), Agrobacterium tumefaciens 5A(H0HAA1_RHIRD), Rhizobium etli CIAT 652(B3PYD4_RHIE6), Rhizobium leguminosarum bv viciae(Q1MMQ0_RHIL3), Sinorhizobium meliloti CCNWSX0020(H0FT84_RHIML) |
| LFLL | 2,78E-07 | | N: Pseudomonas psychrotolerans L19(H0JJY3_9PSED), Haliscomenobacter hydrossis DSM 1100(F4L646_HALH1), Pseudomonas syringae Cit 7(F3GTA3_PSESX), Pseudomonas syringae pv oryzae(F2ZGU5_9PSED), Pseudomonas syringae pv maculicola(F3HFH5_PSEYM), Idiomarina sp A28L(F7RV81_9GAMM), Parvularcula bermudensis HTCC2503(E0TBW6_PARBH), Pseudomonas fulva 12-X(F6AA16_PSEF1), mine drainage metagenome(E6PUT6_9ZZZZ), Pseudomonas savastanoi pv savastanoi(D7I4P2_PSESS), Pseudomonas fluorescens SBW25(C3K7I0_PSEFS), Xanthomonas oryzae pv oryzae(Q5GXS7_XANOR), Xanthomonas albilineans GPE PC73(D2UCQ4_XANAP), Pseudomonas mendocina ymp(A4XRM8_PSEMY), Salinisphaera shabanensis E1L3A(F7QD61_9GAMM), Xanthomonas gardneri ATCC 19865(F0C5U4_9XANT), Xanthomonas campestris pv campestris(B0RR93_XANCB), Pseudomonas syringae pv aptata(F3J5N9_PSEAP), Pseudomonas syringae pv morsprunorum(F3DZ23_9PSED), Pseudomonas syringae pv tomato(E2MJY0_PSEUB), Xanthomonas vesicatoria ATCC 35937(F0BCB2_9XANT), Ricinus communis(B9TG83_RICCO), Xanthomonas citri pv mangiferaeindicae(H8FET1_XANCI) |
| YHML | 2,28E-04 | | O: Novosphingobium aromaticivorans DSM 12444(Q2GCA5_NOVAD), Novosphingobium pentaromativorans US6-1(G6EBK2_9SPHN), Sphingobium sp SYK-6(G2ISN4_9SPHN), Caulobacter segnis ATCC 21756(D5VQ98_CAUST), Novosphingobium sp PP1Y(F6IFD3_9SPHN), Caulobacter sp K31(B0T7B3_CAUSK), Novosphingobium nitrogenifigens DSM 19370(F1Z5X0_9SPHN), Sphingobium chlorophenolicum L-1(F6ESW7_SPHCR), Sphingomonas wittichii RW1(A5V5K0_SPHWW), marine gamma proteobacterium HTCC2148(B7RWE6_9GAMM) |
| VFLL | 1,18E-11 | | N: Acidithiobacillus caldus SM-1(F9ZLS8_ACICS,F9ZPY0_ACICS), Limnobacter sp MED105(A6GNQ3_9BURK), Halothiobacillus neapolitanus c2(D0L0N5_HALNC), Hyphomonas neptunium ATCC 15444(Q0BZ22_HYPNA), Oxalobacteraceae bacterium IMCC9480(F1VZN5_9BURK), Acidiphilium cryptum JF-5(A5G112_ACICJ), Burkholderiales bacterium JOSHI_001(H5WPM9_9BURK), Brevundimonas subvibrioides ATCC 15264(D9QNS8_BRESC), Cupriavidus necator N-1(F8GPA5_CUPNN), Ramlibacter tataouinensis TTB310(F5Y5W2_RAMTT), Methylibium petroleiphilum PM1(A2SF55_METPP), Curvibacter putative symbiont of(C9Y9G0_9BURK), Candidatus Nitrospira defluvii(D8PAF5_9BACT,D8P7L8_9BACT), Acidovorax sp NO-1(H0C3W6_9BURK) |
| YHSF | 6,48E-03 | | O: Rhizobium leguminosarum bv trifolii(B5ZZM9_RHILW), Agrobacterium sp ATCC 31749(F5J978_9RHIZ), Agrobacterium tumefaciens F2(F7U5G1_RHIRD), Sinorhizobium medicae WSM419(A6UBY2_SINMW), Limnobacter sp MED105(A6GP10_9BURK), Agrobacterium vitis S4(B9JR55_AGRVS), Asticcacaulis excentricus CB 48(E8RL60_ASTEC), Sinorhizobium fredii NGR234(C3MGD5_RHISN), Rhizobium sp PDO1-076(H4FB18_9RHIZ), Rhizobium etli CFN 42(Q2K563_RHIEC), Collimonas fungivorans Ter331(G0ACD4_COLFT), Agrobacterium tumefaciens 5A(H0H881_RHIRD), Rhizobium etli CIAT 652(B3PX70_RHIE6), Rhizobium leguminosarum bv viciae(Q1MD01_RHIL3), Agrobacterium radiobacter K84(B9J857_AGRRK), Burkholderia xenovorans LB400(Q142K1_BURXL), Sinorhizobium meliloti CCNWSX0020(H0GA12_RHIML) |
| YHAL | 6,48E-03 | | O: Methylotenera versatilis 301(D7DIW7_METS0), Comamonas testosteroni ATCC 11996(H1RK32_COMTE), Bordetella petrii DSM 12804(A9IJT5_BORPD), Methylobacter tundripaludum SV96(G3IT70_9GAMM), Variovorax paradoxus EPS(E6UXE4_VARPE), Alicycliphilus denitrificans K601(F4GAB4_ALIDK), Variovorax paradoxus S110(C5CWP3_VARPS), Methylomonas methanica MC09(G0A2B2_METMM), Methylomicrobium alcaliphilum 20Z(G4T327_META2), Polaromonas sp JS666(Q12EZ1_POLSJ), Novosphingobium aromaticivorans DSM 12444(Q2G523_NOVAD), Acidovorax avenae subsp avenae(F0Q0V5_ACIAP), Marinomonas mediterranea MMB-1(F2JVC2_MARM1), Neisseria wadsworthii 9715(G4CMK5_9NEIS), Polaromonas naphthalenivorans CJ2(A1VKI6_POLNA), Rhodoferax ferrireducens T118(Q21TE0_RHOFD), Methylovorus sp MP688(E4QIY5_METS6), Methylobacillus flagellatus KT(Q1H1W3_METFK), Methylococcus capsulatus str Bath(Q606Y3_METCA), Gallionella capsiferriformans ES-2(D9SK81_GALCS), Achromobacter arsenitoxydans SY8(H0F9F5_9BURK), Achromobacter xylosoxidans C54(E5U1W4_ALCXX), Beijerinckia indica subsp indica(B2IE39_BEII9), Azotobacter vinelandii DJ(C1DDP7_AZOVD), Achromobacter xylosoxidans A8(E3HFM9_ACHXA), Acidovorax ebreus TPSY(B9MEH6_ACIET), Bradyrhizobium sp STM 3843(H0TRL7_9BRAD), Sphingomonas sp SKA58(Q1NF48_9SPHN), Bordetella avium 197N(Q2KYN5_BORA1), Novosphingobium nitrogenifigens DSM 19370(F1ZB73_9SPHN), Mariprofundus ferrooxydans PV-1(Q0F2H6_9PROT), Achromobacter piechaudii ATCC 43553(D4XAA8_9BURK), Azoarcus sp BH72(A1K669_AZOSB), Acidovorax delafieldii 2AN(C5TD07_ACIDE), Leptothrix cholodnii SP-6(B1Y0J1_LEPCP), Azoarcus sp KH32C(H0PVQ4_9RHOO), Burkholderia vietnamiensis G4(A4JRN5_BURVG), Methylomicrobium album BG8(H8GH79_METAL), Ramlibacter tataouinensis TTB310(F5XW43_RAMTT), Methylotenera mobilis JLW8(C6WW87_METML), Sphingobium chlorophenolicum L-1(F6F2Z0_SPHCR), Herbaspirillum seropedicae SmR1(D8IZK7_HERSS), Curvibacter putative symbiont of(C9Y8P3_9BURK), Sphingobium japonicum UT26S(D4Z631_SPHJU), Bacteriovorax marinus SJ(E1WZL7_BACMS), Achromobacter xylosoxidans AXX-A(F7T4L5_ALCXX), Acidovorax sp NO-1(H0BTR3_9BURK), Bordetella pertussis Tohama I(Q7VWB0_BORPE), Methylomonas sp 16a(A3QVH5_9GAMM), Delftia acidovorans SPH-1(A9BT93_DELAS), Hylemonella gracilis ATCC 19624(F3KVD3_9BURK) |
|  |  |  |  |
|  |  |  |  |
|  |  |  |  |
| STG8 key residues H9-G8-G9 | $\boldsymbol{k}_{\boldsymbol{STG}\boldsymbol{8}}$  **[**$\boldsymbol{M}^{\boldsymbol{-1}}\boldsymbol{s}^{\boldsymbol{-1}}$**]** | | **Specie (uniprotID)** |
| FWL | 2,69E-15 | | O: Burkholderia pseudomallei(Q93SP0_BURPE), Vitis vinifera(D7SUH5_VITVI), Cupriavidus basilensis OR16(H1S0E9_9BURK), Acidithiobacillus ferrivorans SS3(G0JTF5_9GAMM), Variovorax paradoxus EPS(E6UXE4_VARPE), Burkholderia dolosa AUO158(A2W8M7_9BURK), Limnobacter sp MED105(A6GP10_9BURK), Sideroxydans lithotrophicus ES-1(D5CRB2_SIDLE), Variovorax paradoxus S110(C5CWP3_VARPS), Polaromonas sp JS666(Q12EZ1_POLSJ), Janibacter sp HTCC2649(A3TFS8_9MICO), Polaromonas naphthalenivorans CJ2(A1VKI6_POLNA,A1VPS5_POLNA), Herminiimonas arsenicoxydans(A4G6U6_HERAR), Methylophaga thiooxydans DMS010(C0N247_9GAMM), Arthrobacter arilaitensis Re117(E1VWS4_ARTAR), Rhodoferax ferrireducens T118(Q21TE0_RHOFD), Oxalobacteraceae bacterium IMCC9480(F1VZU8_9BURK), Carica papaya(C4PB37_CARPA), Arabidopsis thaliana(Q946U7_ARATH), Gallionella capsiferriformans ES-2(D9SK81_GALCS), Pseudogulbenkiania ferrooxidans 2002(B9Z208_9NEIS), Ralstonia eutropha JMP134(Q473W9_CUPPJ), mine drainage metagenome(E6PT94_9ZZZZ), Nitrosospira multiformis ATCC 25196(Q2Y824_NITMU), Burkholderia thailandensis E264(Q2SZR5_BURTA), Methylosinus trichosporium OB3b(D5QKG5_METTR), Algoriphagus machipongonensis(A3I308_9BACT), Zea mays(B6SXE2_MAIZE), Medicago truncatula(G7J2X4_MEDTR), Wolffia australiana(H6U807_9ARAE), Beijerinckia indica subsp indica(B2IE39_BEII9), Physcomitrella patens(A9S385_PHYPA,A9TSV7_PHYPA), Arabidopsis lyrata subsp lyrata(D7M9Q6_ARALL), Burkholderia phymatum STM815(B2JE19_BURP8), Methylophaga aminisulfidivorans MP(F5SXI9_9GAMM), Thiomonas intermedia K12(D5X1M5_THIK1), Burkholderiales bacterium JOSHI_001(H5WW47_9BURK), Selaginella moellendorffii(D8S6W9_SELML), Planctomyces brasiliensis DSM 5305(F0SGZ6_PLABD), Picea sitchensis(A9NND9_PICSI), Triticum aestivum(B5TQZ2_WHEAT), Kocuria rhizophila DC2201(B2GG22_KOCRD), Beggiatoa sp PS(A7BTI1_9GAMM), Corynebacterium glucuronolyticum ATCC 51866(C2GJU6_9CORY), Collimonas fungivorans Ter331(G0ACD4_COLFT), Caldalkalibacillus thermarum TA2A1(F5L510_9BACI), Saccharopolyspora erythraea NRRL 2338(A4F9C9_SACEN), Azoarcus sp BH72(A1K669_AZOSB), Datisca glomerata(Q7Y079_DATGL), Populus trichocarpa(B9H9D4_POPTR), Eutrema halophilum(E4MXS1_THEHA), Leptothrix cholodnii SP-6(B1Y0J1_LEPCP), Azoarcus sp KH32C(H0PVQ4_9RHOO), Oryza sativa Japonica Group(Q69XE4_ORYSJ), Burkholderia vietnamiensis G4(A4JGG0_BURVG), Planctomyces maris DSM 8797(A6CB44_9PLAN), Glycine max(Q6QDC2_SOYBN,C6T2S6_SOYBN), Janthinobacterium sp Marseille(A6SXN6_JANMA), Ramlibacter tataouinensis TTB310(F5XW43_RAMTT), Methylibium petroleiphilum PM1(A2SJB2_METPP), Kytococcus sedentarius DSM 20547(C7NFL3_KYTSD), Thioalkalivibrio sulfidiphilus HL-EbGr7(B8GQJ2_THISH), Burkholderia sp CCGE1001(E8YI33_9BURK), Burkholderia sp CCGE1002(D5W5I0_BURSC), Herbaspirillum seropedicae SmR1(D8IZK7_HERSS), Chromobacterium violaceum ATCC 12472(Q7NRT9_CHRVO), Polynucleobacter necessarius subsp asymbioticus(A4SYK5_POLSQ), Curvibacter putative symbiont of(C9Y8P3_9BURK), Bacteriovorax marinus SJ(E1WZL7_BACMS), Burkholderia xenovorans LB400(Q142K1_BURXL,Q13N39_BURXL), Acidovorax sp NO-1(H0BSH7_9BURK), Acidithiobacillus ferrooxidans ATCC 23270(B7JAC4_ACIF2), Ricinus communis(B9RS68_RICCO), Methylocella silvestris BL2(B8EJ43_METSB), Ralstonia pickettii 12D(C6BJV3_RALP1), Brevibacterium mcbrellneri ATCC 49030(D4YQV3_9MICO), Cupriavidus taiwanensis(B3R3U0_CUPTR), Acidothermus cellulolyticus 11B(A0LVC1_ACIC1) |
| FWL | 2,69E-15 | | Q: Streptomyces venezuelae ATCC 10712(F2RB89_STRVP), Nocardioidaceae bacterium Broad-1(E9UUY1_9ACTO), Frankia sp CN3(G6H1I3_9ACTO), Frankia sp EuI1c(E3J683_FRASU), Streptomyces himastatinicus ATCC 53653(D9WP19_9ACTO), Streptomyces bingchenggensis BCW-1(D7CHT6_STRBB), Frankia sp EUN1f(D3D2S0_9ACTO) |
| LVV | 1,22E-03 | | N: Mycobacterium rhodesiae NBB3(G8RPN1_MYCRN), Mycobacterium intracellulare MOTT-64(H8JFC0_MYCIT), Kribbella flavida DSM 17836(D2Q0K6_KRIFD), Frankia alni ACN14a(Q0RMX2_FRAAA), Mycobacterium tusciae JS617(H1JWA3_9MYCO), Mycobacterium parascrofulaceum ATCC BAA-614(D5P2P0_9MYCO), SAR116 cluster alpha proteobacterium(G5ZYB7_9PROT), Mycobacterium avium 104(A0QHM2_MYCA1), Mycobacterium bovis BCG str(G7QTX2_MYCBO), Mycobacterium vanbaalenii PYR-1(A1T9D6_MYCVP), Mycobacterium colombiense CECT 3035(F9QQ45_9MYCO), Frankia sp EAN1pec(A8L0I3_FRASN), Mycobacterium gilvum PYR-GCK(A4TAL9_MYCGI), Halomonas sp TD01(F7SSB5_9GAMM), Frankia sp CcI3(Q2J9U9_FRASC), Patulibacter medicamentivorans(H0EBK8_9ACTN), Congregibacter litoralis KT71(A4ACE9_9GAMM), Frankia sp EuI1c(E3IVK7_FRASU), Mycobacterium smegmatis str MC2(A0R4A6_MYCS2), Frankia sp EUN1f(D3CSZ7_9ACTO) |
| AWV | 2,69E-15 | | O: Novosphingobium aromaticivorans DSM 12444(Q2GCA5_NOVAD), Novosphingobium pentaromativorans US6-1(G6EBK2_9SPHN), Novosphingobium sp PP1Y(F6IFD3_9SPHN), Novosphingobium nitrogenifigens DSM 19370(F1Z5X0_9SPHN) |
| AWV | 2,69E-15 | | P: Rhodococcus opacus B4(C1B1A1_RHOOB), Streptomyces venezuelae ATCC 10712(F2R152_STRVP), Acidovorax avenae subsp avenae(F0Q4M9_ACIAP), Kitasatospora setae KM-6054(E4N1M3_KITSK), Flavobacterium johnsoniae UW101(A5FCK0_FLAJ1), Acidovorax citrulli AAC00-1(A1TMH1_ACIAC), Acidovorax delafieldii 2AN(C5T6F2_ACIDE), Rhodococcus jostii RHA1(Q0S323_RHOSR), Acidovorax sp NO-1(H0C1D4_9BURK) |
| AWV | 2,69E-15 | | Q: Streptomyces clavuligerus ATCC 27064(B5GVJ7_STRCL), Yersinia ruckeri ATCC 29473(C4ULC2_YERRU), Kribbella flavida DSM 17836(D2PQN0_KRIFD), Photorhabdus asymbiotica subsp asymbiotica(B6VNR2_PHOAA), Fluviicola taffensis DSM 16823(F2IK57_FLUTR), Ktedonobacter racemifer DSM 44963(D6U4L4_9CHLR), Pseudoalteromonas sp SANK 73390(F8J3E6_9GAMM), Niastella koreensis GR20-10(G8TQW4_NIAKG), Amycolatopsis mediterranei S699(G0G7F8_AMYMD), Xenorhabdus bovienii SS-2004(D3V2A9_XENBS) |
| LWV | 2,69E-15 | | O: Sphingomonas sp SKA58(Q1NF48_9SPHN), Sphingobium chlorophenolicum L-1(F6F2Z0_SPHCR), Sphingomonas wittichii RW1(A5V5K0_SPHWW), Sphingobium japonicum UT26S(D4Z631_SPHJU) |
| RWL | 2,69E-15 | | P: Oceanicaulis sp HTCC2633(A3UC56_9RHOB), Hyphomonas neptunium ATCC 15444(Q0C302_HYPNA), Maricaulis maris MCS10(Q0ARP0_MARMM), Hirschia baltica ATCC 49814(C6XNL3_HIRBI), Alkalilimnicola ehrlichii MLHE-1(Q0A5B2_ALHEH) |
| LLV | 2,28E-04 | | N: Acidithiobacillus caldus SM-1(F9ZLS8_ACICS,F9ZPY0_ACICS), Limnobacter sp MED105(A6GNQ3_9BURK), Halothiobacillus neapolitanus c2(D0L0N5_HALNC), Pseudomonas psychrotolerans L19(H0JJY3_9PSED), Haliscomenobacter hydrossis DSM 1100(F4L646_HALH1), Hyphomonas neptunium ATCC 15444(Q0BZ22_HYPNA), Pseudomonas syringae Cit 7(F3GTA3_PSESX), Shewanella denitrificans OS217(Q12SA8_SHEDO), Pseudomonas syringae pv oryzae(F2ZGU5_9PSED), Oxalobacteraceae bacterium IMCC9480(F1VZN5_9BURK), Marinobacter manganoxydans MnI7-9(G6YYX6_9ALTE), Pseudomonas syringae pv maculicola(F3HFH5_PSEYM), Acidiphilium cryptum JF-5(A5G112_ACICJ), Idiomarina sp A28L(F7RV81_9GAMM), Parvularcula bermudensis HTCC2503(E0TBW6_PARBH), Shewanella sp MR-4(Q0HLA8_SHESM), Pseudomonas fulva 12-X(F6AA16_PSEF1), Marinobacter adhaerens HP15(E4PI43_MARAH), Pseudomonas savastanoi pv savastanoi(D7I4P2_PSESS), Shewanella amazonensis SB2B(A1SAB3_SHEAM), Burkholderiales bacterium JOSHI_001(H5WPM9_9BURK), Reinekea blandensis MED297(A4BB88_9GAMM), Pseudomonas fluorescens SBW25(C3K7I0_PSEFS), Xanthomonas oryzae pv oryzae(Q5GXS7_XANOR), Xanthomonas albilineans GPE PC73(D2UCQ4_XANAP), Pseudomonas mendocina ymp(A4XRM8_PSEMY), Brevundimonas subvibrioides ATCC 15264(D9QNS8_BRESC), Salinisphaera shabanensis E1L3A(F7QD61_9GAMM), Cupriavidus necator N-1(F8GPA5_CUPNN), Marinobacter sp ELB17(A3JBC8_9ALTE), Ramlibacter tataouinensis TTB310(F5Y5W2_RAMTT), Methylibium petroleiphilum PM1(A2SF55_METPP), Marinobacter algicola DG893(A6EY49_9ALTE), Rheinheimera sp A13L(F7NXD1_9GAMM), Curvibacter putative symbiont of(C9Y9G0_9BURK), Xanthomonas gardneri ATCC 19865(F0C5U4_9XANT), Xanthomonas campestris pv campestris(B0RR93_XANCB), Candidatus Nitrospira defluvii(D8PAF5_9BACT,D8P7L8_9BACT), Pseudomonas syringae pv aptata(F3J5N9_PSEAP), Pseudomonas syringae pv morsprunorum(F3DZ23_9PSED), Pseudomonas syringae pv tomato(E2MJY0_PSEUB), Acidovorax sp NO-1(H0C3W6_9BURK), Xanthomonas vesicatoria ATCC 35937(F0BCB2_9XANT), Saccharophagus degradans 2-40(Q21LC5_SACD2), Ricinus communis(B9TG83_RICCO), Halomonas sp HAL1(G4F309_9GAMM), Xanthomonas citri pv mangiferaeindicae(H8FET1_XANCI) |
|  |  |  |  |
| LT key residues H5-B2-H9-E15-E11-G8 | $\boldsymbol{k}_{\boldsymbol{LT}}$  **[**$\boldsymbol{M}^{\boldsymbol{-1}}\boldsymbol{s}^{\boldsymbol{-1}}$**]** | | **Specie (uniprotID)** |
| LFFLLW | 2,21E-12 | | O: Janibacter sp HTCC2649(A3TFS8_9MICO), Arthrobacter arilaitensis Re117(E1VWS4_ARTAR), Kocuria rhizophila DC2201(B2GG22_KOCRD), Corynebacterium glucuronolyticum ATCC 51866(C2GJU6_9CORY), Saccharopolyspora erythraea NRRL 2338(A4F9C9_SACEN), Kytococcus sedentarius DSM 20547(C7NFL3_KYTSD), Brevibacterium mcbrellneri ATCC 49030(D4YQV3_9MICO), Acidothermus cellulolyticus 11B(A0LVC1_ACIC1) |
| FILLQW | 4,26E-05 | | O: Leptospira biflexa serovar Patoc(B0SLD6_LEPBP) |
| LILLQW | 6,48E-03 | | O: Paenibacillus mucilaginosus KNP414(F8FDK4_PAEMK), Paenibacillus dendritiformis C454(H3SCH5_9BACL), Bacillus clausii KSM-K16(Q5WF01_BACSK), Desmospora sp 8437(F5SFV7_9BACL), Paenibacillus sp oral taxon(C6J171_9BACL), Bacillus megaterium QM B1551(D5E038_BACMQ), Bacillus cytotoxicus NVH 391-98(A7GM87_BACCN), Bacillus cereus Rock3-44(C2W574_BACCE), Paenibacillus larvae subsp larvae(E7MC96_9BACL), Paenibacillus sp Aloe-11(H6CNJ3_9BACL), Geobacillus sp WCH70(C5D799_GEOSW), Bacillus thuringiensis serovar huazhongensis(C3GXW6_BACTU), Paenibacillus lactis 154(G4HLD4_9BACL) |
| LFFLQW | 4,26E-05 | | O: Zea mays(B6SXE2_MAIZE), Medicago truncatula(G7J2X4_MEDTR), Triticum aestivum(B5TQZ2_WHEAT), Thalassiosira pseudonana(B8C835_THAPS), Oryza sativa Japonica Group(Q69XE4_ORYSJ) |
| LIAFQV | 3,37E-02 | | N: Mycobacterium ulcerans Agy99(A0PNY4_MYCUA) |
| LFLLFW | 6,33E-11 | | O: Arthrobacter aurescens TC1(A1R7B2_ARTAT), Streptomyces sp AA4(D9VAR2_9ACTO), Arthrobacter sp FB24(A0JXM3_ARTS2), Streptomyces clavuligerus ATCC 27064(B5GVB3_STRCL), Arthrobacter chlorophenolicus A6(B8HA44_ARTCA), Kribbella flavida DSM 17836(D2PTJ8_KRIFD), Streptomyces sp Mg1(B4V9I1_9ACTO), Dermacoccus sp Ellin185(E3BBA3_9MICO), Streptomyces albus J1074(D6B196_9ACTO), Kitasatospora setae KM-6054(E4NIA7_KITSK), Streptomyces sp SirexAA-E(G2NKL6_9ACTO), Streptomyces griseus XylebKG-1(G0PQ09_STRGR), Streptosporangium roseum DSM 43021(D2BDZ5_STRRD), Amycolicicoccus subflavus DQS3-9A1(F6EQ94_AMYSD), Streptomyces avermitilis MA-4680 =(Q82CH9_STRAW), Actinosynnema mirum DSM 43827(C6WQ34_ACTMD), Arthrobacter phenanthrenivorans Sphe3(F0M4E0_ARTPP), Streptomyces sp SPB74(B5G7N3_9ACTO), Microlunatus phosphovorus NM-1(F5XP72_MICPN), Streptomyces pratensis ATCC 33331(E8W9Z7_STRFA), Arthrobacter globiformis NBRC 12137(H0QPD5_ARTGO), Streptomyces coelicolor A3(2)(Q9L250_STRCO), Streptomyces sp C(D9VWG0_9ACTO), Amycolatopsis mediterranei S699(G0G0V5_AMYMD) |
| LIAEVW | 1,22E-03 | | P: Roseibium sp TrichSKD4(E2CBJ4_9RHOB), Mucilaginibacter paludis DSM 18603(H1Y0Q4_9SPHI), Sphingobacterium sp 21(F4CFE1_SPHS2), Leadbetterella byssophila DSM 17132(E4RWM0_LEAB4), Hahella chejuensis KCTC 2396(Q2SEJ4_HAHCH) |
| LILLLL | 1,22E-03 | | N: Haliscomenobacter hydrossis DSM 1100(F4L646_HALH1), Pseudomonas syringae Cit 7(F3GTA3_PSESX), Shewanella denitrificans OS217(Q12SA8_SHEDO), Pseudomonas syringae pv oryzae(F2ZGU5_9PSED), Marinobacter manganoxydans MnI7-9(G6YYX6_9ALTE), Pseudomonas syringae pv maculicola(F3HFH5_PSEYM), Idiomarina sp A28L(F7RV81_9GAMM), Parvularcula bermudensis HTCC2503(E0TBW6_PARBH), Marinobacter adhaerens HP15(E4PI43_MARAH), Pseudomonas savastanoi pv savastanoi(D7I4P2_PSESS), Pseudomonas mendocina ymp(A4XRM8_PSEMY), Salinisphaera shabanensis E1L3A(F7QD61_9GAMM), Cupriavidus necator N-1(F8GPA5_CUPNN), Marinobacter sp ELB17(A3JBC8_9ALTE), Marinobacter algicola DG893(A6EY49_9ALTE), Candidatus Nitrospira defluvii(D8P7L8_9BACT), Pseudomonas syringae pv aptata(F3J5N9_PSEAP), Pseudomonas syringae pv morsprunorum(F3DZ23_9PSED), Pseudomonas syringae pv tomato(E2MJY0_PSEUB), Acidovorax sp NO-1(H0C3W6_9BURK), Halomonas sp HAL1(G4F309_9GAMM) |
| LILLLW | 1,49E-06 | | O: Methylotenera versatilis 301(D7DIW7_METS0), gamma proteobacterium HTCC5015(B5JSW2_9GAMM), Methylobacter tundripaludum SV96(G3IT70_9GAMM), Pseudoalteromonas haloplanktis TAC125(Q3IHN5_PSEHT), Shewanella pealeana ATCC 700345(A8GYI1_SHEPA), Methylomonas methanica MC09(G0A2B2_METMM), Marinomonas mediterranea MMB-1(F2JVC2_MARM1), Shewanella woodyi ATCC 51908(B1KCX2_SHEWM), Shewanella loihica PV-4(A3QJE6_SHELP), Thiobacillus denitrificans ATCC 25259(Q3SKQ0_THIDA), Methylovorus sp MP688(E4QIY5_METS6), Shewanella denitrificans OS217(Q12T98_SHEDO), Shewanella frigidimarina NCIMB 400(Q08A21_SHEFN), Gemmatimonas aurantiaca T-27(C1ACG4_GEMAT), Xanthobacter autotrophicus Py2(A7IBJ8_XANP2), Shewanella sp MR-7(Q0I0R5_SHESR), gamma proteobacterium IMCC2047(F3KDR8_9GAMM), Pseudoalteromonas sp BSi20429(G7F0K5_9GAMM), Shewanella oneidensis MR-1(Q8EKQ1_SHEON), Shewanella amazonensis SB2B(A1S1K7_SHEAM), Bermanella marisrubri(Q1N0I3_9GAMM), Shewanella sp HN-41(F7RUT6_9GAMM), Methylotenera mobilis JLW8(C6WW87_METML), marine gamma proteobacterium HTCC2148(B7RWE6_9GAMM), marine gamma proteobacterium HTCC2143(A0YE49_9GAMM), Saccharophagus degradans 2-40(Q21KH6_SACD2), Shewanella piezotolerans WP3(B8CHC0_SHEPW), Methylomonas sp 16a(A3QVH5_9GAMM), Planctomyces limnophilus DSM 3776(D5SYN7_PLAL2), Psychromonas ingrahamii 37(A1SZ58_PSYIN), Shewanella baltica OS625(G6DWT5_9GAMM) |
| LILSLL | 1,49E-06 | | N: Acidithiobacillus caldus SM-1(F9ZPY0_ACICS), Limnobacter sp MED105(A6GNQ3_9BURK), Halothiobacillus neapolitanus c2(D0L0N5_HALNC), Burkholderiales bacterium JOSHI_001(H5WPM9_9BURK), Candidatus Nitrospira defluvii(D8PAF5_9BACT) |
| LIALLW | 4,26E-05 | | O: Novosphingobium aromaticivorans DSM 12444(Q2G523_NOVAD), Sphingobium sp SYK-6(G2ISN4_9SPHN), Novosphingobium nitrogenifigens DSM 19370(F1ZB73_9SPHN) |
| LFLLLW | 1,49E-06 | | O: Mycobacterium tuberculosis NCGM2209(G2UUQ6_MYCTU), Clavibacter michiganensis subsp sepedonicus(B0RAR2_CLAMS), Micrococcus luteus SK58(D3LL05_MICLU), Corynebacterium efficiens YS-314(Q8FN04_COREF), Streptomyces sviceus ATCC 29083(D6XBM2_9ACTO), Microbacterium laevaniformans OR221(H8E4L0_9MICO), Mycobacterium rhodesiae NBB3(G8RIA5_MYCRN), Leifsonia xyli subsp xyli(Q6AFT0_LEIXX), Actinoplanes sp SE50110(G8S1V0_ACTS5), Mycobacterium intracellulare MOTT-64(H8JRE9_MYCIT), Cellulomonas flavigena DSM 20109(D5UCE3_CELFN), Corynebacterium glutamicum ATCC 14067(G6WV80_CORGT), Streptomyces venezuelae ATCC 10712(F2R2J2_STRVP), Blastococcus saxobsidens DD2(H6RIR9_BLASD), Xylanimonas cellulosilytica DSM 15894(D1BZH5_XYLCX), Gordonia effusa NBRC 100432(H0QZ84_9ACTO), Streptomyces zinciresistens K42(G2GKZ3_9ACTO), Beutenbergia cavernae DSM 12333(C5BXJ9_BEUC1), Frankia alni ACN14a(Q0RPN6_FRAAA), Segniliparus rotundus DSM 44985(D6Z7C0_SEGRD), Mycobacterium tusciae JS617(H1JSN5_9MYCO), Corynebacterium amycolatum SK46(E2MXD8_9CORY), Corynebacterium variabile DSM 44702(G0HB35_CORVD), Corynebacterium accolens ATCC 49725(C0WJJ8_9CORY), Saccharomonospora marina XMU15(H5X3G1_9PSEU), Mycobacterium parascrofulaceum ATCC BAA-614(D5PH46_9MYCO), Mycobacterium avium 104(A0QDE2_MYCA1), Streptomyces ghanaensis ATCC 14672(D6A297_9ACTO), Mycobacterium vanbaalenii PYR-1(A1TCE3_MYCVP), Nocardia farcinica IFM 10152(Q5Z082_NOCFA), Corynebacterium diphtheriae NCTC 13129(Q6NFT6_CORDI), Saccharomonospora cyanea NA-134(H5XE69_9PSEU), Brachybacterium faecium DSM 4810(C7MBP3_BRAFD), Mycobacterium colombiense CECT 3035(F9QGJ2_9MYCO), Corynebacterium lipophiloflavum DSM 44291(C0XRD8_9CORY), Corynebacterium resistens DSM 45100(F8DZ36_CORRG), Frankia sp CN3(G6HKE2_9ACTO), Streptomyces griseoaurantiacus M045(F3NCP4_9ACTO), Mycobacterium abscessus subsp bolletii(H0IML9_MYCAB), Nakamurella multipartita DSM 44233(C8XGN0_NAKMY), marine actinobacterium PHSC20C1(A4AGR0_9ACTN), Isoptericola variabilis 225(F6FQG9_ISOV2), Jonesia denitrificans DSM 20603(C7R2X0_JONDD), Frankia sp EAN1pec(A8L215_FRASN), Thermobifida fusca(2BMM), Corynebacterium ammoniagenes DSM 20306(D5NXP9_CORAM), Dietzia cinnamea P4(E6J538_9ACTO), Corynebacterium casei UCMA 3821(G7HW23_9CORY), Mycobacterium gilvum PYR-GCK(A4T2Q7_MYCGI), Streptomyces cattleya NRRL 8057(F8JS37_STREN), Gordonia araii NBRC 100433(G7H143_9ACTO), Saccharomonospora paurometabolica YIM 90007(G4J0W0_9PSEU), Streptomyces griseoflavus Tu4000(D9Y0C3_9ACTO), Streptomyces viridochromogenes DSM 40736(D9X9K7_STRVR), Corynebacterium pseudogenitalium ATCC 33035(E2S653_9CORY), Micromonospora sp ATCC 39149(C4RPG9_9ACTO), Corynebacterium glutamicum ATCC 13032(Q8NMW6_CORGL), Stackebrandtia nassauensis DSM 44728(D3Q6N6_STANL), Saccharomonospora glauca K62(H1JG78_9PSEU), Frankia sp CcI3(Q2JDW4_FRASC), Mycobacterium rhodesiae JS60(G4I1H8_MYCRH), Streptomyces hygroscopicus subsp jinggangensis(H2K910_STRHJ), Mycobacterium thermoresistibile ATCC 19527(G7CJC9_MYCTH), Gordonia sputi NBRC 100414(H5U4M1_9ACTO), Mycobacterium leprae(Q9X7B3_MYCLE), Gordonia otitidis NBRC 100426(H5TSE0_9ACTO), Nocardia brasiliensis ATCC 700358(H5RS95_9NOCA), Mycobacterium sp MCS(Q1B5W7_MYCSS), Corynebacterium striatum ATCC 6940(C2CLE9_CORST), Corynebacterium jeikeium K411(Q4JWW0_CORJK), Micromonospora aurantiaca ATCC 27029(D9TAK4_MICAI), Streptomyces violaceusniger Tu 4113(G2NX27_STRVO), Microbacterium testaceum StLB037(E8NDL4_MICTS), Streptomyces sp SPB78(D9UM81_9ACTO), Mycobacterium marinum M(B2HNH8_MYCMM), Frankia sp EuI1c(E3J5P0_FRASU), Saccharomonospora viridis DSM 43017(C7MZW7_SACVD), Streptomyces himastatinicus ATCC 53653(D9WQC1_9ACTO), Gordonia alkanivorans NBRC 16433(F9VZA5_9ACTO), Corynebacterium aurimucosum ATCC 700975(C3PII6_CORA7), Salinispora tropica CNB-440(A4XAM0_SALTO), Mycobacterium smegmatis str MC2(A0R1B8_MYCS2), Streptomyces bingchenggensis BCW-1(D7C3F4_STRBB), Saccharomonospora azurea NA-128(H8GDG3_9PSEU), Mycobacterium sp JDM601(F5YY56_MYCSD), Streptomyces sp e14(D6K0W4_9ACTO), Frankia sp EUN1f(D3D1B2_9ACTO), Gordonia neofelifaecis NRRL B-59395(F1YI30_9ACTO), Gordonia bronchialis DSM 43247(D0LAH0_GORB4), Corynebacterium ulcerans 809(G0CMU9_CORUL), Salinispora arenicola CNS-205(A8M1P3_SALAI), Cellulomonas fimi ATCC 484(F4H5S7_CELFA), Nocardiopsis dassonvillei subsp dassonvillei(D7B3R3_NOCDD), Catenulispora acidiphila DSM 44928(C7Q9L1_CATAD), Nocardia cyriacigeorgica GUH-2(H6R5I9_NOCCG), Gordonia polyisoprenivorans VH2(H6MX45_GORPV), Thermomonospora curvata DSM 43183(D1A9X9_THECD), Geodermatophilus obscurus DSM 43160(D2SCM4_GEOOG), Mobilicoccus pelagius NBRC 104925(H5UVC5_9MICO), Corynebacterium pseudotuberculosis 106-A(G7U1I8_CORPS), Frankia symbiont of Datisca(D3M5P7_9ACTO), Corynebacterium genitalium ATCC 33030(D7WC24_9CORY), Streptomyces scabiei 8722(C9Z5X1_STRSW) |
| LILFLL | 3,37E-02 | | N: Pseudomonas psychrotolerans L19(H0JJY3_9PSED), Oxalobacteraceae bacterium IMCC9480(F1VZN5_9BURK), Pseudomonas fulva 12-X(F6AA16_PSEF1), Pseudomonas fluorescens SBW25(C3K7I0_PSEFS), Xanthomonas oryzae pv oryzae(Q5GXS7_XANOR), Xanthomonas albilineans GPE PC73(D2UCQ4_XANAP), Xanthomonas gardneri ATCC 19865(F0C5U4_9XANT), Xanthomonas campestris pv campestris(B0RR93_XANCB), Xanthomonas vesicatoria ATCC 35937(F0BCB2_9XANT), Ricinus communis(B9TG83_RICCO), Xanthomonas citri pv mangiferaeindicae(H8FET1_XANCI) |
| FIASVW | 1,22E-03 | | P: Burkholderia cenocepacia PC184(A2VXT3_9BURK), Burkholderia gladioli BSR3(F2LCR3_BURGS), Burkholderia ambifaria MEX-5(B1T3N9_9BURK), Burkholderia multivorans ATCC 17616(A9AG64_BURM1), Burkholderia glumae BGR1(C5AFG3_BURGB), Burkholderia vietnamiensis G4(A4JES7_BURVG), Roseobacter sp AzwK-3b(A6FK96_9RHOB), Burkholderia sp(Q39FM2_BURS3) |
| LIASVW | 6,48E-03 | | P: Alicycliphilus denitrificans K601(F4GC46_ALIDK), Acidovorax avenae subsp avenae(F0Q4M9_ACIAP), Polaromonas naphthalenivorans CJ2(A1VL14_POLNA), Lautropia mirabilis ATCC 51599(E7RZ21_9BURK), Bordetella bronchiseptica RB50(Q7WJJ5_BORBR), Nitrosomonas eutropha C91(Q0AIE0_NITEC), Acidovorax citrulli AAC00-1(A1TMH1_ACIAC), Azoarcus sp BH72(A1K1I2_AZOSB), Acidovorax sp NO-1(H0C1D4_9BURK) |
| LIALQV | 6,48E-03 | | N: Plesiocystis pacifica SIR-1(A6G520_9DELT), Cyanothece sp PCC 8802(C7QR53_CYAP0) |
| LIFLLW | 7,95E-06 | | O: Bordetella petrii DSM 12804(A9IJT5_BORPD), Alicycliphilus denitrificans K601(F4GAB4_ALIDK), Nitrosospira multiformis ATCC 25196(Q2Y824_NITMU), Algoriphagus machipongonensis(A3I308_9BACT), Beijerinckia indica subsp indica(B2IE39_BEII9), Acidovorax ebreus TPSY(B9MEH6_ACIET), Acidovorax delafieldii 2AN(C5TD07_ACIDE), Curvibacter putative symbiont of(C9Y8P3_9BURK), Bacteriovorax marinus SJ(E1WZL7_BACMS), Acidovorax sp NO-1(H0BSH7_9BURK) |
| LILLFV | 1,81E-09 | | N: Halomonas sp TD01(F7SSB5_9GAMM), Congregibacter litoralis KT71(A4ACE9_9GAMM) |
| FIASKW | 6,48E-03 | | P: Rhizobium leguminosarum bv trifolii(B5ZXR3_RHILW), Agrobacterium sp ATCC 31749(F5J5A9_9RHIZ), Agrobacterium tumefaciens F2(F7U441_RHIRD), Sinorhizobium medicae WSM419(A6ULS3_SINMW), Agrobacterium tumefaciens CCNWGS0286(G6XXM2_RHIRD), Novosphingobium aromaticivorans DSM 12444(Q2G809_NOVAD), Sinorhizobium fredii NGR234(C3KMT5_RHISN), Rhizobium sp PDO1-076(H4F0B9_9RHIZ), Brevundimonas sp BAL3(B4WCH2_9CAUL), Rhizobium etli CFN 42(Q2KDK7_RHIEC), Rhizobium etli CIAT 652(B3PYD4_RHIE6), Sphingomonas wittichii RW1(A5VGI9_SPHWW), Afipia sp 1NLS2(D6V129_9BRAD), Rhizobium leguminosarum bv viciae(Q1MMQ0_RHIL3), Sinorhizobium meliloti CCNWSX0020(H0FT84_RHIML) |
| LFFFQW | 6,48E-03 | | O: Vitis vinifera(D7SUH5_VITVI), Carica papaya(C4PB37_CARPA), Arabidopsis thaliana(Q946U7_ARATH), Wolffia australiana(H6U807_9ARAE), Physcomitrella patens(A9S385_PHYPA,A9TSV7_PHYPA), Arabidopsis lyrata subsp lyrata(D7M9Q6_ARALL), Selaginella moellendorffii(D8S6W9_SELML), Picea sitchensis(A9NND9_PICSI), Datisca glomerata(Q7Y079_DATGL), Populus trichocarpa(B9H9D4_POPTR), Eutrema halophilum(E4MXS1_THEHA), Glycine max(C6T2S6_SOYBN), Ricinus communis(B9RS68_RICCO), Phaeodactylum tricornutum CCAP 10551(B7G0D0_PHATC) |
