# Supplementary material for: Evolutionary and Functional Relationships in the Truncated Hemoglobin Family
Source: PLoS Comput Biol. 2016 Jan 20;12(1):e1004701. doi: 10.1371/journal.pcbi.1004701 (PMC4720485; doi:10.1371/journal.pcbi.1004701)
Supplement: S3 Table — (DOCX) [file pcbi.1004701.s011.docx]

| ***Organism* trHb – *wild type* / mutant form** | $\boldsymbol{log}\left( \boldsymbol{k}_{\boldsymbol{on}} \right)$ | $\boldsymbol{log}\left( \boldsymbol{k}_{\boldsymbol{on}}\boldsymbol{calc} \right)$ | $\boldsymbol{K}_{\boldsymbol{H}_{\boldsymbol{2}}\boldsymbol{O}}$ | **Active site residues B10-CD1-E7-E11-G8** | **Ref.** ^a^ |
| --- | --- | --- | --- | --- | --- |
| *Mycobacterium tuberculosis*  Mt-O-wt | 5,04 | 5,15 | 3,04E-05 | YYALW | (88) |
| *Mycobacterium tuberculosis*  MtO-YCD1F | 6,43 | 6,62 | 2,28E-04 | YFALW | (88) |
| *Mycobacterium tuberculosis*  Mt-O-WG8F | 7,10 | 7,05 | 2,38E-03 | YYALF | (37) |
| *Mycobacterium tuberculosis*  Mt-O-WG8F-YCD1F | 7,36 | 7,95 | 1,51E-02 | YFALF | (37) |
| *Mycobacterium leprae*  Ml-O-wt | 5,04 | 5,19 | 3,04E-05 | YYALW | (37) |
| *Thermobifida fusca*  Tf-O-wt | 5,95 | 5,48 | 3,04E-05 | YYALW | (89) |
| *Thermobifida fusca*  Tf-O-WG8F | 6,53 | 7,20 | 2,38E-03 | YYALF | (20) |
| *Thermobifida fusca*  Tf-O-WG8F-YB10F-YCD1F | 6,58 | 7,42 | 3,49E-02 | FFALF | (20) |
| *Pseudoalteromonas haloplanktis*  Ph-O-wt | 5,95 | 5,40 | 3,04E-05 | YHIFW | (90) |
| *Agrobacterium tumefaciens*  At-O-wt | 5,30 | 5,19 | 3,04E-05 | YHSFW | (91) |
| *Bacillus Subtilis*  Bs-O-wt | 7,48 | 7,12 | 5,27E-04 | YFTQW | (37) |
| *Geobacillus stearothermophilus*  Gs-O-wt | 7,88 | 6,94 | 5,27E-04 | YFTQW | (37) |
| *Campylobacter jejuni*  Cj-P-wt | 5,96 | 5,33 | 3,04E-05 | YFHVW | (92) |
| *Campylobacter jejuni*  Cj-P-YB10F | 5,08 | 6,08 | 8,33E-05 | FFHVW | (92) |
| *Campylobacter jejuni*  Cj-P-YB10F-HE7L | 7,62 | 8,13 | 3,49E-02 | FFLVW | (92) |
| *Synechocystis*  Syn-N-wt | 8,38 | 7,31 | 9,85E-05 | YFQQV | (84) |
| *Mycobacterium tuberculosis*  Mt-N-wt | 7,40 | 7,00 | 9,85E-05 | YFLQV | (38) |
| *Mycobacterium tuberculosis*  Mt-N-YB10F | 8,73 | 8,19 | 2,82E-03 | FFLQV | (35) |
| *Mycobacterium tuberculosis*  Mt-N-YB10L | 8,79 | 7,87 | 2,82E-03 | LFLQV | (35) |
| *Mycobacterium tuberculosis*  Mt-N-QE11V | 7,51 | 7,18 | 1,38E-04 | YFLVV | (35) |
| *Mycobacterium tuberculosis*  Mt-N-QE11A | 7,57 | 7,43 | 1,38E-04 | YFLAV | (35) |
| *Mycobacterium tuberculosis*  Mt-N-YB10L-QE11V | 9,26 | 9,59 | 8,08E-02 | LFLVV | (35) |
| *Chlamydomonas eugametos*  Ce-N-wt | 6,00 | 6,73 | 2,28E-04 | YFQQV | (49) |
| *Paramecium caudatum*  Pc-N-wt | 7,48 | 7,06 | 9,85E-05 | YFQTV | (49) |
| *Tetrahymena pyriformis*  Tp-N-wt | 6,74 | 6,94 | 2,28E-04 | YFQQI | (39) |
| Sperm whale myoglobin  Mb-wt | 7,20 | 7,22 | 2,28E-04 | LFHV- | (22) |
| Sperm whale myoglobin  Mb-HE7G | 8,18 | 8,20 | 6,52E-03 | LFGV- | (22) |

^a^ The references correspond to the work where the rate constants ($k_{on}$) were measured. Rate constants were usually determined at 20°C.
