# Supplementary material for: Evolutionary and Functional Relationships in the Truncated Hemoglobin Family
Source: PLoS Comput Biol. 2016 Jan 20;12(1):e1004701. doi: 10.1371/journal.pcbi.1004701 (PMC4720485; doi:10.1371/journal.pcbi.1004701)
Supplement: S4 Table — (DOCX) [file pcbi.1004701.s012.docx]

| ***Organism* trHb – *wild type* or mutant form** | $\boldsymbol{log}\left( \boldsymbol{k}_{\boldsymbol{off}} \right)$ | $\boldsymbol{log}\left( \boldsymbol{k}_{\boldsymbol{off}}\boldsymbol{calc} \right)$ | **Active site residues B10-CD1-E7-E11-G8** | **Ref.** ^a^ |
| --- | --- | --- | --- | --- |
| *Mycobacterium tuberculosis*  Mt-O-wt | -2,85 | -1,59 | YYALW | (88) |
| *Mycobacterium tuberculosis*  Mt-O-TrpG8Phe | -0,26 | 0,71 | YYALF | (37) |
| *Bacillus Subtilis*  Bs-O-wt | -2,32 | -1,59 | YFTQW | (37) |
| *Thermobifida fusca*  Tf-O-wt | -1,15 | -1,55 | YYALW | (89) |
| *Mycobacterium tuberculosis*  Mt-N-wt | -0,70 | -1,74 | YFLQV | (38) |
| *Mycobacterium tuberculosis*  Mt-N-TyrB10Leu | 1,65 | 0,07 | AFLQV | (38) |
| *Chlamydomonas eugametos*  Ce-N-wt | -1,85 | -3,09 | YFQQV | (93) |
| *Paramecium caudatum*  Pc-N-wt | 1,40 | 1,29 | YFQTV | (49) |
| *T**etrahymena pyriformis*  Tp-N-wt | -0,74 | -3,09 | YFQQI | (39) |
| *Tetrahymena pyriformis*  Tp-N-GlnE11Ala | 0,69 | -0,51 | YFQAI | (39) |
| *Campylobacter jejuni*  Cj-P-wt | -2,39 | -2,64 | YFHVW | (37) |
| *Campylobacter jejuni*  Cj-P-TrpG8Phe | -1,48 | -1,44 | YFHVF | (26) |
| *Campylobacter jejuni*  Cj-P-HieE7Leu | -3,52 | -3,27 | YFLVW | (37) |
| *Cerebratulus lacteus*  CerHb-wt | 2,26 | 1,04 | YFQTL | (40) |
| *Cerebratulus lacteus*  CerHb-ThrE11Ala | -0,59 | -2,09 | YFQAL | (40) |
| *Cerebratulus lacteus*  CerHb-TyrB10Phe | 2,66 | 2,86 | FFQTL | (40) |
| Soybean leghemoglobin  Lba | 0,70 | 0,68 | YFHLV | (94) |
| Soybean leghemoglobin  Lba-HisF8Gly | 1,18 | 2,86 | YFHLV | (41) |
| *Ascaris lumbricoides*  AscHb | -2,40 | -3,18 | YFQIV | (95) |
| *Methanosarcina acetivorans*  Ma-Pgb | -1,04 | 0,24 | YFVFF | (96) |
| Sperm whale myoglobin  Mb-wt | 1,18 | 1,56 | LFHV- | (22) |
| Sperm whale myoglobin  Mb-HisE7Gly | 3,23 | 3,84 | LFGV- | (22) |

^a^ The references correspond to the work where the rate constants ($k_{off}$) were measured. Rate constants were usually determined at 20°C.
